# Extreme thermal fluctuations from climate change unexpectedly accelerate demographic collapse of vertebrates with temperature-dependent sex determination

**DOI:** 10.1038/s41598-019-40597-4

**Published:** 2019-03-12

**Authors:** Nicole Valenzuela, Robert Literman, Jennifer L. Neuwald, Beatriz Mizoguchi, John B. Iverson, Julia L. Riley, Jacqueline D. Litzgus

**Affiliations:** 1 Department of Ecology, Evolution and Organismal Biology, Iowa State University, Ames, IA 50011 USA; 2 Present Address: Department of Biology, Colorado State University, Fort Collins, CO 80523 USA; 3 Department of Biology, Earlham College, Richmond, Indiana 47374 USA; 4 Present Address: Department of Botany and Zoology, Stellenbosch University, Matieland, 7602, Stellenbosch, Western Cape 7600 South Africa; 5 Department of Biology, Laurentian University, Sudbury, Ontario P3E 2C6 Canada

## Abstract

Global climate is warming rapidly, threatening vertebrates with temperature-dependent sex determination (TSD) by disrupting sex ratios and other traits. Less understood are the effects of increased thermal fluctuations predicted to accompany climate change. Greater fluctuations could accelerate feminization of species that produce females under warmer conditions (further endangering TSD animals), or counter it (reducing extinction risk). Here we use novel experiments exposing eggs of Painted Turtles (*Chrysemys picta*) to replicated profiles recorded in field nests plus mathematically-modified profiles of similar shape but wider oscillations, and develop a new mathematical model for analysis. We show that broadening fluctuations around naturally male-producing (cooler) profiles feminizes developing embryos, whereas embryos from warmer profiles remain female or die. This occurs presumably because wider oscillations around cooler profiles expose embryos to very low temperatures that inhibit development, and to feminizing temperatures where most embryogenesis accrues. Likewise, embryos incubated under broader fluctuations around warmer profiles experience mostly feminizing temperatures, some dangerously high (which increase mortality), and fewer colder values that are insufficient to induce male development. Therefore, as thermal fluctuations escalate with global warming, the feminization of TSD turtle populations could accelerate, facilitating extinction by demographic collapse. Aggressive global CO_2_ mitigation scenarios (RCP2.6) could prevent these risks, while intermediate actions (RCP4.5 and RCP6.0 scenarios) yield moderate feminization, highlighting the peril that insufficient reductions of greenhouse gas emissions pose for TSD taxa. If our findings are generalizable, TSD squamates, tuatara, and crocodilians that produce males at warmer temperatures could suffer accelerated masculinization, underscoring the broad taxonomic threats of climate change.

## Introduction

Unlike most animals that possess sex chromosomes and whose gonadal development is relatively less sensitive to environmental conditions, the sexual fate of many reptiles and some fish is triggered by the temperatures experienced during development^1,2^ (TSD), whereas others have mixed mechanisms of sex determination^3^. While understanding the evolution of sex-determining mechanisms remains a scientific challenge^4^, it is generally recognized that TSD renders species vulnerable to extinction if they are unable to counter the sex-biasing effect of rapid contemporary climate change by adapting via plastic or evolutionary responses in their thermal sensitivity or behavior^5,6^. Yet, general awareness of the threats posed by climate change to TSD taxa is restricted to the negative effect of higher average global temperature. Indeed, the consequences of accentuated thermal fluctuations predicted to accompany rising mean temperatures^7,8^ remain unknown because no study has explored how greater thermal oscillations in natural nests affect TSD sex ratios under realistic scenarios.

Most TSD turtles produce males at lower incubation temperatures and females at higher temperatures^1,2^. Consequently, extinction risk escalates when feminization reaches levels that impair population growth, because extreme male scarcity limits reproduction and sex ratio bias promotes the loss of genetic variation. Such extreme feminization resulting from contemporary global warming has already been documented in sea turtle populations exposed to rising average temperatures^9^. Extreme sex ratio biases are also predicted to afflict other TSD turtles, many of which are already endangered^10^. Additionally, some lizards, crocodilians, and the tuatara develop into males at higher temperatures^1,2^, and their populations may be even more vulnerable to extinction because global warming induces masculinization, and female scarcity can induce demographic collapse faster than female excess. The effects of climate change can be complex, because the influence of skewed sex ratios on population dynamics may be mediated by the species’ mating system^11^ or longevity^12^, and the effect of warming temperatures on TSD sex ratios may be ameliorated by behavioral variation in nest-site choice and the timing of nesting within populations^13^. Less understood is the effect of thermal fluctuations on TSD sex ratios^5,14^. Also unknown is whether the predicted accentuation of temperature fluctuations expected to occur seasonally with global warming^7,8^ will ultimately amplify or antagonize the effect of higher average temperatures. Additional sources of thermal variation affect TSD taxa, such as in sea turtles whose nests may be deep enough to lack daily thermal variation, but which experience fluctuating temperatures associated with precipitation events that are also predicted to be altered by climate change^15^.

**Figure 1:**
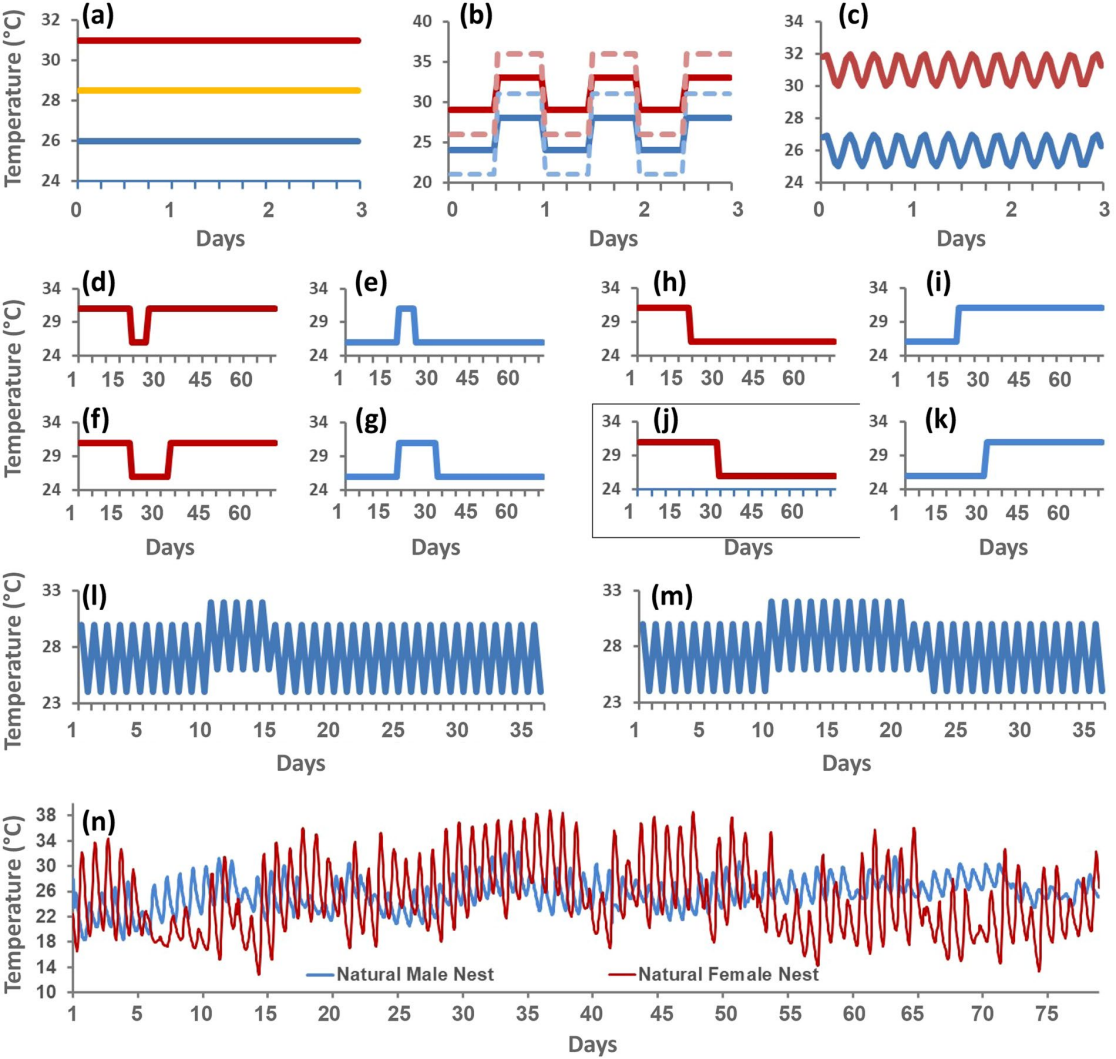
Exemplar thermal profiles used in studies of temperature-dependent sex determination and natural nest profiles. (**a**) Constant incubation conditions [examples illustrate temperatures that produce only males (blue), only females (red) or 1:1 sex ratios (yellow) in the Painted Turtles used here]. (**b**) Oscillating temperature conditions alternating high-low values that deviate more or less (dashed versus solid lines) from a male- (blue/light-blue) or female-producing (red/light-red) average temperature (e.g.^14^). (**c**) Cosine-model of thermal oscillations around a stationary temperature to model simple diel thermal fluctuations (e.g.^53^). (**d**–**k**) Shift-twice (panels d–g) and shift-once (panels h–k) experiments between a female-producing (high; in red) or male-producing (low; in blue) temperature, which are used to identify the thermosensitive period by delaying the time when eggs are transferred between temperatures or the duration of exposure to the second temperature (e.g.^24,54^). (**l**,**m**) Shift-twice experiments under simple oscillations to study the effect of heat waves of varying duration under constant amplitude but varying average temperature^19^. (**n**) Natural nest profiles recorded in the field that produce only males (blue) or only females (red) and whose complexity is not captured by any of the simpler experimental profiles used thus far as illustrated in panels (a–m) (e.g. this study, or^24^).

The effects of temperature fluctuation remain enigmatic because experimental approaches so far have been restricted to using simplistic thermal profiles, such as uniform sinusoidal cycles or high/low alternating temperatures (Fig. 1a–m). These simplistic experiments do not capture the complexity of the incubation conditions found in nature, where diel fluctuations may not follow a uniform cycle and may include seasonal or sub-seasonal trends (Fig. 1n)^14,16–20^. Therefore, research thus far may have obscured the potential for mitigating or exacerbating responses to contemporary climate change that can only be revealed under more realistic experimental conditions.

Here we tested whether accentuated thermal fluctuations around natural nest profiles offset the negative impact that contemporary global warming might have on the persistence of TSD species, by examining embryonic development in the Painted Turtle (*Chrysemys picta*), a TSD reptile^21^ lacking sex chromosomes^22^. Earlier work using simple 12 hr-high/12 hr-low temperature oscillation profiles of increasing amplitude (Fig. 1b) demonstrated that Painted Turtles undergo sex reversal when artificial incubation temperatures diverge significantly from average values that produce exclusively males or females when the temperature is constant^14^. This earlier study revealed the unappreciated effect that increased thermal variance may have on sex determination^14^, which could have important ecological and evolutionary consequences provided that similar effects were experienced by nests in nature. Namely, if rising average temperature alone induces feminization of TSD turtles but global warming is accompanied by more marked thermal oscillations within natural nests that have a masculinizing effect, then these effects could counter each other, slowing down the feminization of TSD population, and thus decelerating the concomitant demographic collapse. However, natural nests experience more complex thermal oscillations than used in all previous experimental studies (Fig. 1n versus Fig. 1a–m), such that the ecological relevance of previous observations^14,19,23^ remain unclear. Here we use a novel and ecologically-relevant experimental approach, replicating and modifying thermal profiles recorded in natural nests. We show that instead of having a moderating effect, increased thermal variation may accelerate the rate at which natural TSD turtle populations could become feminized by climate change. Our data revealed that larger oscillations alter the balance between embryonic development accrued above and below the pivotal temperature by altering the exposure of eggs to temperatures that vary in their potency to sustain development and to trigger male or female development. We also found that geographic populations differed in their responses to identical natural-nest incubation conditions. These interpopulation differences could have a potentially alleviating effect, provided they reflect heritable variation. But adaptive responses would only be possible if they are not precluded by the fragmentation of natural habitats or impeded by the speed at which contemporary global temperatures are changing. Our findings, if applicable to other TSD reptiles, including those that produce males at warmer temperatures, underscore the potentially devastating effect of climate change at a broader taxonomic scale than previously appreciated.

## Results

### Natural nest profiles replicated in the lab

**Figure 2:**
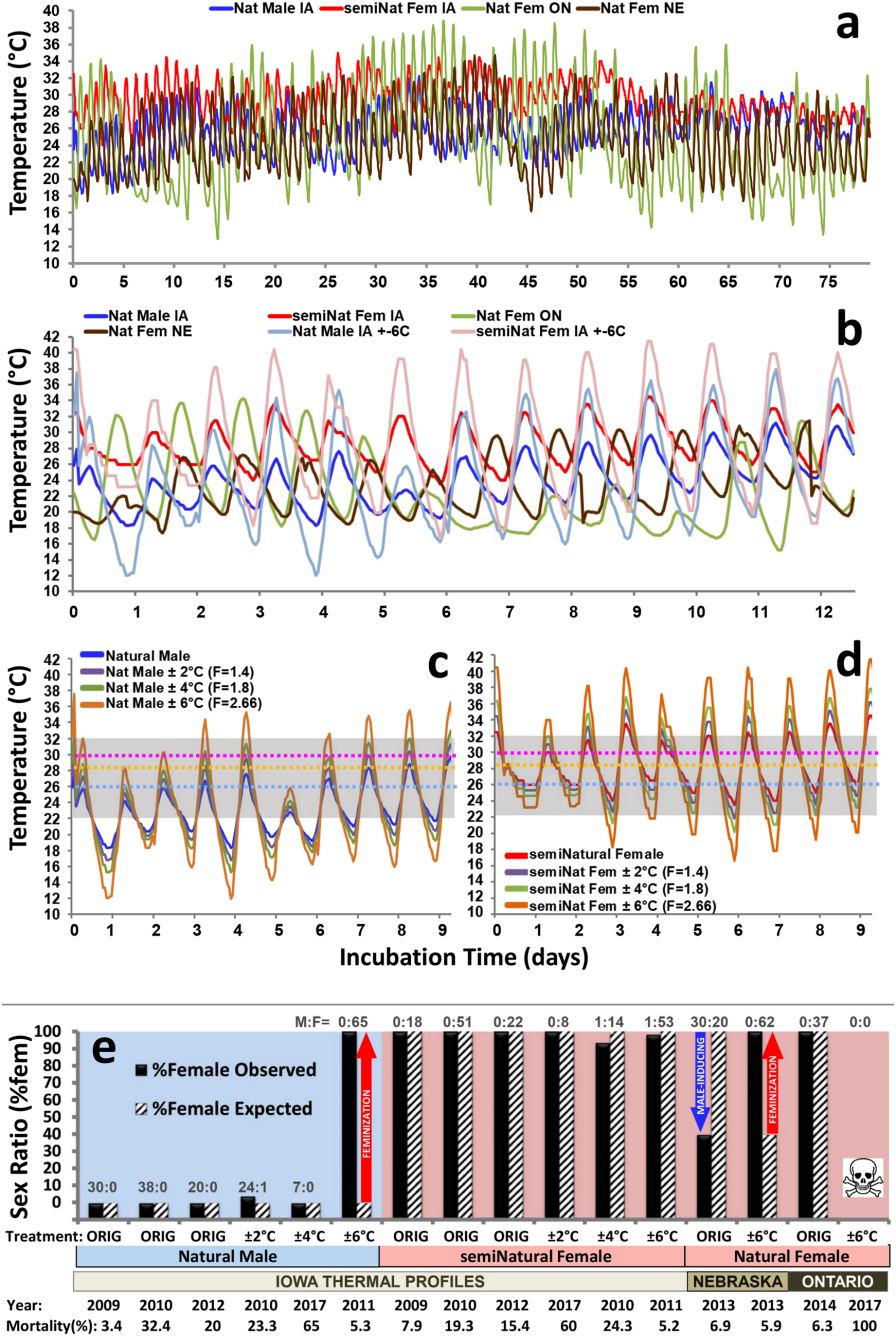
Thermal regimes used in this study to incubate eggs of Painted Turtles, *Chrysemys picta* and resulting sex ratios. (**a**) Baseline thermal profiles recorded in the field (hourly) from a male-producing natural nest in Iowa (NatMale-IA in blue), from female-producing natural nests in Ontario (NatFem-ON in green) and Nebraska (NatFem-NE in brown), and female-producing profile (semiNatFem-IA in red) generated by adding 5 °C to each record from the NatMale-IA profile to simulate increased overall mean global temperature. (**b**) Comparison of excerpts of the hourly baseline profiles from panel (a) plus the most extreme ±6 °C affine-transformed profiles from Iowa (NatMale-IA ±6 °C in light blue and semiNatFem-IA ±6 °C in pink). (**c**,**d**) Excerpts of the Iowa baseline profiles (NatMale-IA in blue and semiNatFem-IA in red) illustrating the full range of affine transformations aimed to increase thermal variance by approximately ±2 °C (purple lines), by ~±4 °C (green lines), and by ~±6 °C (orange lines). These excerpts depict how the transformations preserve the general shape of the original profiles (transformed profiles maintain the inflection points and the timing of the daily maxima and minima). Gray areas demarcate the optimal temperature range for development in Painted Turtles^14^. Dashed lines = constant temperature that produces 100% females (pink), 100% males (light blue) or 1:1 sex ratios (yellow). (**e**) Sex ratios obtained in the lab from eggs incubated under the profiles recorded in field nests or affine-transformed as described in panels (c,d) above (see text for details), which were replicated in programmable incubators. Red and blue arrows indicate significant deviations from expected sex ratios (i.e. sex ratios recorded from field nests) in the direction of the arrow (Chi^2^_(df=1)_ test, *P* < 0.00001 in all three cases). Abbreviations: Nat Male IA = male-producing profile recorded in a field nest in Iowa; semiNat Fem IA = semi-natural female-producing profile generated as described for panel (a) above. Nat Fem NE = profile recorded in a natural nest in Nebraska which produced 100% females in Nebraska but induced mostly males using eggs from an Iowa farm. Nat Fem ON = female-producing profile recorded in a natural nest in Ontario (Canada) which also produced 100% females using eggs from an Iowa farm. ORIG = original profiles; ±2 °C, ±4 °C, ±6 °C = amplitude of thermal variance added to original profiles by the affine transformations. M:F = number of males and females obtained in each lab experiment.

A thermal profile recorded hourly inside a natural nest of Painted Turtles (*Chrysemys picta*) in Iowa that produced only males (NatMale-IA; Fig. 2a) was replicated in the lab and all eggs exposed to this thermal profile produced the expected 100% males (Fig. 2e). Likewise, when this NatMale-IA profile was shifted by adding 5 °C to each recorded temperature to simulate the predicted feminizing effect of a rising mean temperature if global greenhouse gas emissions are not curtailed^8^, eggs incubated in the lab produced 100% females (semiNatFem-IA profile in Fig. 2b). An Iowa all-female natural profile was not used because all natural and experimental nests monitored in Iowa over multiple years (2006–2010) produced male-biased sex ratios (likely due to the relatively colder temperatures experienced during the nesting season at the monitored locations over those years – see Supplementary Information).

### High thermal variance induces feminization or embryo mortality

These NatMale-IA and semiNatFem-IA profiles were then modified by an affine mathematical transformation that increased the variance of the profile (by approximately ±2 °C, ±4 °C, ±6 °C) while preserving its general shape (maintaining, in general, the inflection points and timing of daily maxima/minima – see Fig. 2b–d). Exposing eggs to such modified incubation conditions had mixed results depending on the amplitude of the oscillations. First, we found that the tighter fluctuations of ±2 °C and ±4 °C around both the male-producing (NatMale-IA) and female-producing (semiNatFem-IA) profiles, had virtually no effect, such that the observed sex ratios matched the expected sex ratios (Fig. 2e). However, wider fluctuations of ±6 °C around the male-producing natural profile (NatMale-IA ±6 °C) feminized all embryos, whereas embryos from the semiNatFem-IA ±6 °C treatment were virtually unaffected and developed as females (Fig. 2e).

Next, because stretching the NatMale-IA and semiNatFem-IA profiles by ±6 °C caused unidirectional sex reversal (i.e. feminization and not masculinization), we examined the generality of this effect by testing whether adding ±6 °C to natural nest profiles that produced 100% females in the wild would also lack a sex reversal effect as for the semiNatFem-IA ±6 °C treatment. For this, we used a natural profile recorded from a population of Painted Turtles from the Nebraska Sandhills and one from a population from Algonquin Provincial Park in Ontario (Canada), both of which produced exclusively females at those sites (NatFem-NE and NatFem-ON, respectively) (Fig. 2a,e). Notably, we found that when using Iowa eggs, the Ontario NatFem-ON profile replicated in the lab induced 100% females, whereas the Nebraska NatFem-NE profile induced male-biased development (40% females) (Fig. 2e), demonstrating significant interpopulation differences in the responses to identical natural-nest incubation conditions. When the ±6 °C affine transformation was applied to these thermal profiles and Iowa eggs were incubated under those settings, the modified Nebraska profile (NatFem-NE ±6 °C) elicited full feminization just as the NatMale-IA ±6 °C treatment did, whereas the modified Ontario profile (NatFem-ON ±6 °C) resulted in 100% embryonic mortality (Fig. 2e).

### Cumulative heat under thermal oscillations determines sex ratios

**Figure 3:**
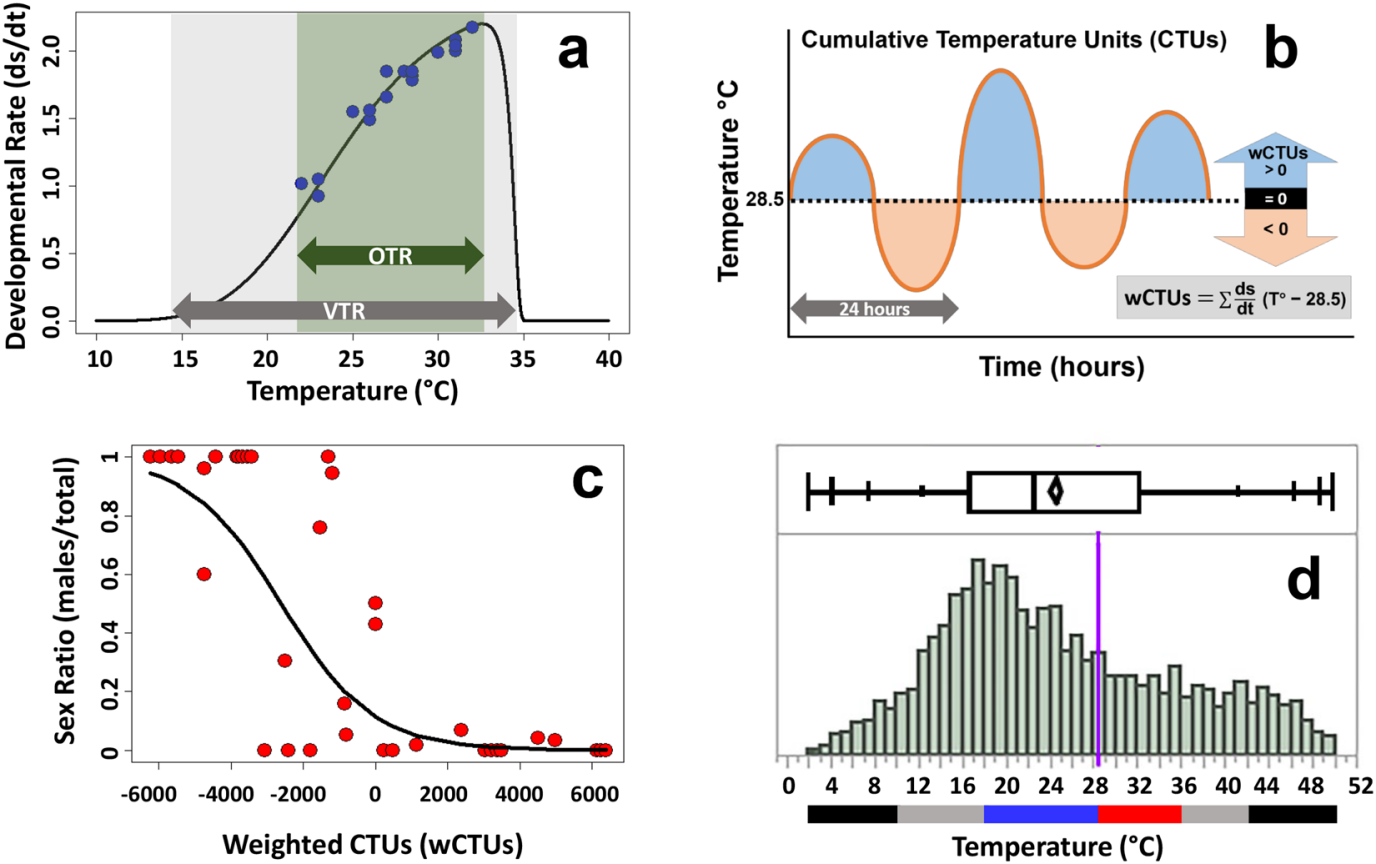
Components of the novel wCTU model of development and sex determination for the TSD Painted Turtle, *Chrysemys picta*. (**a**) Developmental rate by temperature function obtained by applying the nlCTE model^14^ to incubation data of Painted Turtles (see text for details). OTR = Optimal Temperature Range for *C*. *picta*^14^. VTR = Viable Temperature Range (temperatures that induce a developmental rate > 0). Blue dots denote actual (rather than predicted) developmental rate from constant incubation experiments to illustrate de good fit of the model. (**b**) Cumulative Temperature Units (CTUs) sensu^24^ above (blue areas) and below (orange areas) the pivotal temperature (dotted line) which is 28.5 °C in *C*. *picta* used in this study. Each hourly temperature is multiplied by the developmental rate it induces (calculated from panel *a* to obtain the weighted CTUs (wCTUs). (**c**) Sex ratios of *C*. *picta* as a function of wCTUs, and fitted logistic regression (black line). Sex ratio data derive from constant and simplistic fluctuations from^14,25,^ plus data from the novel experiments from this study. (**d**) Density distribution and quantile plot of temperatures from the thermal profile NatFem-ON ±6 °C that resulted in 100% mortality. The 25% to 75% quantile box depicts the average temperature (diamond) and the median temperature line. The bottom bars illustrate temperature ranges within the OTR that induce males (blue) or females (red), the ranges outside the OTR but within the VTR (gray), and the temperatures lower or higher than the most extreme values in all other profiles (black). Note that most temperatures of the NatFem-ON ±6 °C profile fell below the pivotal temperature (purple vertical line) and a substantial portion fell in the gray areas where development is retarded or in the black range of temperatures that may be lethal, perhaps explaining the high mortality of this treatment.

To explain the sex ratios produced by the various thermal treatments used in this study we developed a new mathematical model by calculating the weighted Cumulative Temperature Units (wCTUs) for each profile (Fig. 3a,b). wCTUs correspond to the integrated time (by hour) that embryos were exposed to temperatures above and below the pivotal temperature (the value that produces 1:1 sex ratio, i.e. 28.5 °C in the *C*. *picta* used here; modified from^24^; Fig. 3b). Under this model, each hourly temperature is weighted by the developmental rate it induces, which was calculated by fitting a non-linear Constant Temperature Equivalent (nlCTE) model to the incubation data from this study, supplemented with data from constant and artificially-simple fluctuating temperature experiments (see Fig. 1a–m) from^14,25^ (Fig. 3a). This novel wCTU model provided a good fit to the sex ratio data for *C*. *picta* as indicated by the highly significant logistic regression of male proportion against wCTUs (Z = −15.15, *P* < 1 × 10^−15^) (Fig. 3c). We observed that thermal oscillations alter the balance between the proportion of development that takes place at feminizing versus masculinizing temperatures by exposing embryos to values outside the viable thermal range (VTR, Fig. 3a) which contribute little or nil to development. This in turn alters how much of development is realized at temperatures within the VTR that vary in their potency to sustain development and in their potency to differentially induce male or female determination/differentiation (which can be overlapping processes in TSD taxa^26^).

## Discussion

Our novel experimental approach represents the first ecologically-relevant test of the effects of increased thermal variance in wild nests (as predicted by climate change) on sex determination in TSD vertebrates, beyond the effect of rising mean temperature. We accomplished this by exposing developing embryos to various thermal profiles, some recorded directly within nests in the field and some modified using mathematical transformations. This range of conditions simulates not only increased average temperature but also accentuated fluctuations as predicted from climate change scenarios. Prior to our study, it remained untested whether thermal oscillations could mitigate the detrimental effects of rising global temperature, or exacerbate these effects such that we might expect faster collapse of TSD populations in the future. This knowledge gap remained because previous studies compared the effect of constant incubation temperatures to simplistic thermal fluctuations in the laboratory that differed greatly from natural nest conditions (Fig. 1)^14,16–19^, whereas the thermal oscillations used herein were based on real nest profiles (compare Figs 1n and 2a–d with Fig. 1b–m). Incubating eggs under thermal conditions modified by our mathematical transformation showed that the feminization of TSD turtles accelerates drastically as thermal fluctuations increase in natural nests, highlighting an unanticipated threat of climate change for TSD vertebrates.

Namely, we found that sexual development of embryos from the ‘stretched’ NatMale-IA and semiNatFem-IA profiles (Fig. 2c,d) was unaffected if the added variation was narrow (restricted to ±2 °C and ±4 °C; Fig. 2e), whereas complete feminization was induced by wider fluctuations around otherwise male-producing conditions (NatMale-IA ±6 °C) but no reversal was observed around female-producing conditions (semiNatFem-IA ±6 °C; Fig. 2e). These results resemble previous observations in which a milder simple fluctuation of ±3 °C around a mean of 26 °C or 31 °C had no effect on sexual development of Painted Turtles, but a wider fluctuation of ±5 °C induced sex reversal (and greater mortality around the 31 °C average)^14^. Notably, this previous sex reversal effect was bidirectional^14^ providing some hope that males produced by high variance could alleviate somewhat the feminization from global warming. Increasing the amplitude of simple fluctuations around average values that produce a single sex in the TSD lizard *Amphibolurus muricatus* also had a sex reversal effect^27^, and wider oscillations in natural nests of *Crocodylus moreletii* correlated positively with female-biased sex ratios^28^, indicating that these effects may be taxonomically widespread among TSD reptiles. Notice, however, that except for our present study, the oscillating profiles used in all previous experiments differed greatly from real nest profiles (see Fig. 1) and thus failed to mimic the conditions currently experienced by natural populations, or those forecasted under climate change. Unfortunately, when using ecologically-relevant conditions (Fig. 2e), as we do here, only a feminizing effect was detected, suggesting that there may be no good news after all.

This unidirectional pattern of sex reversal was further confirmed by our observations that the NatFem-NE profile (which produced 60% males using Iowa eggs) and the NatMale-IA profile (which produced 100% males using Iowa eggs) induced full feminization when stretched by ±6 °C (NatFem-NE ±6 °C and NatMale-IA ±6 °C treatments) (Fig. 2e), whereas stretching the female-producing profile from Ontario (NatFem-ON ±6 °C) caused 100% mortality (Fig. 2e). Given that the original NatFem-ON profile was characterized by a wider range of temperatures than the IA and NE profiles (Fig. 2b), the lethality of the NatFem-ON ±6 °C treatment suggests a limit to the thermal variation that is tolerable by developing embryos. This agrees with observations in other TSD turtles^29–31^, echoing the concern that extreme temperatures imperil population viability via aggravated mortality. However, it should be noted that egg inviability was particularly high across all treatments that year (2017) for unknown reasons (Fig. 2e).

All our observations are explained by our wCTU model which provided a good fit to the sex ratios produced under a wide range of treatments (Fig. 3c), from constant temperature and artificially simplistic oscillations (Fig. 1a–m) to the natural conditions and profiles modified by our mathematical affine transformation (Fig. 2). Further, the wCTU model accounted for the effect of all viable temperatures (including those outside of the linear range of development). Importantly, the wCTU model performed better than the alternative models on which it is based, such as the CTE^16^ or CTU^24^ models (which apply only to temperatures in the linear developmental phase), and even the non-linear CTE model (nlCTEs)^14^, all of which provided a poor fit for the full range of sex ratios when used separately (results not shown). The wCTU results revealed the potential mechanistic basis of the effects of thermal fluctuations on sex ratios and mortality in general, and of predicted climate change in particular. Namely, female (or male) determination occurs when a larger proportion of the development takes place at temperatures above (or below) the constant-pivotal temperature (the value that produces 1:1 sex ratios), which is ~28.5 °C for *C*. *picta* used here (Fig. 3). And temperatures within the viable thermal range (VTR) have a stronger potency to induce femaleness or maleness the further they deviate from the pivotal value. Importantly, this potency is amplified (or dampened) by the effect that temperatures within the VTR have on developmental rate (Fig. 3a). For instance, because higher temperatures within the VTR accelerate development, 1 hr spent 2 °C above the pivotal temperature has a stronger feminizing effect than the masculinizing effect of 1 hr spent 2 °C below the pivotal temperature because more development accrues at the warmer than at the colder temperature^16^ each hour. The wCTU model explains the feminization we observed under the NatMale-IA ±6 °C or NatMale-NE ±6 °C treatments. Namely, these profiles exposed embryos to temperatures in the lower range of, or below, the VTR which retard development (and contributed little or nil to accrued development). These treatments also exposed embryos to temperatures above the pivotal value with greater feminizing-potency, because they accelerate development (and thus contribute disproportionately to accrued development) (Fig. 2b). A similar phenomenon would describe why virtually only females developed under experiments with accentuated oscillations around the semiNatFem-IA female-producing thermal profile, where most embryonic development accrued during exposure to temperatures above the pivotal value. The wCTU model also explains the masculinization and higher (albeit partial) mortality reported when *C*. *picta* eggs were incubated under cycles of 12 hrs at 36 °C followed by 12 hrs at 26 °C (average 31 °C)^14^, because 36 °C is predicted to induce little to no development (Fig. 3a) such that most embryogenesis ensued under the male-inducing 26 °C. Notably, embryos incubated under severe oscillations could experience extreme temperatures with little potency to induce sex determination and that could even be lethal because they deviate too far outside the VTR, or because exposure is too prolonged or too frequent. This appears to have caused the high mortality under the NatFem-ON ±6 °C treatment (Fig. 3d) which exposed embryos to temperatures that exceeded the highest (42 °C) and lowest (10 °C) temperatures of all other profiles (which may be lethal when sustained for too long or repeatedly), and to temperatures that depress or halt development even if they are not lethal for short periods (11–20 °C and 36–41 °C). Our wCTU model may be applicable to other TSD taxa, provided that it is modified by adjusting the parameters to species- or population-specific values.

Importantly, our observations also revealed profound differences that exist among geographically distant populations in their sex ratio response to identical thermal regimes encountered in nature. Such interpopulation variation in the sex ratios induced by identical incubation temperatures (i.e., their reaction norms) has been reported previously in turtles [examples reviewed in^32^] and other TSD reptiles. These accounts include variation among conspecific populations of the turtles *Chrysemys picta*, *Chelydra serpentina*, *Graptemys pseudogeographic*a, and potentially *Pseudemys concinna*, *Terrapene carolina*^32–34^ and *Natator depressus*^35^, as well as the lizard *Niveoscincus ocellatus*^36^. In Painted Turtles a lower pivotal temperature (also called threshold temperature) was identified in southern populations (Tennessee) compared to northern ones (Wisconsin)^33^. Thus, given that the parental adults from the Iowa turtle farm used in our study purportedly derive in large part from Minnesota, the response we observed to the Nebraska NatFem-NE thermal profile supports the trend of higher pivotal temperature with latitude^33^, whereas the response to the Ontario NatFem-ON thermal profile run counter to a West-to-East decline in pivotal temperature also proposed for Painted Turtles^32^.

The interpopulation variation in the response to identical natural incubation conditions that we observed here may be of paramount importance. If such heterogeneity among populations is heritable (as has been reported for Painted Turtles^37^), the underlying genetic variation may permit the survival of some populations of TSD turtles that are able to produce males at high average global temperatures. Further, the male-biased sex ratios observed at the monitored field sites in Iowa during 2006–2010 indicate that colder spells within or between years at some nesting localities (see Supplementary Information) represent male-producing bouts that could also attenuate the overall feminizing trend of climate change at those locations. The same would be true for sea turtles that produce more males when precipitation events cool down nests, except that precipitation may become rarer at some sea turtle nesting sites with climate change^15^, or if the thermal fluctuations induced by precipitation resemble those observed in our study and become feminizing. Male production at some locations could benefit other populations if males could disperse between localities. Unfortunately, species-level recovery from such surviving source populations (from which an influx of males or an influx of heritable thermosensitive variation could derive) may be impossible due to habitat fragmentation that prevents recolonization of areas of extirpation without human intervention, especially for highly endangered species^38,39^. Habitat fragmentation can also preclude populations from shifting their distributional ranges on their own to track optimal temperatures at higher latitudes. On the other hand, intraspecific variation in the response of the gene regulatory network controlling sexual development to incubation temperature exists in Painted Turtles^40^. If heritable, such genetic diversity could also provide raw material for natural selection and consequently, for populations of TSD turtles to evolve adaptively to the challenges of climate change. Whether inter- or intra-population variation may have permitted TSD turtles to survive extinction during past episodes of climate change by fine-tuning their TSD mechanism or by evolving sex chromosomes^41^ remains unclear. It is also unknown whether such adaptive responses to ensure the future survival of TSD taxa are possible today given the fast pace of contemporary environmental degradation^8,42^ and the typically slower tempo of adaptive evolution, particularly in turtles which have long generation times^23,43,44^.

In conclusion, previous research using simplistic incubation conditions (Fig. 1b) suggested that larger fluctuations in incubation temperature might counteract the detrimental effect that contemporary climate change could have on TSD turtles by triggering male development under warmer average temperatures^14^. However, our study using ecologically-relevant experiments, underscores the perils for the persistence of TSD taxa associated with the lack of action to reduce contemporary greenhouse gas emissions to levels that lower or reverse projected climate change. First, higher average global temperatures as predicted for the year 2100 if no additional efforts are made to curtail gas emissions (Representative Concentration Pathway – RCP8.5)^8^ could fully feminize natural nests that under today’s conditions produce 100% males. Such dire predictions have become a reality for some loggerhead sea turtles populations^9^ and single-sex populations are projected to afflict a broad array of TSD reptiles in the near future, including not only turtles, but also lizards, crocodilians, and tuatara (e.g.^45–47^).

For Painted Turtles the drastic effects detected here are preventable only under aggressive mitigation scenarios (RCP2.6), while intermediate actions (RCP4.5 and RCP6.0 scenarios) will increase average temperatures to levels that result in more moderate feminization. Importantly however, any climate change that increases the thermal variance in TSD turtle nests, even if the average temperature remains intact, could have a detrimental feminizing effect. Thus, the larger fluctuations expected to accompany global warming can have a synergistic effect and actually accelerate the rate of feminization of TSD turtle populations. This would put them at greater risk of extinction than they are already, a risk that exacerbates the effects of other factors such as habitat degradation and anthropogenic exploitation, which combined have rendered this charismatic lineage the most endangered vertebrate group on earth^10^.

Finally, given that sex-reversal effects have now been detected in both turtles and lizards^14,27^, further research is urgently needed to test the hypothesis that the responses we document here are taxonomically widespread, which is partially supported by the consistent responses reported across various TSD turtles to simpler thermal oscillations^16–18,48^. Furthermore, studies that incorporate natural nest thermal oscillations under realistic climate change scenarios across varied taxa would help illuminate the mysteries of TSD and the future of the many reptiles that rely on this environmental mechanism of sex determination.

## Methods

Freshly-laid eggs of Painted Turtles (*Chrysemys picta*) obtained from Iowa turtle farms were incubated in moistened-sand^14^ following standard protocols^49^. Programmable incubators were set to replicate (a) hourly thermal profiles recorded from natural nests in the field using Dallas Semiconductor iButton™ dataloggers with 0.5 °C precision (Fig. 2a,b), or (b) thermal profiles modified by an affine mathematical transformation that increases the variance of the profile (by ±2 °C, ±4 °C, ±6 °C) while preserving its general shape (Fig. 2b–d). Thermal profiles were affine-transformed using the equation

1 $${{\rm{T}}}_{{\rm{new}}}=[({\rm{T}}-{\rm{M}})\ast {\rm{F}}]+{\rm{M}}$$

where T is the temperature recorded every hour in a nest, M is the daily mean temperature of that nest, and F is a scalar factor. An F = 1.4 stretched the thermal profile by approximately ±2 °C, F = 1.8 by ~±4 °C, and F = 2.3 by ~±6 °C. This affine transformation preserves the shape of the thermal profiles by generally maintaining the inflection points and timing of daily maxima/minima (see Fig. 2b–d). These treatments bracket the variance of our previous experiments in which sex reversals were detected^14^. Hatchlings were raised for three months to ensure that sexual differentiation was complete prior to sexing by gonadal inspection^14^. Deviations from expected sex ratios were assessed using a Chi^2^_(df=1)_ test, and significance assessed at an α = 0.05. Experiments were conducted between 2009 and 2017 as eggs and incubators were available. All procedures were carried out in accordance with relevant guidelines and regulations, and were approved by the IACUC of Iowa State University.

The following thermal profiles were utilized (all incubation profiles are included in the Supplementary Information). First, we recorded hourly thermal profiles inside natural nests laid by Painted Turtles at an Iowa turtle farm using iButton dataloggers placed in the bottom, middle, and top of the egg chamber (two dataloggers per level), and documented their resulting sex ratios (Fig. 2). Care was taken to carefully and quickly remove the eggs, maintaining the integrity of the egg chamber, placing the dataloggers, positioning the eggs back in their original orientation, and covering the nest with the original substrate. iButtons are small enough that their effect on nest temperature is negligible (as evidenced by the fact that the profiles recorded in the middle of the egg chamber produce identical sex ratios when replicated in the laboratory). We tested the predicted feminizing effect of a rising mean temperature if global gas emissions are not curtailed^8^, by adding 5 °C to each recorded temperature of a natural profile that produced 100% males (Iowa natural male profile: NatMale-IA) which generated a semi-natural profile that induced 100% females (semiNatFem-IA) (Fig. 2a,b). Because eggs incubated under these two incubation profiles produced the expected 100% males and 100% females (respectively) during three consecutive years when replicated in the lab, such that no sex ratio biases were detected that could be attributable to year-to-year differences in the source of eggs or incubator performance (Chi^2^_(df=1)_ > 3.84, *P* > 0.05 in all cases), all other treatments were conducted in single years.

The unidirectional feminization effect of a ±6 °C affine transformation was then tested using natural nest profiles that produced 100% females in a Nebraska Sandhills population and in an Algonquin Provincial Park population (Ontario, Canada), NatFem-NE and NatFem-ON respectively (Fig. 2a,e). No 100%-female natural profile was available from Iowa because all natural and experimental nests monitored over multiple years (2006–2010) produced male-biased sex ratios (perhaps in response to relatively colder temperatures experienced during the nesting season over those years –  see Supplementary Information).

To explain the sex ratios produced by the various thermal profiles, we devised a novel mathematical model by calculating the weighted Cumulative Temperature Units (wCTUs) for each profile. wCTUs were defined as

2 $${\rm{wCTUs}}={\sum }^{}\frac{{\rm{ds}}}{{\rm{dt}}}\,({\rm{T}}-{{\rm{T}}}_{{\rm{piv}}})$$

that is, the sum of the hourly temperature deviations from the pivotal temperature (T = 28.5 °C in our case) (i.e., CTUs *sensu*^24^) multiplied by the developmental rate elicited by each temperature (d_s_/d_t_). The d_s_/d_t_ value for each temperature (expressed as a percentage per day) was calculated by fitting the non-linear Constant Temperature Equivalent (nlCTE) model^14^ (Fig. 3a) to all available incubation data from our lab and additional data^14,25^, using the function *devara* in the *DEVAR* 2.0.1 software^50^. In particular,

3 $$\frac{{\rm{ds}}}{{\rm{dt}}}={b}_{1}\,{10}^{-{v}^{2}(1-{b}_{5}+{b}_{5}{v}^{2})}$$

where

4 $$v=(u+{e}^{{b}_{4}u})/{c}_{2};$$

5 $$u=(T-{b}_{3})/({b}_{3}-{b}_{2})-{c}_{1};$$

6 $${c}_{1}=1/[1+0.28{b}_{4}+0.72\,\mathrm{ln}(1+{b}_{4})],$$

and

7 $${c}_{2}=1+[{b}_{4}/(1+1.5{b}_{4}+0.39{{b}_{4}}^{2})].$$

*T* represents the hourly incubation temperature, *b*_1_ is the maximal developmental rate, *b*_3_ is the temperature at which *b*_1_ occurs, *b*_2_ is the temperature at which developmental rate is *b*_1_/10, *b*_4_ controls how sharply d_s_/d_t_ approaches 0 at the lower temperatures, and *b*_5_ controls the asymmetry of d_s_/d_t_^50^. The best fit was given by *b*_*1*_ = 2.2, *b*_2_ = 18 °C, *b*_3_ = 32.5 °C, *b*_4_ = 14, and *b*_5_ = 0.6 (Fig. 3a), which correctly estimated the developmental zero around 14 °C as previously reported for *C*. *picta*^51^. A logistic regression of sex ratio by wCTUs was then calculated using the function *glm* in R version 3.5.1^52^.

## Supplementary information

Supplemental Information
