# Supplementary material for: Extreme thermal fluctuations from climate change unexpectedly accelerate demographic collapse of vertebrates with temperature-dependent sex determination
Source: Sci Rep. 2019 Mar 12;9:4254. doi: 10.1038/s41598-019-40597-4 (PMC6414666; doi:10.1038/s41598-019-40597-4)
Supplement: Supplementary file 1 — Supplemental Information [file 41598_2019_40597_MOESM1_ESM.pdf]

Supplemental Information – Scientific Reports 2019

Extreme thermal fluctuations from climate change unexpectedly accelerate demographic collapse of vertebrates with temperature-dependent sex determination

Nicole Valenzuela, Robert Literman, Jennifer L. Neuwald, Beatriz Mizoguchi, John B. Iverson, Julia L. Riley, and Jacqueline D. Litzgus.

Figure S1: Monthly average temperatures (°C) for two locations were painted turtle nests were monitored, Ames (IA) and Birmingham (IA) (to which the closest weather station is Fairfield, IA). The panels below illustrate monthly average temperatures for June (panel A), July (panel B) and August (panel C) for the years 1893-2018 obtained from the Iowa Environmental Mesonet Climodat reports, which provide monthly summaries of daily climate record data from The National Weather Service [NWS] Cooperative Observer Program [COOP] (https://mesonet.agron.iastate.edu/climodat/index.phtml?network=IACLIMATE&station=IA0200&report=16). Averages across years are shown as dotted lines for Ames and dashed lines for Fairfield. Yellow boxes denote the period in 2006-2010 when nests were monitored in the field in Iowa and which produced male-biased sex ratios exclusively. While the period 2006-2010 is relatively cold (particularly in July), the monthly averages during those years are not unprecedented, as illustrated by the distribution of temperatures for the years 1893-2018 (panel D). Thus, it is expected that male-producing years are not an uncommon occurrence at this locations.

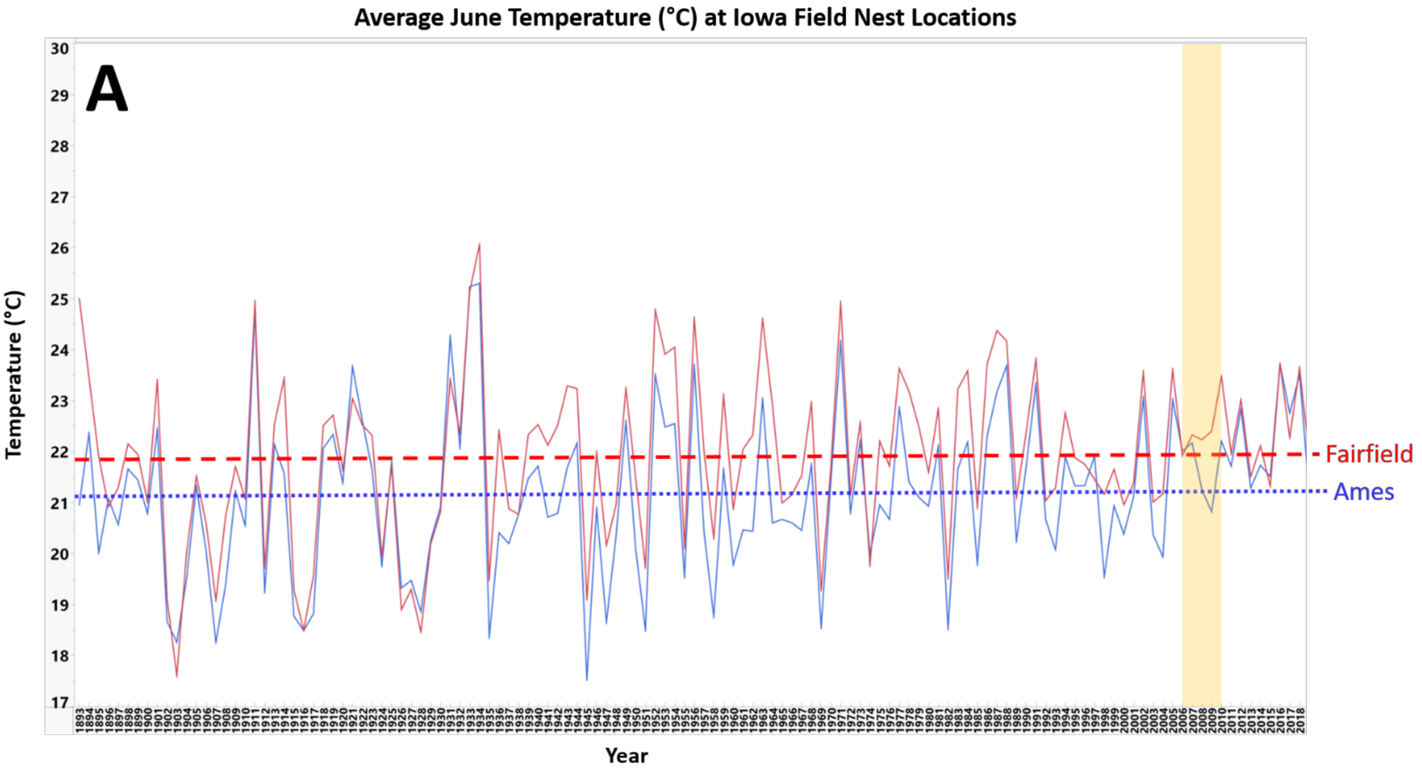

Average July Temperature (°C) at Iowa Field Nest Locations

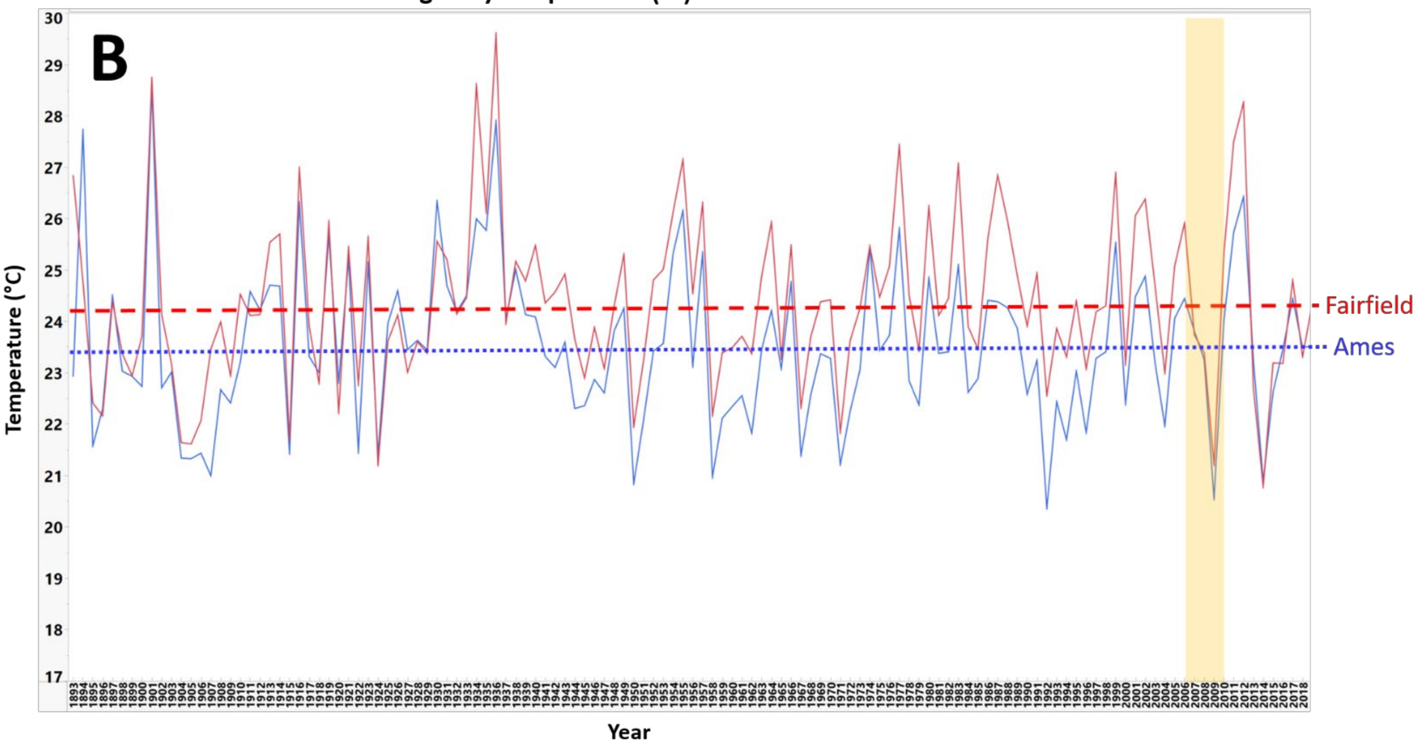

Average August Temperature (°C) at Iowa Field Nest Locations

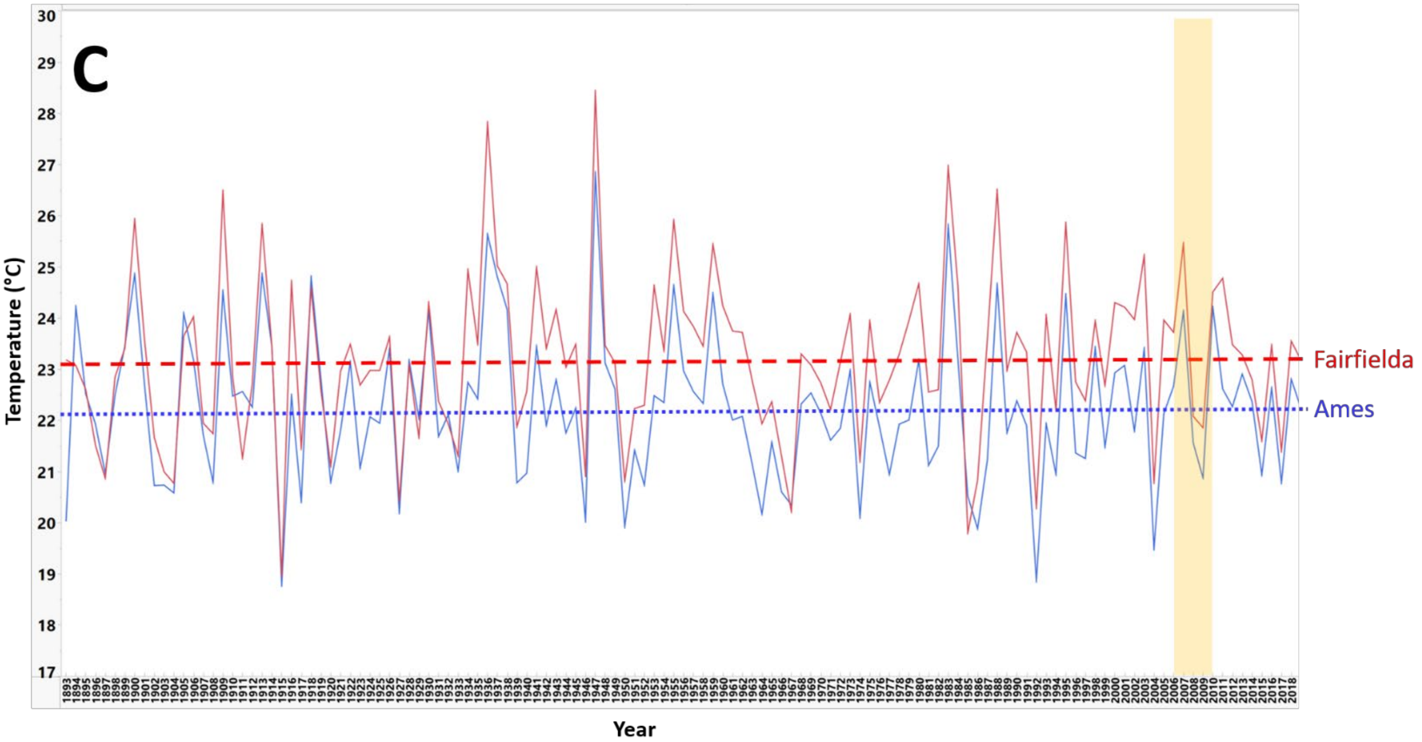

D

Year	JUN-Ames	JUL-Ames	AUG-Ames	JUN-Fairfield	JUL-Fairfield	AUG-Fairfield
2006	22.0	24.4	22.7	21.9	25.9	23.7
2007	22.2	23.8	24.1	22.3	23.7	25.5
2008	21.3	23.3	21.6	22.2	23.4	22.1
2009	20.8	20.5	20.9	22.4	21.2	21.9
2010	22.2	24.0	24.2	23.5	25.3	24.5

Distributions

JUN-Ames | JUL-Ames | AUG-Ames | JUN-Fairfield | JUL-Fairfield | AUG-Fairfield

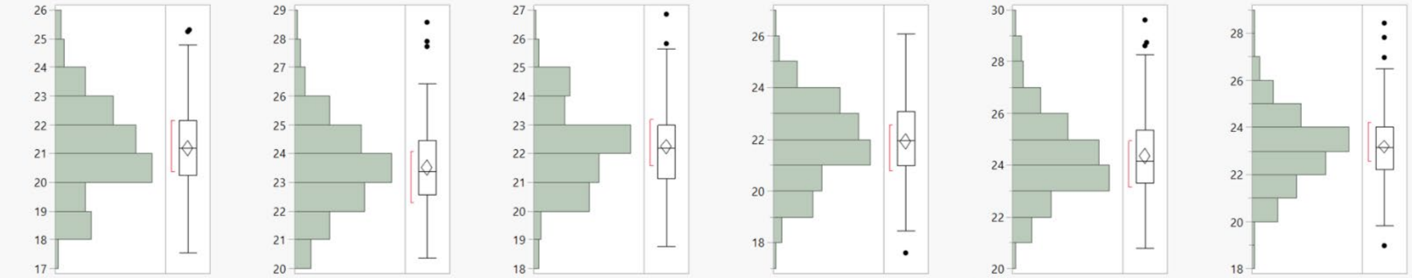

Quantiles

		JUN-Ames	JUL-Ames	AUG-Ames	JUN-Fairfield	JUL-Fairfield	AUG-Fairfield
100.0%	maximum	25.296296296	28.55734767	26.836917563	26.055555556	29.596774194	28.422939068
99.5%		25.296296296	28.55734767	26.836917563	26.055555556	29.596774194	28.422939068
97.5%		24.679398148	27.489247312	25.499775986	24.997916667	28.542562724	26.880824373
90.0%		23.216666667	25.565412186	24.290322581	23.842592593	26.519713262	25.079749104
75.0%	quartile	22.166666667	24.453405018	22.999551971	23.055555556	25.351702509	24.030017921
50.0%	median	21.166666667	23.373655914	22.190860215	21.949074074	24.16218638	23.158602151
25.0%	quartile	20.229166667	22.567204301	21.140232975	20.960648148	23.299731183	22.208781362
10.0%		18.807407407	21.433691756	20.487455197	19.557407407	22.291218638	21.069892473
2.5%		18.277083333	20.850134409	19.558243728	18.562037037	21.280241935	20.229166667
0.5%		17.537037037	20.367383513	18.781362007	17.601851852	20.779569892	18.96953405
0.0%	minimum	17.537037037	20.367383513	18.781362007	17.601851852	20.779569892	18.96953405

Summary Statistics

	JUN-Ames	JUL-Ames	AUG-Ames	JUN-Fairfield	JUL-Fairfield	AUG-Fairfield
Mean	21.169459	23.515603	22.231538	21.907701	24.349149	23.175314
Std Dev	1.5567629	1.5287577	1.4420368	1.6074095	1.6293513	1.5970606
Std Err Mean	0.1386875	0.1361926	0.1284669	0.1431994	0.1451541	0.1422775
Upper 95% Mean	21.443939	23.785145	22.48579	22.191111	24.636428	23.456899
Lower 95% Mean	20.894979	23.246061	21.977287	21.624292	24.061871	22.89373
N	126	126	126	126	126	126

Hourly thermal profiles used in the present study

Sex Ratio Wild Nest:		NatMale	NatMale	NatMale	NatMale	semiNat Fem	semiNat Fem	semiNat Fem	semiNat Fem	NatFem	NatFem	NatFem	NatFem
Original Recorded At:		IA	IA	IA	IA	IA	IA	IA	IA	NE	NE	ON	ON
Added Variance:		Original	±2°C	±4°C	±6°C	Original	±2°C	±4°C	±6°C	Original	±6°C	Original	±6°C
Day	Hour	Temp	Temp	Temp	Temp	Temp	Temp	Temp	Temp	Temp	Temp	Temp	Temp
1	1	25.9	27.4	32.6	32.2	32.5	34.4	36.4	40.5	20	19.8	22.4	20.3
1	2	27.9	30.2	24.6	37.6	32.5	34.4	36.4	40.5	20	19.9	21.3	17.7
1	3	23.5	24.1	26.1	25.9	31.5	33	34.6	37.8	19.9	19.7	20.3	15.4
1	4	24.3	25.2	27	28	30	30.9	31.9	33.8	19.7	19.3	19.4	13.4
1	5	24.8	25.9	27.9	29.3	29.5	30.2	31	32.5	19.5	18.8	18.6	11.5
1	6	25.3	26.6	28.8	30.7	27.5	27.4	27.4	27.2	19.3	18.3	17.9	10
1	7	25.8	27.3	28.1	32	28	28.1	28.3	28.5	19.1	17.9	17.2	8.4
1	8	25.4	26.7	26.1	30.9	28	28.1	28.3	28.5	18.8	17.2	16.7	7.1
1	9	24.3	25.2	24.5	28	28	28.1	28.3	28.5	18.7	16.9	16.5	6.8
1	10	23.4	23.9	23.6	25.6	27.5	27.4	27.4	27.2	18.6	16.7	17.2	8.4
1	11	22.9	23.2	22.5	24.3	27.5	27.4	27.4	27.2	18.6	16.8	19	12.5
1	12	22.3	22.4	21.4	22.7	27	26.7	26.5	25.9	18.8	17.1	21.7	18.6
1	13	21.7	21.6	20.5	21.1	27	26.7	26.5	25.9	19.2	18	24.6	25.2
1	14	21.2	20.9	19.6	19.7	26.5	26	25.6	24.5	19.5	18.8	27	30.8
1	15	20.7	20.2	18.9	18.4	26.5	26	25.6	24.5	20	19.8	29.2	36
1	16	20.3	19.6	18	17.4	26.5	26	25.6	24.5	20.7	21.5	30.7	39.4
1	17	19.8	18.9	17.1	16	26.5	26	25.6	24.5	21.3	22.9	32.1	42.6
1	18	19.3	18.2	16.2	14.7	26	25.3	24.7	23.2	21.8	24	32.2	42.7
1	19	18.8	17.5	16.2	13.4	26	25.3	24.7	23.2	22	24.5	31.9	42
1	20	18.8	17.5	15.3	13.4	26	25.3	24.7	23.2	22	24.6	31.1	40.3
1	21	18.3	16.8	15.3	12	26	25.3	24.7	23.2	22	24.5	29.8	37.3
1	22	18.3	16.8	15.5	12	26	25.3	24.7	23.2	20.6	21.4	28.2	33.7
1	23	18.4	16.9	15.5	12.3	26	25.3	24.7	23.2	20.5	21.1	26.7	30.1
1	24	18.4	16.9	17	12.3	26	25.3	24.7	23.2	20.7	21.6	25.3	26.9
2	25	19.1	18.1	18.5	14.8	26	25.4	24.7	23.3	20.9	19.4	24.1	21.9
2	26	19.9	19.2	19.2	17	26.5	26.1	25.6	24.7	20.8	19.2	23	19.4
2	27	20.3	19.8	20.3	18	27	26.8	26.5	26	20.5	18.6	22.1	17.3
2	28	20.9	20.6	22.4	19.6	28	28.2	28.3	28.7	20.3	18.1	21.2	15.3
2	29	22.1	22.3	24.6	22.8	28.5	28.9	29.2	30	20	17.5	20.4	13.5
2	30	23.3	24	26.2	26	29.5	30.3	31	32.6	19.8	16.9	19.8	12.1
2	31	24.2	25.2	26	28.4	30	31	31.9	34	19.5	16.2	19.3	10.9
2	32	24.1	25.1	25.3	28.1	30	31	31.9	34	19.2	15.6	19	10.4
2	33	23.7	24.5	24.8	27.1	30	31	31.9	34	18.9	15	19	10.2
2	34	23.4	24.1	24.1	26.3	29	29.6	30.1	31.3	17.7	12.2	19.6	11.7
2	35	23	23.5	23.7	25.2	28.5	28.9	29.2	30	17.3	11.3	21.2	15.3
2	36	22.8	23.3	22.8	24.7	28.5	28.9	29.2	30	17.9	12.5	23.2	20
2	37	22.3	22.6	22.3	23.3	28	28.2	28.3	28.7	19.5	16.2	25.9	26
2	38	22	22.1	21.9	22.5	28	28.2	28.3	28.7	21.2	20.2	27.9	30.6
2	39	21.8	21.9	21.2	22	27.5	27.5	27.4	27.3	22.2	22.5	30.2	36
2	40	21.4	21.3	21	21	27	26.8	26.5	26	24.1	26.8	32.1	40.4
2	41	21.3	21.2	20.3	20.7	27	26.8	26.5	26	25.7	30.5	33.2	43
2	42	20.9	20.6	20.1	19.6	26.5	26.1	25.6	24.7	26.6	32.7	33.7	44.1
2	43	20.8	20.5	19.4	19.4	26.5	26.1	25.6	24.7	26.9	33.4	33.6	43.8
2	44	20.4	19.9	19.4	18.3	26.5	26.1	25.6	24.7	26.8	33.1	32.6	41.6
2	45	20.4	19.9	19.4	18.3	26	25.4	24.7	23.3	26.4	32.1	31.2	38.3
2	46	20.4	19.9	19.7	18.3	26	25.4	24.7	23.3	25.7	30.5	29.7	34.9
2	47	20.6	20.2	20.5	18.8	26	25.4	24.7	23.3	24.9	28.7	28.4	31.9
2	48	21	20.7	20.2	19.9	26	25.4	24.7	23.3	24.1	26.7	27.1	28.9
3	49	21.5	20.9	22.2	18.8	26	25.4	24.9	23.6	23.3	23.3	25.9	25
3	50	22.6	22.4	24.2	21.7	26	25.4	24.9	23.6	22.7	21.8	24.9	22.7
3	51	23.7	23.9	26	24.7	27	26.8	26.7	26.3	22.1	20.6	23.9	20.5
3	52	24.7	25.3	27.2	27.3	28.5	28.9	29.4	30.3	21.7	19.6	23.2	18.8
3	53	25.4	26.3	27.9	29.2	30	31	32.1	34.3	21.3	18.8	22.4	17.1
3	54	25.8	26.9	27.9	30.3	31	32.4	33.9	36.9	21	18	21.6	15.2
3	55	25.8	26.9	27.2	30.3	31.5	33.1	34.8	38.2	20.7	17.3	20.9	13.5
3	56	25.4	26.3	26.3	29.2	31.5	33.1	34.8	38.2	20.3	16.5	20.2	12.1
3	57	24.9	25.6	25.6	27.9	30.5	31.7	33	35.6	19.9	15.6	19.9	11.2
3	58	24.5	25.1	24.9	26.8	30	31	32.1	34.3	19.7	15	20.1	11.8

3	59	24.1	24.5	24.3	25.7	29	29.6	30.3	31.6	19.7	15.1	21.3	14.5
3	60	23.8	24.1	24	24.9	28.5	28.9	29.4	30.3	20.1	16	23.4	19.4
3	61	23.6	23.8	23.4	24.4	28	28.2	28.5	28.9	21.4	19	26	25.3
3	62	23.3	23.4	22.7	23.6	27.5	27.5	27.6	27.6	23	22.7	28.7	31.6
3	63	22.9	22.8	22.5	22.5	27	26.8	26.7	26.3	24.9	26.9	31.2	37.2
3	64	22.8	22.7	21.8	22.3	26.5	26.1	25.8	24.9	26.4	30.3	32.9	41.2
3	65	22.4	22.1	21.5	21.2	26	25.4	24.9	23.6	27	31.8	33.9	43.5
3	66	22.2	21.8	20.7	20.7	25.5	24.7	24	22.3	27.2	32.3	34.3	44.4
3	67	21.8	21.3	19.8	19.6	25	24	23.1	21	27.1	32.1	34	43.8
3	68	21.3	20.6	18.9	18.3	25	24	23.1	21	26.7	31.1	32.9	41.2
3	69	20.8	19.9	18.4	17	24.5	23.3	22.2	19.6	26.2	29.9	31.2	37.2
3	70	20.5	19.5	18.2	16.2	24	22.6	21.3	18.3	25.9	29.3	29.5	33.3
3	71	20.4	19.3	18.6	15.9	24.5	23.3	22.2	19.6	25.5	28.3	27.9	29.6
3	72	20.6	19.6	20.8	16.4	25.5	24.7	24	22.3	24.8	26.8	26.4	26.3
4	73	21.4	21.1	22.5	20.2	27	26.1	25.1	23.1	24.2	25.9	25.1	25
4	74	22.3	22.4	24.3	22.6	29	28.9	28.7	28.4	23.8	24.9	23.8	22.1
4	75	23.3	23.8	26.4	25.3	30.5	31	31.4	32.4	23.1	23.3	22.7	19.7
4	76	24.5	25.5	28	28.5	32	33.1	34.1	36.4	22.3	21.4	21.7	17.3
4	77	25.4	26.7	29.5	30.9	33	34.5	35.9	39.1	21.5	19.7	20.8	15.2
4	78	26.2	27.8	30.4	33	33.5	35.2	36.8	40.4	21	18.4	20	13.3
4	79	26.7	28.5	28.9	34.3	33	34.5	35.9	39.1	20.5	17.3	19.2	11.6
4	80	25.9	27.4	27.1	32.2	32.5	33.8	35	37.8	20	16.2	18.6	10.2
4	81	24.9	26	25.5	29.6	32	33.1	34.1	36.4	19.5	15.1	18.2	9.3
4	82	24	24.8	24.4	27.2	31	31.7	32.3	33.8	19.4	14.8	18.7	10.4
4	83	23.4	23.9	23.4	25.6	30.5	31	31.4	32.4	19.4	14.8	20.1	13.6
4	84	22.8	23.1	22.5	24	30	30.3	30.5	31.1	19.9	15.9	21.9	17.6
4	85	22.3	22.4	21.4	22.6	29.5	29.6	29.6	29.8	21.4	19.4	24	22.5
4	86	21.7	21.5	20.5	21	29	28.9	28.7	28.4	23	23.1	26.3	27.8
4	87	21.2	20.8	19.8	19.7	28.5	28.2	27.8	27.1	24.8	27.2	28.6	33
4	88	20.8	20.3	18.9	18.6	28	27.5	26.9	25.8	26.4	30.9	30.5	37.5
4	89	20.3	19.6	18	17.3	27.5	26.8	26	24.5	26.8	31.8	31.9	40.8
4	90	19.8	18.9	17.1	16	27	26.1	25.1	23.1	25.2	28.2	32.7	42.5
4	91	19.3	18.2	16.5	14.7	27	26.1	25.1	23.1	26.1	30.3	32.7	42.6
4	92	19	17.8	16.2	13.9	26.5	25.4	24.2	21.8	26.2	30.5	32	40.9
4	93	18.8	17.5	15.3	13.3	26.5	25.4	24.2	21.8	25.3	28.4	30.6	37.8
4	94	18.3	16.8	15.6	12	26.5	25.4	24.2	21.8	24.6	26.7	29.1	34.2
4	95	18.5	17.1	17.4	12.5	26.5	25.4	24.2	21.8	23.7	24.8	27.5	30.6
4	96	19.5	18.5	19.1	15.2	27.5	26.8	26	24.5	22.9	23	26.1	27.3
5	97	20.8	19.9	21.8	17.2	29	29.4	29.7	30.5	22.1	22.1	24.8	25.9
5	98	22.3	22	23.9	21.2	30.5	31.5	32.4	34.5	21.4	20.7	23.7	23.3
5	99	23.5	23.7	26.6	24.4	31.5	32.9	34.2	37.1	21	19.6	22.7	21
5	100	25	25.8	29	28.4	31	32.2	33.3	35.8	20.5	18.4	21.8	19
5	101	26.3	27.6	30.8	31.9	30.5	31.5	32.4	34.5	20.1	17.5	21	17.2
5	102	27.3	29	31.3	34.5	30	30.8	31.5	33.1	19.7	16.6	20.4	15.7
5	103	27.6	29.5	30.4	35.3	30	30.8	31.5	33.1	19.4	15.9	19.8	14.4
5	104	27.1	28.8	28.4	34	30	30.8	31.5	33.1	19	15.2	19.3	13.3
5	105	26	27.2	26.8	31.1	29.5	30.1	30.6	31.8	18.7	14.5	19	12.7
5	106	25.1	26	25.4	28.7	29.5	30.1	30.6	31.8	18.5	14	19.4	13.6
5	107	24.3	24.8	24.5	26.5	29	29.4	29.7	30.5	18.5	13.8	20.1	15
5	108	23.8	24.1	23.4	25.2	28.5	28.7	28.8	29.2	18.9	14.9	21.6	18.4
5	109	23.2	23.3	22.5	23.6	28	28	27.9	27.8	20.1	17.6	23.8	23.6
5	110	22.7	22.6	21.6	22.3	27.5	27.3	27	26.5	21.6	21	25.7	28.1
5	111	22.2	21.9	20.7	20.9	27.5	27.3	27	26.5	22.4	22.9	28.3	34
5	112	21.7	21.2	20	19.6	27	26.6	26.1	25.2	23.5	25.4	29.5	36.7
5	113	21.3	20.6	19.1	18.6	26.5	25.9	25.2	23.8	24.8	28.3	29.3	36.3
5	114	20.8	19.9	18.2	17.2	26.5	25.9	25.2	23.8	25.9	31	28.7	35
5	115	20.3	19.2	18	15.9	26	25.2	24.3	22.5	26.5	32.4	27.9	33.1
5	116	20.2	19.1	17.3	15.6	25.5	24.5	23.4	21.2	26.5	32.2	27.1	31.2
5	117	19.8	18.5	17.1	14.6	25	23.8	22.5	19.8	26	31.2	26.2	29.2
5	118	19.7	18.4	17.3	14.3	25	23.8	22.5	19.8	25.3	29.6	25.5	27.5
5	119	19.8	18.5	17.8	14.6	25	23.8	22.5	19.8	24.5	27.6	24.8	25.9
5	120	20.1	19	19.7	15.4	26	25.2	24.3	22.5	23.6	25.6	24.1	24.3
6	121	20.3	20	20.3	19.2	27	26.7	26.5	25.9	22.8	24.1	23.4	26.2
6	122	20.6	20.4	21	19.9	28	28.1	28.3	28.6	22.1	22.4	22.7	24.6
6	123	21	21	21.7	21	29.5	30.2	31	32.5	21.4	21	22.1	23.1

6	124	21.4	21.6	22.6	22.1	30.5	31.6	32.8	35.2	20.9	19.7	21.6	21.8
6	125	21.9	22.3	23.3	23.4	31.5	33	34.6	37.9	20.4	18.6	20.9	20.3
6	126	22.3	22.8	23.5	24.5	32	33.7	35.5	39.2	19.9	17.6	20.2	18.8
6	127	22.4	23	24.1	24.7	32	33.7	35.5	39.2	19.6	16.7	19.6	17.4
6	128	22.7	23.4	24.2	25.5	32	33.7	35.5	39.2	19.3	16.1	19.2	16.4
6	129	22.8	23.5	23.9	25.8	31	32.3	33.7	36.5	19.1	15.6	19.1	16.2
6	130	22.6	23.2	23.3	25.3	30	30.9	31.9	33.9	19	15.3	19.5	17.1
6	131	22.3	22.8	22.4	24.5	29	29.5	30.1	31.2	19.1	15.6	20.2	18.8
6	132	21.8	22.1	21.7	23.1	28.5	28.8	29.2	29.9	19.7	16.9	20.9	20.3
6	133	21.4	21.6	21.4	22.1	27.5	27.4	27.4	27.2	20.7	19.4	21.4	21.5
6	134	21.2	21.3	20.8	21.5	27	26.7	26.5	25.9	21.7	21.6	22.2	23.3
6	135	20.9	20.9	19.9	20.7	26.5	26	25.6	24.6	22.3	23.1	22.8	24.7
6	136	20.4	20.2	19.6	19.4	26	25.3	24.7	23.2	22.8	24	22.9	24.9
6	137	20.2	19.9	19	18.9	25.5	24.6	23.8	21.9	23.5	25.8	22.6	24.1
6	138	19.9	19.5	18.8	18.1	25	23.9	22.9	20.6	24.5	28.1	22.4	23.7
6	139	19.8	19.3	18.3	17.8	24.5	23.2	22	19.2	25.3	29.9	22.2	23.3
6	140	19.5	18.9	17.9	17	24.5	23.2	22	19.2	24.8	28.9	21.9	22.7
6	141	19.3	18.6	17.8	16.5	24	22.5	21.1	17.9	24.4	27.9	21.7	22.3
6	142	19.2	18.5	18.5	16.2	23.5	21.8	20.2	16.6	23.8	26.5	21.4	21.4
6	143	19.6	19	19.7	17.3	24	22.5	21.1	17.9	23.1	24.9	20.9	20.3
6	144	20.3	20	19.5	19.2	25	23.9	22.9	20.6	22.5	23.4	20.3	19
7	145	21.3	20.4	22	17.5	26.5	26	25.5	24.5	22	19.7	19.7	21.4
7	146	22.7	22.3	24	21.2	28	28.1	28.2	28.5	21.5	18.5	19.3	20.6
7	147	23.8	23.9	26.1	24.1	29.5	30.2	30.9	32.5	21.1	17.6	18.9	19.7
7	148	25	25.6	28.5	27.3	30.5	31.6	32.7	35.1	20.7	16.8	18.6	19
7	149	26.3	27.4	29.2	30.8	31.5	33	34.5	37.8	20.4	16.1	18.4	18.4
7	150	26.7	27.9	29.4	31.8	32.5	34.4	36.3	40.5	20.2	15.7	18.2	18.1
7	151	26.8	28.1	29.7	32.1	32	33.7	35.4	39.1	20	15.2	18	17.7
7	152	27	28.4	27.9	32.6	32	33.7	35.4	39.1	19.8	14.8	17.9	17.4
7	153	26	27	27	30	30.5	31.6	32.7	35.1	19.7	14.5	17.9	17.2
7	154	25.5	26.3	25.8	28.6	30	30.9	31.8	33.8	19.5	14	17.9	17.2
7	155	24.8	25.3	24.9	26.8	29	29.5	30	31.1	19.4	13.7	18	17.5
7	156	24.3	24.6	24.1	25.4	28.5	28.8	29.1	29.8	19.9	15	18	17.5
7	157	23.9	24	23.2	24.4	27.5	27.4	27.3	27.2	21.7	19.1	18.1	17.8
7	158	23.4	23.3	22.3	23	27	26.7	26.4	25.8	23.6	23.4	18.2	18.1
7	159	22.9	22.6	22.2	21.7	26.5	26	25.5	24.5	25.3	27.3	18.3	18.3
7	160	22.8	22.5	21.4	21.5	26	25.3	24.6	23.2	26.8	30.9	18.3	18.3
7	161	22.4	21.9	20.9	20.4	25.5	24.6	23.7	21.8	28.2	34.1	18.6	19
7	162	22.1	21.5	20.4	19.6	25.5	24.6	23.7	21.8	29.1	36.1	18.8	19.4
7	163	21.8	21.1	19.6	18.8	25	23.9	22.8	20.5	29.5	37	18.8	19.4
7	164	21.4	20.5	19.5	17.7	24.5	23.2	21.9	19.2	29.6	37.2	18.4	18.5
7	165	21.3	20.4	18.9	17.5	24	22.5	21	17.8	29.2	36.2	18.2	18
7	166	21	20	19.5	16.7	24	22.5	21	17.8	28.3	34.2	18	17.5
7	167	21.3	20.4	21.1	17.5	24	22.5	21	17.8	27.2	31.6	17.8	17.1
7	168	22.2	21.6	22.4	19.9	25	23.9	22.8	20.5	26	29	17.7	16.8
8	169	23.3	22.9	24.2	21.5	26.5	25.7	24.9	23.1	24.4	24.4	17.5	15.2
8	170	24.3	24.3	26.2	24.2	28	27.8	27.6	27.1	23.4	22.2	17.5	15.1
8	171	25.4	25.8	28	27.1	29	29.2	29.4	29.8	22.7	20.4	17.4	15
8	172	26.4	27.2	29.8	29.8	30.5	31.3	32.1	33.8	22	18.9	17.4	14.8
8	173	27.4	28.6	31.1	32.4	31.5	32.7	33.9	36.4	21.4	17.6	17.4	14.8
8	174	28.1	29.6	31.4	34.3	32.5	34.1	35.7	39.1	20.9	16.4	17.4	14.8
8	175	28.3	29.9	30.9	34.8	32.5	34.1	35.7	39.1	20.4	15.3	17.3	14.7
8	176	28	29.4	29.1	34	32	33.4	34.8	37.8	20	14.3	17.3	14.7
8	177	27	28	27.5	31.4	31	32	33	35.1	19.5	13.2	17.6	15.4
8	178	26.1	26.8	26.6	29	30.5	31.3	32.1	33.8	19.2	12.6	18	16.4
8	179	25.6	26.1	25.3	27.6	30	30.6	31.2	32.5	19.1	12.3	18.5	17.4
8	180	24.9	25.1	24.4	25.8	29.5	29.9	30.3	31.1	19.7	13.7	19	18.5
8	181	24.4	24.4	23.5	24.4	29	29.2	29.4	29.8	21.6	18	19.3	19.3
8	182	23.9	23.7	22.6	23.1	28.5	28.5	28.5	28.5	23.6	22.6	19.8	20.4
8	183	23.4	23	22.3	21.8	28	27.8	27.6	27.1	25.5	27	21.1	23.4
8	184	23.2	22.7	21.5	21.2	27.5	27.1	26.7	25.8	27.4	31.2	21.6	24.4
8	185	22.8	22.2	20.8	20.2	27	26.4	25.8	24.5	28.8	34.5	21.9	25.3
8	186	22.4	21.6	20.5	19.1	26.5	25.7	24.9	23.1	29.8	36.8	22	25.5
8	187	22.2	21.3	19.7	18.6	26	25	24	21.8	30.3	38	22.1	25.7
8	188	21.8	20.8	19	17.5	26	25	24	21.8	30.3	38	21.6	24.4

8	189	21.4	20.2	18.7	16.5	25.5	24.3	23.1	20.5	29.9	37	21.1	23.4
8	190	21.2	19.9	19	15.9	25	23.6	22.2	19.2	29.1	35.2	20.8	22.7
8	191	21.4	20.2	20.5	16.5	25.5	24.3	23.1	20.5	28.2	33.1	20.3	21.6
8	192	22.2	21.3	22.1	18.6	26.5	25.7	24.9	23.1	27.3	31	19.9	20.7
9	193	23.3	22.7	23.9	20.9	27.5	26.7	25.9	24.1	26.4	28.6	19.6	18.6
9	194	24.3	24.1	26.6	23.5	29	28.8	28.6	28.1	25.7	27	19.4	18
9	195	25.8	26.2	28.4	27.5	30.5	30.9	31.3	32.1	25.1	25.5	19.1	17.4
9	196	26.8	27.6	30.2	30.2	32	33	34	36.1	24.5	24.3	18.9	17
9	197	27.8	29	31.7	32.9	33	34.4	35.8	38.7	18.7	10.8	18.7	16.6
9	198	28.6	30.1	32	35	33.5	35.1	36.7	40.1	20	13.8	18.5	16.1
9	199	28.8	30.4	31.1	35.5	33.5	35.1	36.7	40.1	20.1	14	18.4	15.9
9	200	28.3	29.7	29.5	34.2	33	34.4	35.8	38.7	20	13.9	18.3	15.6
9	201	27.4	28.5	28.1	31.8	32.5	33.7	34.9	37.4	20	13.8	18.5	16
9	202	26.6	27.3	27	29.7	31.5	32.3	33.1	34.8	19.9	13.7	18.5	16.1
9	203	26	26.5	25.9	28.1	31	31.6	32.2	33.4	20	13.9	18.6	16.3
9	204	25.4	25.7	25	26.5	30.5	30.9	31.3	32.1	20.8	15.7	18.9	17
9	205	24.9	25	24.1	25.1	30	30.2	30.4	30.8	22.4	19.5	20.2	20
9	206	24.4	24.3	23.2	23.8	29.5	29.5	29.5	29.4	24.5	24.2	21.8	23.6
9	207	23.9	23.6	22.3	22.5	29	28.8	28.6	28.1	26.5	28.7	22.3	24.8
9	208	23.4	22.9	22	21.2	28.5	28.1	27.7	26.8	28.2	32.7	23	26.4
9	209	23.2	22.6	21.2	20.6	28	27.4	26.8	25.4	29.4	35.6	23.3	27.1
9	210	22.8	22	20.5	19.6	27.5	26.7	25.9	24.1	30.2	37.3	23.6	27.6
9	211	22.4	21.5	20.2	18.5	27	26	25	22.8	30.4	37.8	23.2	26.8
9	212	22.2	21.2	19.4	18	26.5	25.3	24.1	21.5	30	36.9	22.7	25.8
9	213	21.8	20.6	19.3	16.9	26.5	25.3	24.1	21.5	29.3	35.3	22.1	24.2
9	214	21.7	20.5	19.4	16.6	26	24.6	23.2	20.1	28.4	33.3	21.3	22.5
9	215	21.8	20.6	20.7	16.9	26	24.6	23.2	20.1	27.6	31.4	20.6	20.9
9	216	22.5	21.6	22.4	18.8	27	26	25	22.8	26.3	28.3	20	19.4
10	217	23.8	23.1	24.9	20.8	28.5	27.8	27.1	25.5	23.5	22.6	19.4	20.7
10	218	25.2	25	26.9	24.5	30	29.9	29.8	29.5	21.4	17.8	18.9	19.6
10	219	26.3	26.6	29.6	27.4	31	31.3	31.6	32.1	21.5	17.9	18.5	18.7
10	220	27.8	28.7	31.4	31.4	32.5	33.4	34.3	36.1	21.3	17.5	18.3	18.1
10	221	28.8	30.1	32.3	34.1	34	35.5	37	40.1	21	16.8	18	17.4
10	222	29.3	30.8	33	35.4	34.5	36.2	37.9	41.5	20.7	16.2	17.7	16.7
10	223	29.7	31.3	32.1	36.5	34.5	36.2	37.9	41.5	20.4	15.5	17.4	16
10	224	29.2	30.6	30.6	35.2	34	35.5	37	40.1	20.2	14.9	17.2	15.5
10	225	28.4	29.5	29	33	33.5	34.8	36.1	38.8	19.8	14.2	17.1	15.4
10	226	27.5	28.3	27.9	30.6	32.5	33.4	34.3	36.1	19.7	13.9	17	15.2
10	227	26.9	27.4	26.9	29	32	32.7	33.4	34.8	19.7	13.8	17	15.2
10	228	26.3	26.6	26	27.4	31.5	32	32.5	33.5	20.2	15	17.4	16
10	229	25.8	25.9	25.1	26.1	30.5	30.6	30.7	30.8	21.9	18.9	17.5	16.4
10	230	25.3	25.2	24.3	24.8	30	29.9	29.8	29.5	23.8	23.2	17.9	17.1
10	231	24.9	24.6	23.4	23.7	30	29.9	29.8	29.5	25.7	27.6	18.6	18.8
10	232	24.4	23.9	22.5	22.4	29	28.5	28	26.8	27.5	31.7	19.5	20.9
10	233	23.9	23.2	22.2	21.1	28.5	27.8	27.1	25.5	28.8	34.8	20.1	22.1
10	234	23.7	22.9	21.5	20.5	28	27.1	26.2	24.2	29.7	36.9	19.9	21.9
10	235	23.3	22.4	20.7	19.5	28	27.1	26.2	24.2	30.3	38.2	19.8	21.6
10	236	22.9	21.8	20.4	18.4	27.5	26.4	25.3	22.8	30.1	37.9	19.6	21.1
10	237	22.7	21.5	19.8	17.9	27	25.7	24.4	21.5	29.6	36.6	19.4	20.7
10	238	22.4	21.1	20.6	17.1	26.5	25	23.5	20.2	28.8	34.7	19.1	20
10	239	22.8	21.7	21.5	18.1	26.5	25	23.5	20.2	27.9	32.8	18.7	19.1
10	240	23.3	22.4	22.6	19.5	27.5	26.4	25.3	22.8	27	30.7	18.4	18.3
11	241	24.3	23.5	25	20.8	28.5	28	27.5	26.4	26.2	26.2	18	13.6
11	242	25.6	25.3	27.3	24.3	30	30.1	30.2	30.4	25.4	24.5	17.8	13.1
11	243	26.9	27.1	29.8	27.7	31.5	32.2	32.9	34.4	24.8	23.1	17.5	12.5
11	244	28.3	29.1	30.9	31.4	32.5	33.6	34.7	37.1	24.3	21.9	17.4	12.1
11	245	28.9	29.9	32.2	33	33.5	35	36.5	39.7	23.8	20.7	17.2	11.6
11	246	29.6	30.9	32.9	34.9	34	35.7	37.4	41.1	23.3	19.7	17	11.2
11	247	30	31.4	32.2	36	34	35.7	37.4	41.1	22.9	18.8	16.9	10.9
11	248	29.6	30.9	30.9	34.9	33.5	35	36.5	39.7	22.5	17.8	16.7	10.6
11	249	28.9	29.9	30	33	32.5	33.6	34.7	37.1	22.2	17	16.8	10.8
11	250	28.4	29.2	28.9	31.7	32	32.9	33.8	35.7	21.9	16.5	17	11.2
11	251	27.8	28.4	28	30.1	31.5	32.2	32.9	34.4	22	16.6	17.4	12.2
11	252	27.3	27.7	27.1	28.8	30.5	30.8	31.1	31.7	22.6	18.1	18.3	14.2
11	253	26.8	27	26.2	27.4	30	30.1	30.2	30.4	24.4	22.2	21	20.4

11	254	26.3	26.3	25.3	26.1	29	28.7	28.4	27.8	26.5	26.9	23.7	26.6
11	255	25.8	25.6	24.4	24.8	28.5	28	27.5	26.4	28.4	31.5	25.9	31.8
11	256	25.3	24.9	23.7	23.5	28	27.3	26.6	25.1	30	35	27.9	36.4
11	257	24.9	24.3	23.3	22.4	27.5	26.6	25.7	23.8	30.8	36.9	28.7	38.2
11	258	24.7	24	22.8	21.9	27.5	26.6	25.7	23.8	30.1	35.3	28.8	38.4
11	259	24.4	23.6	22.4	21.1	27	25.9	24.8	22.4	29.6	34.2	28.7	38.2
11	260	24.2	23.3	21.9	20.5	26.5	25.2	23.9	21.1	29.9	34.8	27.4	35.1
11	261	23.9	22.9	21.7	19.7	26.5	25.2	23.9	21.1	29.6	34.2	25.8	31.5
11	262	23.8	22.8	21.7	19.5	26	24.5	23	19.8	29.1	32.9	24.2	27.9
11	263	23.8	22.8	22.8	19.5	26.5	25.2	23.9	21.1	28.5	31.5	22.9	24.7
11	264	24.4	23.6	23.8	21.1	27	25.9	24.8	22.4	27.7	29.8	21.6	21.7
12	265	25.3	24.6	26.1	22.2	28	27.7	27.3	26.6	27.1	29	20.5	17.3
12	266	26.6	26.4	28.3	25.6	29.5	29.8	30	30.6	26.4	27.4	19.5	15
12	267	27.8	28.1	30.6	28.8	30.5	31.2	31.8	33.2	25.9	26.2	18.5	12.8
12	268	29.1	29.9	32.8	32.3	32	33.3	34.5	37.2	25.4	25.1	17.7	11
12	269	30.3	31.6	33.7	35.5	33	34.7	36.3	39.9	25	24.1	17	9.2
12	270	30.8	32.3	34.4	36.8	33	34.7	36.3	39.9	24.5	23	16.4	7.8
12	271	31.2	32.8	33.3	37.9	33	34.7	36.3	39.9	24.1	22	15.7	6.3
12	272	30.6	32	32.1	36.3	32.5	34	35.4	38.6	23.7	21.1	15.2	5.2
12	273	29.9	31	31	34.4	31.5	32.6	33.6	35.9	23.3	20.4	15.2	5.2
12	274	29.3	30.2	29.7	32.8	31	31.9	32.7	34.6	23.1	19.8	16.2	7.4
12	275	28.6	29.2	28.8	31	30	30.5	30.9	31.9	22.9	19.5	18.3	12.3
12	276	28.1	28.5	27.6	29.6	29.5	29.8	30	30.6	23.3	20.3	20.8	18
12	277	27.4	27.5	26.7	27.8	29	29.1	29.1	29.2	24.1	22	23.7	24.8
12	278	26.9	26.8	25.8	26.4	28.5	28.4	28.2	27.9	25.3	24.9	26.7	31.5
12	279	26.4	26.1	24.9	25.1	28	27.7	27.3	26.6	26.7	28.2	29.3	37.6
12	280	25.9	25.4	24.2	23.8	27.5	27	26.4	25.3	28.6	32.4	31.2	41.9
12	281	25.5	24.8	23.6	22.7	27	26.3	25.5	23.9	30	35.8	31.5	42.6
12	282	25.2	24.4	23.1	21.9	26.5	25.6	24.6	22.6	30.8	37.6	31.4	42.3
12	283	24.9	24	22.4	21.1	26	24.9	23.7	21.3	31.3	38.7	30.6	40.4
12	284	24.5	23.4	22.2	20.1	25.5	24.2	22.8	19.9	31.5	39.2	29.5	38
12	285	24.4	23.3	22	19.8	25	23.5	21.9	18.6	22.1	17.5	28.3	35.3
12	286	24.3	23.2	22	19.5	25	23.5	21.9	18.6	23.3	20.3	27.1	32.4
12	287	24.3	23.2	23.1	19.5	25	23.5	21.9	18.6	23.2	20.2	25.8	29.5
12	288	24.9	24	24.7	21.1	26	24.9	23.7	21.3	23	19.7	24.8	27.2
13	289	25.8	25.3	26.5	23.5	27.5	26.7	25.9	24.1	22.6	22	23.9	26.3
13	290	26.8	26.7	29	26.2	29	28.8	28.6	28.1	22.2	21	23.2	24.7
13	291	28.2	28.6	31	29.9	30.5	30.9	31.3	32.1	21.8	20.1	22.6	23.3
13	292	29.3	30.2	32.4	32.8	32	33	34	36	21.4	19.2	22.1	22.1
13	293	30.1	31.3	33.5	35	32.5	33.7	34.9	37.4	21.1	18.5	21.6	21
13	294	30.7	32.1	33.7	36.6	33	34.4	35.8	38.7	20.7	17.7	21.1	19.7
13	295	30.8	32.3	32.8	36.8	33.5	35.1	36.7	40	20.4	16.9	20.6	18.5
13	296	30.3	31.6	31.4	35.5	33	34.4	35.8	38.7	20.1	16.1	20.2	17.7
13	297	29.5	30.4	30.5	33.4	32.5	33.7	34.9	37.4	19.8	15.4	20.1	17.5
13	298	29	29.7	29.2	32	32	33	34	36	19.6	15	20.4	18.1
13	299	28.3	28.8	28.3	30.2	31.5	32.3	33.1	34.7	19.5	14.8	20.6	18.7
13	300	27.8	28.1	27.4	28.8	30.5	30.9	31.3	32.1	20.1	16.1	21.1	19.8
13	301	27.3	27.4	26.5	27.5	30	30.2	30.4	30.7	21.7	20	22.7	23.4
13	302	26.8	26.7	25.8	26.2	29.5	29.5	29.5	29.4	23.5	24.1	24.1	26.6
13	303	26.4	26.1	24.9	25.1	28.5	28.1	27.7	26.7	25.1	27.7	23	24.1
13	304	25.9	25.4	24.7	23.8	28	27.4	26.8	25.4	26.2	30.2	22.4	22.7
13	305	25.8	25.3	24	23.5	28	27.4	26.8	25.4	27.2	32.4	23.6	25.6
13	306	25.4	24.7	23.3	22.5	27.5	26.7	25.9	24.1	27.6	33.4	24.8	28.3
13	307	25	24.1	23.1	21.4	27	26	25	22.7	27	32.1	24.7	28
13	308	24.9	24	22.7	21.1	27	26	25	22.7	26.8	31.6	23.6	25.6
13	309	24.7	23.7	22.2	20.6	26.5	25.3	24.1	21.4	26.3	30.4	22.4	22.7
13	310	24.4	23.3	22.2	19.8	26.5	25.3	24.1	21.4	25.5	28.6	21.4	20.5
13	311	24.4	23.3	22.5	19.8	26.5	25.3	24.1	21.4	24.6	26.5	20.6	18.7
13	312	24.6	23.6	24.7	20.3	27	26	25	22.7	23.8	24.6	19.9	17.1
14	313	24.8	24.8	25.8	24.6	27.5	27.2	26.9	26.2	23	21.6	19.4	20
14	314	25.4	25.6	26.7	26.2	28	27.9	27.8	27.5	22.4	20.1	18.8	18.7
14	315	25.9	26.3	27.2	27.5	28.5	28.6	28.7	28.8	21.8	18.8	18.3	17.6
14	316	26.2	26.7	27.6	28.3	29	29.3	29.6	30.2	21.3	17.7	17.9	16.6
14	317	26.4	27	26.9	28.9	29.5	30	30.5	31.5	20.8	16.5	17.5	15.9
14	318	26	26.4	27.1	27.8	30.5	31.4	32.3	34.2	20.4	15.5	17.2	15

14	319	26.1	26.6	27.8	28.1	30.5	31.4	32.3	34.2	19.9	14.5	16.9	14.4
14	320	26.5	27.1	27.4	29.1	30.5	31.4	32.3	34.2	19.6	13.7	16.8	14.1
14	321	26.3	26.9	27.1	28.6	30	30.7	31.4	32.8	19.3	13	16.7	14
14	322	26.1	26.6	26.5	28.1	30	30.7	31.4	32.8	19.2	12.8	16.9	14.4
14	323	25.8	26.2	25.8	27.3	29.5	30	30.5	31.5	19.1	12.6	17.2	15.1
14	324	25.4	25.6	25.6	26.2	29	29.3	29.6	30.2	19.6	13.7	17.7	16.3
14	325	25.3	25.5	24.9	26	28.5	28.6	28.7	28.8	21.2	17.4	18	17
14	326	24.9	24.9	24.7	24.9	28	27.9	27.8	27.5	23	21.7	18.9	18.9
14	327	24.8	24.8	24	24.6	28	27.9	27.8	27.5	25	26.2	19.5	20.3
14	328	24.4	24.2	23.8	23.6	27.5	27.2	26.9	26.2	26.9	30.7	20.1	21.8
14	329	24.3	24.1	23.3	23.3	27.5	27.2	26.9	26.2	28.6	34.5	20.8	23.4
14	330	24	23.6	23.1	22.5	27	26.5	26	24.9	29.8	37.3	21.7	25.5
14	331	23.9	23.5	22.2	22.2	27	26.5	26	24.9	30.4	38.7	22.1	26.4
14	332	23.4	22.8	21.8	20.9	26.5	25.8	25.1	23.5	30.5	38.8	21.7	25.4
14	333	23.2	22.5	21.1	20.4	26.5	25.8	25.1	23.5	30	37.8	20.8	23.4
14	334	22.8	22	21.1	19.3	26.5	25.8	25.1	23.5	29.3	36.1	19.9	21.3
14	335	22.8	22	21.7	19.3	26.5	25.8	25.1	23.5	28.4	33.9	19	19.3
14	336	23.1	22.4	23.3	20.1	27	26.5	26	24.9	27.4	31.7	18.2	17.3
15	337	23.9	23.6	24.9	22.7	27.5	26.7	26	24.3	26.5	27.4	17.4	12.3
15	338	24.8	24.9	26.7	25.1	28	27.4	26.9	25.6	25.7	25.6	16.5	10.5
15	339	25.8	26.3	29.3	27.8	29	28.8	28.7	28.3	25	23.9	15.9	8.9
15	340	27.2	28.2	30.9	31.5	30.5	30.9	31.4	32.3	24.4	22.5	15.2	7.3
15	341	28.1	29.5	31.2	33.9	31.5	32.3	33.2	34.9	23.8	21.2	14.5	5.7
15	342	28.3	29.8	30.9	34.4	32.5	33.7	35	37.6	23.3	20.1	13.9	4.3
15	343	28.1	29.5	29.6	33.9	32.5	33.7	35	37.6	22.9	19.1	13.3	3
15	344	27.4	28.5	28.9	32	33	34.4	35.9	38.9	22.5	18.1	12.9	2
15	345	27	28	27.6	31	32.5	33.7	35	37.6	22.1	17.3	12.9	2.1
15	346	26.3	27	26.7	29.1	32	33	34.1	36.3	21.8	16.7	13.7	4
15	347	25.8	26.3	26	27.8	31.5	32.3	33.2	34.9	21.8	16.5	15.9	8.9
15	348	25.4	25.7	24.9	26.7	31	31.6	32.3	33.6	22.3	17.8	18.5	15.1
15	349	24.8	24.9	24	25.1	30	30.2	30.5	30.9	23	19.5	21.6	22.1
15	350	24.3	24.2	23.1	23.8	30	30.2	30.5	30.9	24.4	22.5	24.9	29.8
15	351	23.8	23.5	22.2	22.4	29.5	29.5	29.6	29.6	26	26.2	28.2	37.2
15	352	23.3	22.8	21.7	21.1	28.5	28.1	27.8	26.9	27.9	30.6	30.4	42.4
15	353	23	22.4	21.2	20.3	28	27.4	26.9	25.6	29.3	34	31.7	45.3
15	354	22.7	21.9	20.4	19.5	27.5	26.7	26	24.3	30.4	36.4	32	46
15	355	22.3	21.4	19.9	18.5	27.5	26.7	26	24.3	30.8	37.3	31.3	44.4
15	356	22	21	19.4	17.7	27	26	25.1	23	30.2	36	29.4	40.1
15	357	21.7	20.5	18.8	16.9	27	26	25.1	23	29.9	35.2	27.4	35.5
15	358	21.4	20.1	18.8	16.1	27	26	25.1	23	29.2	33.6	25.6	31.2
15	359	21.4	20.1	19.9	16.1	26.5	25.3	24.2	21.6	28.4	31.8	23.9	27.3
15	360	22	21	21	17.7	26.5	25.3	24.2	21.6	27.5	29.8	22.4	23.9
16	361	22.9	22	23.4	19	26.5	26.5	26.5	26.6	26.7	26.7	21	17.2
16	362	24.2	23.8	25.3	22.5	26.5	26.5	26.5	26.6	26	25.1	19.9	14.6
16	363	25.3	25.3	28	25.4	27	27.2	27.4	27.9	25.3	23.5	18.8	12.1
16	364	26.8	27.4	30.7	29.4	27	27.2	27.4	27.9	24.7	22.1	17.9	10.1
16	365	28.3	29.5	31.6	33.4	27.5	27.9	28.3	29.2	24.1	20.9	17.1	8.2
16	366	28.8	30.2	32.4	34.7	27.5	27.9	28.3	29.2	23.6	19.7	16.4	6.7
16	367	29.2	30.8	31.8	35.8	27.5	27.9	28.3	29.2	23.2	18.7	15.7	5.1
16	368	28.9	30.4	30	35	27.5	27.9	28.3	29.2	22.8	17.7	15.2	3.9
16	369	27.9	29	28.6	32.3	27	27.2	27.4	27.9	22.3	16.8	15.2	3.9
16	370	27.1	27.8	27.7	30.2	27	27.2	27.4	27.9	22.1	16.2	16.2	6.2
16	371	26.6	27.1	26.4	28.8	27	27.2	27.4	27.9	21.9	15.8	18.5	11.4
16	372	25.9	26.2	25.5	27	27	27.2	27.4	27.9	22.3	16.7	21.7	18.7
16	373	25.4	25.5	24.6	25.6	26.5	26.5	26.5	26.6	23.9	20.4	25.2	26.9
16	374	24.9	24.8	23.7	24.3	26.5	26.5	26.5	26.6	25.7	24.6	28.7	35
16	375	24.4	24.1	23.4	23	26	25.8	25.6	25.2	27.6	28.9	31.7	41.7
16	376	24.2	23.8	22.6	22.5	26	25.8	25.6	25.2	29.3	32.8	33.5	46
16	377	23.8	23.2	21.9	21.4	26	25.8	25.6	25.2	30.7	36	33.7	46.5
16	378	23.4	22.7	21.7	20.3	25.5	25.1	24.7	23.9	31.6	38.1	32.8	44.3
16	379	23.3	22.5	21	20.1	25.5	25.1	24.7	23.9	32.1	39.2	32.6	43.9
16	380	22.9	22	21	19	25.5	25.1	24.7	23.9	32.1	39.3	31.5	41.3
16	381	22.9	22	20.7	19	25.5	25.1	24.7	23.9	31.7	38.3	29.9	37.7
16	382	22.7	21.7	20.8	18.5	25.5	25.1	24.7	23.9	30.9	36.4	28.4	34.3
16	383	22.8	21.8	22.1	18.7	25.5	25.1	24.7	23.9	30	34.3	27	31

16	384	23.5	22.8	23.3	20.6	26	25.8	25.6	25.2	28.7	31.4	25.6	27.7
17	385	24.6	24	24.9	21.9	27	26.7	26.4	25.8	26.6	28.2	24.4	22.9
17	386	25.5	25.2	27.1	24.3	27	26.7	26.4	25.8	25.5	25.5	23.2	20.3
17	387	26.7	26.9	29.6	27.5	27	26.7	26.4	25.8	24.6	23.5	22.3	18.1
17	388	28.1	28.9	30.9	31.2	27.5	27.4	27.3	27.1	23.9	21.8	21.6	16.5
17	389	28.8	29.8	32.3	33.1	28	28.1	28.2	28.4	23.2	20.3	20.9	14.9
17	390	29.6	31	33.2	35.2	29	29.5	30	31.1	22.6	18.9	20.4	13.6
17	391	30.1	31.7	33	36.5	29	29.5	30	31.1	22.1	17.8	20	12.8
17	392	30	31.5	31.6	36.3	29.5	30.2	30.9	32.4	21.7	16.8	19.7	12.1
17	393	29.2	30.4	30.3	34.1	29.5	30.2	30.9	32.4	21.4	16.1	19.7	12.2
17	394	28.5	29.4	29.2	32.3	29	29.5	30	31.1	21.3	15.9	20.2	13.4
17	395	27.9	28.6	27.3	30.7	29	29.5	30	31.1	21.2	15.8	21.2	15.5
17	396	26.8	27	26.2	27.8	28.5	28.8	29.1	29.7	21.6	16.6	23.1	19.8
17	397	26.2	26.2	25.5	26.2	28.5	28.8	29.1	29.7	23.1	20	25.1	24.4
17	398	25.8	25.6	24.7	25.1	28.5	28.8	29.1	29.7	24.7	23.8	26.8	28.5
17	399	25.4	25.1	23.8	24	28	28.1	28.2	28.4	26.5	27.8	29.1	33.6
17	400	24.9	24.4	23.7	22.7	28	28.1	28.2	28.4	28.1	31.6	30.8	37.7
17	401	24.8	24.2	22.9	22.4	27.5	27.4	27.3	27.1	29.4	34.5	32	40.5
17	402	24.4	23.7	22.8	21.4	27	26.7	26.4	25.8	29.7	35.3	32.9	42.4
17	403	24.3	23.5	22	21.1	27	26.7	26.4	25.8	30	35.9	32.4	41.4
17	404	23.9	23	21.9	20	26.5	26	25.5	24.4	30.1	36.1	31.5	39.4
17	405	23.8	22.8	21.1	19.8	26	25.3	24.6	23.1	29.8	35.4	30.5	36.9
17	406	23.4	22.3	21.1	18.7	26	25.3	24.6	23.1	29	33.7	29.4	34.5
17	407	23.4	22.3	21	18.7	26	25.3	24.6	23.1	28.3	32	28.3	31.9
17	408	23.3	22.1	19.4	18.4	27	26.7	26.4	25.8	27.6	30.3	27.2	29.5
18	409	21.9	20.6	21	16.7	28	27.8	27.6	27.2	19.8	14.1	26.3	23.7
18	410	22.8	21.9	23.5	19.1	29.5	29.9	30.3	31.2	21.1	17	25.6	21.9
18	411	24.2	23.9	26.4	22.8	30.5	31.3	32.1	33.9	21	16.9	24.9	20.3
18	412	25.8	26.1	28.9	27	31.5	32.7	33.9	36.5	21	16.9	24.2	18.9
18	413	27.2	28.1	30.4	30.8	32	33.4	34.8	37.8	20.8	16.5	23.6	17.5
18	414	28	29.2	30.9	32.9	32	33.4	34.8	37.8	20.7	16.2	23.1	16.2
18	415	28.3	29.6	30.7	33.7	31.5	32.7	33.9	36.5	20.6	15.9	22.4	14.7
18	416	28.2	29.5	30.2	33.4	31.5	32.7	33.9	36.5	20.4	15.4	21.9	13.6
18	417	27.9	29	29.5	32.6	31	32	33	35.2	20.2	15	21.7	13
18	418	27.5	28.5	28.6	31.6	30	30.6	31.2	32.5	20.1	14.7	22.3	14.5
18	419	27	27.8	24.2	30.2	29.5	29.9	30.3	31.2	20.1	14.7	23.9	18
18	420	24.6	24.4	24.2	23.9	28.5	28.5	28.5	28.5	20.6	16	26.2	23.5
18	421	24.6	24.4	24.4	23.9	28	27.8	27.6	27.2	22.3	19.9	29.1	30
18	422	24.7	24.6	23.9	24.1	27.5	27.1	26.7	25.9	24.4	24.6	31.7	36
18	423	24.4	24.1	23.7	23.3	27.5	27.1	26.7	25.9	26.5	29.5	33.2	39.6
18	424	24.3	24	23.2	23.1	27	26.4	25.8	24.5	28	33.1	35	43.6
18	425	24	23.6	23	22.3	26.5	25.7	24.9	23.2	29.4	36.1	35.8	45.5
18	426	23.9	23.4	22.8	22	26.5	25.7	24.9	23.2	30.1	37.9	36	45.9
18	427	23.8	23.3	22.8	21.7	26.5	25.7	24.9	23.2	30.5	38.8	35.6	45
18	428	23.8	23.3	22.3	21.7	26	25	24	21.9	30	37.6	34.5	42.5
18	429	23.5	22.9	22.1	20.9	26	25	24	21.9	29.2	35.7	33	39
18	430	23.4	22.7	22.4	20.7	25.5	24.3	23.1	20.6	28	33.1	31.5	35.6
18	431	23.6	23	22.8	21.2	25.5	24.3	23.1	20.6	27.1	30.9	30.1	32.3
18	432	23.8	23.3	24.2	21.7	25.5	24.3	23.1	20.6	26.3	29	28.7	29.3
19	433	24.1	24.2	24.9	24.3	26	25.7	25.5	24.9	25.5	27.7	27.5	26.8
19	434	24.5	24.7	25.7	25.4	26	25.7	25.5	24.9	24.8	26	26.4	24.2
19	435	24.9	25.3	25.8	26.5	26.5	26.4	26.4	26.2	24.3	24.8	25.3	21.8
19	436	25	25.4	26	26.7	27	27.1	27.3	27.6	21.8	19.2	24.4	19.8
19	437	25.1	25.6	26.6	27	28	28.5	29.1	30.2	21.8	19.2	23.6	17.8
19	438	25.4	26	26.4	27.8	28.5	29.2	30	31.5	21.7	19	22.8	16
19	439	25.3	25.8	26	27.5	29	29.9	30.9	32.9	21.6	18.7	22.1	14.3
19	440	25.1	25.6	26	27	29	29.9	30.9	32.9	21.5	18.3	21.5	13
19	441	25.1	25.6	25.5	27	29	29.9	30.9	32.9	21.3	17.9	21.2	12.3
19	442	24.8	25.1	25.1	26.2	28.5	29.2	30	31.5	21.2	17.8	21.7	13.6
19	443	24.6	24.9	24.8	25.7	28	28.5	29.1	30.2	21.3	17.9	23.2	17
19	444	24.4	24.6	24	25.1	27.5	27.8	28.2	28.9	21.4	18.3	25.6	22.4
19	445	24	24	23.7	24.1	27	27.1	27.3	27.6	22	19.6	28.3	28.7
19	446	23.8	23.7	23.1	23.5	27	27.1	27.3	27.6	22.7	21.2	31.2	35.3
19	447	23.5	23.3	23	22.7	26.5	26.4	26.4	26.2	23.7	23.5	32.6	38.6
19	448	23.4	23.2	22.8	22.5	26	25.7	25.5	24.9	25.2	26.8	33.7	41

19	449	23.3	23	22.1	22.2	26	25.7	25.5	24.9	26	28.8	34.4	42.7
19	450	22.9	22.5	22.1	21.1	25.5	25	24.6	23.6	27.2	31.5	35	44
19	451	22.9	22.5	21.9	21.1	25	24.3	23.7	22.2	27.1	31.3	35	44.2
19	452	22.8	22.3	21.9	20.9	25	24.3	23.7	22.2	27.4	32	34.2	42.2
19	453	22.8	22.3	21.2	20.9	24.5	23.6	22.8	20.9	27	31.1	32.7	38.9
19	454	22.4	21.8	21.2	19.8	24.5	23.6	22.8	20.9	26.4	29.6	31.3	35.6
19	455	22.4	21.8	21.5	19.8	24.5	23.6	22.8	20.9	25.8	28.3	30	32.6
19	456	22.6	22.1	22.8	20.3	25.5	25	24.6	23.6	25.2	27	28.7	29.7
20	457	22.8	22.8	22.8	22.8	26.5	25.7	24.9	23.1	24.6	26.5	27.6	28.5
20	458	22.8	22.8	23.1	22.8	28	27.8	27.6	27.1	24.1	25.3	26.5	26
20	459	23	23.1	23.3	23.3	29.5	29.9	30.3	31.1	23.6	24.2	25.6	23.9
20	460	23.1	23.2	24	23.5	30.5	31.3	32.1	33.8	23.2	23.2	24.7	21.9
20	461	23.5	23.8	24	24.6	31.5	32.7	33.9	36.4	22.8	22.3	24	20.3
20	462	23.5	23.8	24.6	24.6	32.5	34.1	35.7	39.1	22.3	21.2	23.3	18.7
20	463	23.8	24.2	24.9	25.4	32.5	34.1	35.7	39.1	21.9	20.3	22.6	17.1
20	464	24	24.5	25.1	25.9	32.5	34.1	35.7	39.1	21.5	19.5	22.1	15.8
20	465	24.1	24.6	25.1	26.2	31.5	32.7	33.9	36.4	21.1	18.5	21.9	15.4
20	466	24.1	24.6	24.9	26.2	31	32	33	35.1	20.9	17.9	22.4	16.5
20	467	24	24.5	24.6	25.9	30	30.6	31.2	32.4	20.7	17.7	23.7	19.7
20	468	23.8	24.2	23.7	25.4	29.5	29.9	30.3	31.1	20.8	17.8	25.6	24
20	469	23.3	23.5	23	24.1	29	29.2	29.4	29.8	21	18.2	27.1	27.5
20	470	22.9	22.9	22.8	23	28.5	28.5	28.5	28.4	21.4	19.1	29.6	33.1
20	471	22.8	22.8	22.1	22.8	28	27.8	27.6	27.1	22.6	21.8	30.7	35.6
20	472	22.4	22.2	21.9	21.7	27.5	27.1	26.7	25.8	23.4	23.9	31.1	36.7
20	473	22.3	22.1	21.2	21.4	27	26.4	25.8	24.4	23.7	24.5	31.2	36.8
20	474	21.9	21.5	21.2	20.4	26.5	25.7	24.9	23.1	24.5	26.2	30.7	35.8
20	475	21.9	21.5	20.8	20.4	26	25	24	21.8	25.6	28.8	30.6	35.5
20	476	21.7	21.2	20.3	19.8	25.5	24.3	23.1	20.5	25.9	29.6	30.4	34.9
20	477	21.4	20.8	20.3	19	25.5	24.3	23.1	20.5	25.9	29.4	29.7	33.3
20	478	21.4	20.8	20.3	19	25	23.6	22.2	19.1	25.3	28	28.8	31.3
20	479	21.4	20.8	21.3	19	25	23.6	22.2	19.1	24.6	26.6	27.9	29.2
20	480	22	21.7	20.7	20.6	26	25	24	21.8	23.9	25	26.9	27
21	481	22.9	21.8	22.3	18.3	27	26	25	22.7	23.3	23	26.1	28.1
21	482	23.8	23	24.5	20.7	28.5	28.1	27.7	26.7	22.8	21.7	25.4	26.5
21	483	25	24.7	26.6	23.9	30	30.2	30.4	30.7	22.3	20.7	24.8	25
21	484	26.2	26.4	28.4	27.1	31.5	32.3	33.1	34.7	21.9	19.8	24.2	23.7
21	485	27.2	27.8	30	29.7	32.5	33.7	34.9	37.4	21.5	18.9	23.7	22.6
21	486	28.1	29.1	30.6	32.1	33	34.4	35.8	38.7	21.2	18.1	23.4	21.9
21	487	28.4	29.5	30.4	32.9	33	34.4	35.8	38.7	20.9	17.5	23.1	21.2
21	488	28.3	29.3	29.5	32.6	32.5	33.7	34.9	37.4	20.6	16.8	22.9	20.7
21	489	27.8	28.6	28.6	31.3	32	33	34	36	20.3	16.1	22.5	19.7
21	490	27.3	27.9	27.7	30	31.5	32.3	33.1	34.7	20.1	15.7	22.1	18.9
21	491	26.8	27.2	27	28.6	31	31.6	32.2	33.4	20	15.5	22.1	18.9
21	492	26.4	26.7	26.4	27.6	30.5	30.9	31.3	32.1	20.5	16.5	22.7	20.2
21	493	26.1	26.3	25.9	26.8	30	30.2	30.4	30.7	22	20	23.9	23
21	494	25.8	25.8	25.4	26	29.5	29.5	29.5	29.4	23.5	23.5	26	27.8
21	495	25.5	25.4	25	25.2	29	28.8	28.6	28.1	25.4	27.9	27.4	30.9
21	496	25.3	25.1	24.5	24.7	28.5	28.1	27.7	26.7	27.4	32.3	28	32.4
21	497	25	24.7	24.1	23.9	28	27.4	26.8	25.4	28.3	34.6	28.1	32.5
21	498	24.8	24.4	23.6	23.3	28	27.4	26.8	25.4	27.7	33.2	27.1	30.4
21	499	24.5	24	23.4	22.5	27.5	26.7	25.9	24.1	26.8	31	26.9	29.9
21	500	24.4	23.9	23	22.3	27.5	26.7	25.9	24.1	26.5	30.4	26.1	27.9
21	501	24.2	23.6	22.7	21.7	27	26	25	22.7	26.2	29.7	25.1	25.8
21	502	24	23.3	22.8	21.2	27	26	25	22.7	25.7	28.6	24.1	23.5
21	503	24.1	23.5	23.7	21.5	27	26	25	22.7	25.2	27.3	23	20.9
21	504	24.6	24.2	23.8	22.8	27.5	26.7	25.9	24.1	24.6	25.9	22.1	18.7
22	505	25.4	24.6	25.8	22.1	28.5	28.2	27.9	27.3	23.9	22.7	21.1	16.8
22	506	26.5	26.1	27.9	25	29.5	29.6	29.7	30	23.4	21.5	20.4	15.2
22	507	27.7	27.8	30.1	28.2	30.5	31	31.5	32.6	22.9	20.5	19.7	13.6
22	508	28.9	29.5	32.1	31.4	31	31.7	32.4	34	22.5	19.4	19.2	12.3
22	509	30	31	32.8	34.3	31	31.7	32.4	34	22.1	18.4	18.6	11
22	510	30.4	31.6	33	35.4	31	31.7	32.4	34	21.7	17.6	18	9.7
22	511	30.5	31.7	32.6	35.6	31	31.7	32.4	34	21.3	16.7	17.5	8.4
22	512	30.3	31.5	31	35.1	31	31.7	32.4	34	20.9	15.9	17	7.4
22	513	29.4	30.2	30.3	32.7	30.5	31	31.5	32.6	20.6	15.2	17.1	7.6

22	514	29	29.6	29.4	31.6	30.5	31	31.5	32.6	20.4	14.7	18.1	9.9
22	515	28.5	28.9	28.7	30.3	30	30.3	30.6	31.3	20.3	14.4	20.1	14.5
22	516	28.1	28.4	28.1	29.2	30	30.3	30.6	31.3	20.8	15.5	22.4	19.8
22	517	27.8	28	27.2	28.5	29.5	29.6	29.7	30	22.5	19.4	25	25.7
22	518	27.3	27.3	26.5	27.1	29	28.9	28.8	28.7	24.5	24	27.6	31.7
22	519	26.9	26.7	26	26.1	29	28.9	28.8	28.7	26.5	28.7	30.1	37.3
22	520	26.6	26.3	25.4	25.3	28.5	28.2	27.9	27.3	28.3	32.7	32	41.9
22	521	26.3	25.9	24.7	24.5	28.5	28.2	27.9	27.3	29.7	36	33.5	45.2
22	522	25.9	25.3	24.5	23.4	28	27.5	27	26	30.6	38.2	33.8	45.9
22	523	25.8	25.2	23.8	23.1	28	27.5	27	26	31	39.1	33.4	44.9
22	524	25.4	24.6	23.6	22.1	27.5	26.8	26.1	24.7	31	38.9	31.8	41.4
22	525	25.3	24.5	23.1	21.8	27.5	26.8	26.1	24.7	30.4	37.6	30	37.2
22	526	25	24	23.6	21	27	26.1	25.2	23.3	29.4	35.4	28.3	33.3
22	527	25.3	24.5	24	21.8	27	26.1	25.2	23.3	27.2	30.3	26.7	29.6
22	528	25.5	24.7	26.7	22.3	27	26.1	25.2	23.3	24.4	23.8	25.3	26.4
23	529	26.4	26.5	28.7	27	27	26.8	26.7	26.3	23.2	21.5	24.1	24.6
23	530	27.5	28.1	29.8	29.9	27.5	27.5	27.6	27.6	22.7	20.4	23.1	22.3
23	531	28.1	28.9	30.7	31.5	28	28.2	28.5	28.9	22.3	19.5	22.1	20
23	532	28.6	29.6	29.6	32.9	28.5	28.9	29.4	30.3	22	18.6	21.4	18.4
23	533	28	28.8	28.3	31.3	29	29.6	30.3	31.6	21.6	17.8	20.9	17.1
23	534	27.3	27.8	28.3	29.4	29.5	30.3	31.2	32.9	21.2	17	20.4	15.9
23	535	27.3	27.8	28.3	29.4	30	31	32.1	34.3	20.9	16.1	19.9	14.8
23	536	27.3	27.8	28	29.4	30	31	32.1	34.3	20.5	15.2	19.4	13.6
23	537	27.1	27.5	27.4	28.9	29.5	30.3	31.2	32.9	20.1	14.4	19.4	13.8
23	538	26.8	27.1	27.1	28.1	29.5	30.3	31.2	32.9	19.9	13.9	20	15.1
23	539	26.6	26.8	26.5	27.5	29	29.6	30.3	31.6	19.8	13.6	21.1	17.7
23	540	26.3	26.4	26	26.7	28.5	28.9	29.4	30.3	20.3	14.9	22.7	21.4
23	541	26	26	25.6	25.9	28	28.2	28.5	28.9	22.1	19	24.2	24.9
23	542	25.8	25.7	24.9	25.4	27.5	27.5	27.6	27.6	24.1	23.5	24.6	25.7
23	543	25.4	25.1	24.7	24.3	27	26.8	26.7	26.3	26.1	28.1	24.9	26.3
23	544	25.3	25	24	24.1	27	26.8	26.7	26.3	27.8	32	25.9	28.6
23	545	24.9	24.4	24	23	26.5	26.1	25.8	24.9	29	34.9	28.1	33.6
23	546	24.9	24.4	23.8	23	26	25.4	24.9	23.6	29.9	36.9	29.1	35.9
23	547	24.8	24.3	23.1	22.8	25.5	24.7	24	22.3	30.3	37.8	29.6	37.2
23	548	24.4	23.7	22.9	21.7	25.5	24.7	24	22.3	30.2	37.6	29.1	35.9
23	549	24.3	23.6	22.2	21.4	25	24	23.1	21	29.7	36.4	27.3	31.9
23	550	23.9	23	22.2	20.4	24.5	23.3	22.2	19.6	28.9	34.6	25.6	28
23	551	23.9	23	22.2	20.4	24.5	23.3	22.2	19.6	27.9	32.3	24.4	25.1
23	552	23.9	23	24.6	20.4	25.5	24.7	24	22.3	27.1	30.4	23.4	22.8
24	553	24.3	24.5	25.1	24.9	26.5	26	25.4	24.3	26.3	27	22.4	18.3
24	554	24.6	24.9	26.4	25.7	27.5	27.4	27.2	26.9	25.5	25.1	21.4	16.1
24	555	25.3	25.9	26.6	27.6	28.5	28.8	29	29.6	24.8	23.5	20.6	14.1
24	556	25.4	26	26.8	27.8	29.5	30.2	30.8	32.2	24.1	21.9	19.8	12.4
24	557	25.5	26.1	26.8	28.1	30	30.9	31.7	33.6	23.6	20.7	19.1	10.8
24	558	25.5	26.1	26.6	28.1	30.5	31.6	32.6	34.9	23.1	19.7	18.5	9.4
24	559	25.4	26	26.4	27.8	31	32.3	33.5	36.2	22.7	18.6	17.9	7.9
24	560	25.3	25.9	26.4	27.6	31	32.3	33.5	36.2	22.2	17.6	17.4	6.8
24	561	25.3	25.9	26	27.6	30.5	31.6	32.6	34.9	21.8	16.5	17.3	6.6
24	562	25.1	25.6	25.5	27.1	30	30.9	31.7	33.6	21.5	15.9	18.2	8.8
24	563	24.8	25.2	24.8	26.3	29.5	30.2	30.8	32.2	21.3	15.6	20	12.8
24	564	24.4	24.6	24.6	25.2	29	29.5	29.9	30.9	21.7	16.5	22.7	19.2
24	565	24.3	24.5	23.9	24.9	28.5	28.8	29	29.6	23.3	20	26	26.6
24	566	23.9	23.9	23	23.9	28	28.1	28.1	28.2	24.9	23.7	29.3	34.3
24	567	23.4	23.2	23	22.5	27.5	27.4	27.2	26.9	26.5	27.5	31.9	40.1
24	568	23.4	23.2	22.1	22.5	27	26.7	26.3	25.6	28	30.9	34	45.2
24	569	22.9	22.5	22.1	21.2	26.5	26	25.4	24.3	29.2	33.7	35.2	47.8
24	570	22.9	22.5	21.2	21.2	26	25.3	24.5	22.9	30.2	35.9	35.2	47.9
24	571	22.4	21.8	21.2	19.9	26	25.3	24.5	22.9	30.6	37	34.5	46.2
24	572	22.4	21.8	20.5	19.9	25.5	24.6	23.6	21.6	30.7	37.1	33.2	43.3
24	573	22	21.2	20.3	18.8	25	23.9	22.7	20.3	30.3	36.2	31.5	39.4
24	574	21.9	21.1	20.3	18.5	24.5	23.2	21.8	18.9	29.6	34.5	30	35.8
24	575	21.9	21.1	20.3	18.5	25	23.9	22.7	20.3	28.7	32.6	28.4	32.3
24	576	21.9	21.1	21.6	18.5	25.5	24.6	23.6	21.6	27.9	30.5	27.2	29.4
25	577	22.3	21.9	22.5	20.8	26.5	25.7	25	23.3	27.1	27.5	26.1	24.9
25	578	22.8	22.6	23.6	22.2	27.5	27.1	26.8	25.9	26.4	25.9	25.1	22.6

25	579	23.4	23.5	25.2	23.8	28.5	28.5	28.6	28.6	25.8	24.6	24.2	20.8
25	580	24.3	24.7	26.1	26.2	29.5	29.9	30.4	31.3	25.3	23.4	23.5	19
25	581	24.8	25.4	27	27.5	30.5	31.3	32.2	33.9	24.8	22.3	22.9	17.7
25	582	25.3	26.1	27.9	28.8	31.5	32.7	34	36.6	24.4	21.4	22.5	16.7
25	583	25.8	26.8	27.7	30.1	31	32	33.1	35.3	24	20.3	22.1	15.7
25	584	25.7	26.7	27	29.9	31	32	33.1	35.3	23.4	19	21.7	15
25	585	25.3	26.1	26.1	28.8	30.5	31.3	32.2	33.9	22.9	17.9	21.7	14.9
25	586	24.8	25.4	25.4	27.5	30	30.6	31.3	32.6	22.5	16.9	22.2	16
25	587	24.4	24.9	25	26.4	29.5	29.9	30.4	31.3	22.2	16.3	23.6	19.2
25	588	24.2	24.6	24.3	25.9	29.5	29.9	30.4	31.3	22.5	16.9	25.2	22.9
25	589	23.8	24	23.4	24.8	29	29.2	29.5	29.9	23.9	20.3	26.8	26.7
25	590	23.3	23.3	22.7	23.5	28.5	28.5	28.6	28.6	25.7	24.3	28.7	31
25	591	22.9	22.8	22	22.4	28	27.8	27.7	27.3	27.4	28.2	30.5	35.1
25	592	22.5	22.2	21.6	21.4	27.5	27.1	26.8	25.9	28.8	31.6	32	38.7
25	593	22.3	21.9	20.9	20.8	27.5	27.1	26.8	25.9	30.2	34.8	32.7	40.3
25	594	21.9	21.4	20.2	19.8	27	26.4	25.9	24.6	31.3	37.2	32.5	39.7
25	595	21.5	20.8	19.8	18.7	27	26.4	25.9	24.6	31.7	38.1	32.3	39.3
25	596	21.3	20.5	19.3	18.2	26.5	25.7	25	23.3	31.4	37.5	31.9	38.4
25	597	21	20.1	19.1	17.4	26.5	25.7	25	23.3	31.3	37.1	31	36.4
25	598	20.9	20	19.1	17.1	26.5	25.7	25	23.3	30.5	35.5	30	34
25	599	20.9	20	19.3	17.1	26.5	25.7	25	23.3	29.6	33.3	29	31.7
25	600	21	20.1	20.8	17.4	26.5	25.7	25	23.3	28.7	31.2	27.9	29.2
26	601	21.6	21.2	21.5	19.9	26.5	25	23.5	20.3	27.8	29.3	27	27.5
26	602	22	21.8	23.1	21	28	27.1	26.2	24.3	27	27.5	26.2	25.8
26	603	22.9	23	24	23.4	29	28.5	28	26.9	26.5	26.2	25.6	24.2
26	604	23.4	23.7	25.3	24.7	30.5	30.6	30.7	30.9	26	25.1	25	22.9
26	605	24.1	24.7	25.7	26.6	31.5	32	32.5	33.6	25.6	24.2	24.5	21.8
26	606	24.3	25	25.8	27.1	32	32.7	33.4	34.9	25.3	23.5	24.1	20.8
26	607	24.4	25.1	26	27.4	32	32.7	33.4	34.9	25	22.8	23.7	20.1
26	608	24.5	25.3	25.7	27.6	32.5	33.4	34.3	36.2	24.5	21.8	23.4	19.3
26	609	24.3	25	25.7	27.1	32	32.7	33.4	34.9	24.1	20.9	23.5	19.5
26	610	24.3	25	24.8	27.1	32	32.7	33.4	34.9	23.9	20.4	23.9	20.5
26	611	23.8	24.3	24	25.8	32	32.7	33.4	34.9	24	20.5	25	22.9
26	612	23.4	23.7	23.9	24.7	31.5	32	32.5	33.6	24.2	21	26	25.2
26	613	23.3	23.6	23	24.5	31	31.3	31.6	32.2	24.6	21.9	26.9	27.4
26	614	22.8	22.9	22.2	23.1	31	31.3	31.6	32.2	25	22.8	29	32.1
26	615	22.4	22.3	21.9	22.1	30.5	30.6	30.7	30.9	25.6	24.3	30.6	35.7
26	616	22.2	22	21.3	21.5	30.5	30.6	30.7	30.9	26.9	27.2	31.2	37.1
26	617	21.9	21.6	20.6	20.7	30	29.9	29.8	29.6	28.1	30	30.7	36
26	618	21.5	21.1	20.3	19.7	29.5	29.2	28.9	28.3	29.3	32.8	29.7	33.7
26	619	21.3	20.8	19.5	19.1	29.5	29.2	28.9	28.3	30	34.4	28.6	31.3
26	620	20.9	20.2	19.4	18.1	29	28.5	28	26.9	30.2	34.8	28.1	30.1
26	621	20.8	20.1	18.8	17.8	29	28.5	28	26.9	30	34.4	27.4	28.5
26	622	20.5	19.7	19.4	17	29	28.5	28	26.9	29.5	33.3	26.6	26.7
26	623	20.8	20.1	19.9	17.8	28.5	27.8	27.1	25.6	28.9	31.8	25.9	25.1
26	624	21.1	20.5	20.3	18.6	29	28.5	28	26.9	28.2	30.1	25.3	23.6
27	625	21.6	20.9	22.2	18.8	29.5	28.7	28	26.3	27.5	26.9	24.6	22.9
27	626	22.7	22.5	23.9	21.8	30.5	30.1	29.8	29	26.9	25.6	24.2	21.9
27	627	23.6	23.7	25.1	24.2	32	32.2	32.5	33	26.3	24.3	23.8	21
27	628	24.3	24.7	27.1	26	33.5	34.3	35.2	37	25.9	23.3	23.5	20.3
27	629	25.4	26.3	28.2	28.9	34.5	35.7	37	39.6	25.5	22.4	23.2	19.7
27	630	26	27.1	28.7	30.5	35	36.4	37.9	40.9	25.2	21.7	23.1	19.3
27	631	26.3	27.5	28.5	31.3	35	36.4	37.9	40.9	25	21.4	22.6	18.3
27	632	26.2	27.4	26.9	31.1	34.5	35.7	37	39.6	24.9	21	22.4	17.8
27	633	25.3	26.1	26	28.7	34	35	36.1	38.3	24.7	20.5	22.4	17.8
27	634	24.8	25.4	25.3	27.3	33.5	34.3	35.2	37	24.5	20	22.6	18.3
27	635	24.4	24.9	24.4	26.3	33	33.6	34.3	35.6	24.2	19.5	23.1	19.4
27	636	23.9	24.2	24.2	25	33	33.6	34.3	35.6	24.4	19.9	23.8	21
27	637	23.8	24	23.3	24.7	32.5	32.9	33.4	34.3	25.6	22.7	25.7	25.4
27	638	23.3	23.3	22.6	23.4	32	32.2	32.5	33	27.3	26.5	28.3	31.3
27	639	22.9	22.8	21.7	22.3	31.5	31.5	31.6	31.6	28.9	30.2	29.7	34.6
27	640	22.4	22.1	21.3	21	28.5	27.3	26.2	23.7	30.2	33.1	30.7	36.8
27	641	22.2	21.8	20.8	20.4	29.5	28.7	28	26.3	31.4	35.9	32	40
27	642	21.9	21.4	20.4	19.6	29.5	28.7	28	26.3	32	37.3	31.2	37.9
27	643	21.7	21.1	19.9	19.1	29	28	27.1	25	32.3	38.1	30.1	35.5

27	644	21.4	20.7	19.5	18.3	29	28	27.1	25	32.4	38.2	29.6	34.2
27	645	21.2	20.4	19	17.8	29	28	27.1	25	32	37.4	28.4	31.6
27	646	20.9	20	19	17	28.5	27.3	26.2	23.7	31.3	35.8	27.1	28.6
27	647	20.9	20	19.7	17	28.5	27.3	26.2	23.7	30.6	34.1	26	26
27	648	21.3	20.5	20.5	18	28.5	27.3	26.2	23.7	29.8	32.2	25	23.7
28	649	22	21.3	22.2	19	29	28.7	28.4	27.7	29.1	33	24.1	23
28	650	22.9	22.5	24.3	21.4	29	28.7	28.4	27.7	28.6	31.8	23.2	21
28	651	24.1	24.2	26.5	24.6	29.5	29.4	29.3	29	24.2	21.8	22.4	19.2
28	652	25.3	25.9	27.7	27.8	30	30.1	30.2	30.3	23.9	20.9	21.9	17.9
28	653	26	26.9	28.6	29.6	30.5	30.8	31.1	31.6	23.4	19.9	21.4	16.7
28	654	26.5	27.6	29.2	31	31.5	32.2	32.9	34.3	23	19	20.9	15.6
28	655	26.8	28	28.5	31.8	32	32.9	33.8	35.6	22.6	18.1	20.2	14.1
28	656	26.4	27.4	27.4	30.7	32	32.9	33.8	35.6	22.3	17.3	19.9	13.3
28	657	25.8	26.6	26.5	29.1	32	32.9	33.8	35.6	22	16.6	19.9	13.3
28	658	25.3	25.9	25.6	27.8	32	32.9	33.8	35.6	21.8	16.2	20.2	14
28	659	24.8	25.2	24.9	26.4	32	32.9	33.8	35.6	21.8	16.2	20.4	14.6
28	660	24.4	24.6	24	25.4	31.5	32.2	32.9	34.3	22.2	17.1	21.6	17.3
28	661	23.9	23.9	23.8	24	31	31.5	32	33	23.2	19.5	23.2	20.9
28	662	23.8	23.8	23.1	23.8	30.5	30.8	31.1	31.6	25	23.6	25.3	25.8
28	663	23.4	23.2	22.3	22.7	30	30.1	30.2	30.3	27	28.1	28.6	33.2
28	664	23	22.7	22.2	21.6	29.5	29.4	29.3	29	28.6	31.8	31.2	39.4
28	665	22.9	22.5	21.3	21.4	29	28.7	28.4	27.7	29.9	34.9	32.5	42.4
28	666	22.4	21.8	21.3	20	28.5	28	27.5	26.3	30.7	36.7	32.6	42.6
28	667	22.4	21.8	20.4	20	28.5	28	27.5	26.3	31.2	37.7	31.8	40.7
28	668	21.9	21.1	20.4	18.7	28	27.3	26.6	25	31.1	37.7	30.2	37.1
28	669	21.9	21.1	20.2	18.7	27.5	26.6	25.7	23.7	30.6	36.5	28.7	33.7
28	670	21.8	21	20.2	18.5	27	25.9	24.8	22.3	29.7	34.3	27.4	30.5
28	671	21.8	21	20.7	18.5	27	25.9	24.8	22.3	28.7	32.1	26.1	27.5
28	672	22.1	21.4	20.4	19.3	28	27.3	26.6	25	27.9	30.1	24.9	24.8
29	673	22.5	21.4	22	18.1	29	28.3	27.6	26.1	27.1	31.1	23.7	20.4
29	674	23.4	22.7	24	20.5	30	29.7	29.4	28.8	26.4	29.4	22.7	18.1
29	675	24.5	24.2	25.8	23.4	31	31.1	31.2	31.4	21.1	17.2	21.9	16.1
29	676	25.5	25.6	27.6	26.1	32	32.5	33	34.1	21	17.1	21.1	14.2
29	677	26.5	27	28.5	28.7	33.5	34.6	35.7	38.1	21	17	20.2	12.3
29	678	27	27.7	28.7	30.1	34	35.3	36.6	39.4	20.9	16.8	19.6	10.7
29	679	27.1	27.9	28.3	30.3	34	35.3	36.6	39.4	20.8	16.6	18.9	9.2
29	680	26.9	27.6	28.1	29.8	33.5	34.6	35.7	38.1	20.7	16.3	18.3	7.9
29	681	26.8	27.5	27.6	29.5	33	33.9	34.8	36.7	20.6	16.2	18	7.3
29	682	26.5	27	27.2	28.7	32.5	33.2	33.9	35.4	20.6	16.2	18.7	8.9
29	683	26.3	26.8	26.3	28.2	32	32.5	33	34.1	20.7	16.2	20.5	12.9
29	684	25.8	26.1	26	26.9	31.5	31.8	32.1	32.7	21.1	17.3	23.2	19.2
29	685	25.6	25.8	25.4	26.3	31	31.1	31.2	31.4	22.6	20.7	26.6	26.8
29	686	25.3	25.4	25.1	25.5	31	31.1	31.2	31.4	24.2	24.5	29.8	34.3
29	687	25.1	25.1	24.5	25	30.5	30.4	30.3	30.1	25.9	28.3	32.5	40.6
29	688	24.8	24.7	24.5	24.2	30	29.7	29.4	28.8	27.4	31.6	34.4	44.9
29	689	24.8	24.7	24	24.2	29.5	29	28.5	27.4	28.4	34.1	35.5	47.4
29	690	24.5	24.2	23.8	23.4	29.5	29	28.5	27.4	29.2	35.8	35.9	48.4
29	691	24.4	24.1	23.6	23.1	29	28.3	27.6	26.1	29.4	36.4	35.5	47.5
29	692	24.3	24	23.3	22.9	28.5	27.6	26.7	24.8	28.7	34.6	34.3	44.7
29	693	24.1	23.7	23.1	22.4	28.5	27.6	26.7	24.8	27.7	32.3	32.5	40.6
29	694	24	23.5	22.9	22.1	28	26.9	25.8	23.4	26.5	29.8	30.8	36.6
29	695	23.9	23.4	23.3	21.8	28	26.9	25.8	23.4	24.5	25	29.2	32.9
29	696	24.1	23.7	23.2	22.4	28.5	27.6	26.7	24.8	23.9	23.7	27.9	29.9
30	697	24.5	23.8	24.6	21.7	29.5	28.7	27.8	26	23.3	22.7	26.7	25
30	698	25.3	24.9	26	23.8	30.5	30.1	29.6	28.7	22.8	21.6	25.6	22.4
30	699	26.1	26.1	27.7	26	31.5	31.5	31.4	31.3	22.4	20.5	24.5	19.9
30	700	27	27.3	29.5	28.3	33	33.6	34.1	35.3	22	19.7	23.5	17.6
30	701	28	28.7	30.4	31	34	35	35.9	38	21.7	19	22.6	15.6
30	702	28.5	29.4	30.9	32.3	34.5	35.7	36.8	39.3	21.5	18.4	21.8	13.8
30	703	28.8	29.8	30.7	33.1	34	35	35.9	38	21.2	17.9	21.1	12
30	704	28.7	29.7	30	32.9	34	35	35.9	38	21.1	17.6	20.4	10.5
30	705	28.3	29.1	29.1	31.8	33.5	34.3	35	36.6	20.9	17.1	20	9.6
30	706	27.8	28.4	28.6	30.5	33	33.6	34.1	35.3	20.7	16.7	20.4	10.5
30	707	27.5	28	27.8	29.7	33	33.6	34.1	35.3	20.5	16.2	21.8	13.8
30	708	27.1	27.5	27.3	28.6	32.5	32.9	33.2	34	20.8	16.9	24.3	19.5

30	709	26.8	27	26.4	27.8	32	32.2	32.3	32.7	22.1	20	27.4	26.6
30	710	26.3	26.3	25.7	26.5	31.5	31.5	31.4	31.3	23.5	23.1	30.4	33.6
30	711	25.9	25.8	25.1	25.4	31.5	31.5	31.4	31.3	24.6	25.7	33.2	39.9
30	712	25.6	25.4	24.6	24.6	31	30.8	30.5	30	25.9	28.6	35.2	44.5
30	713	25.3	24.9	23.9	23.8	30.5	30.1	29.6	28.7	27	31.1	36.4	47.4
30	714	24.9	24.4	23.7	22.8	30.5	30.1	29.6	28.7	27.7	32.7	36.9	48.5
30	715	24.8	24.2	23	22.5	30	29.4	28.7	27.3	27.9	33.2	36.7	48.1
30	716	24.4	23.7	22.8	21.4	30	29.4	28.7	27.3	27.8	33	35.7	45.8
30	717	24.3	23.5	22.1	21.2	29.5	28.7	27.8	26	27.3	31.9	34.1	42.1
30	718	23.9	23	22.4	20.1	29.5	28.7	27.8	26	26.7	30.6	32.5	38.5
30	719	24.1	23.3	23.3	20.6	29.5	28.7	27.8	26	26	28.9	31	34.9
30	720	24.6	24	24.1	22	30	29.4	28.7	27.3	25.2	27.1	29.6	31.6
31	721	25.2	24.6	25.9	22.8	30.5	30.1	29.8	28.9	24.3	24.4	28.2	27.5
31	722	26.2	26	27.7	25.5	31.5	31.5	31.6	31.6	23.6	22.7	27.1	24.9
31	723	27.2	27.4	29.8	28.2	32	32.2	32.5	32.9	23	21.4	26.1	22.6
31	724	28.4	29.1	31.3	31.4	33	33.6	34.3	35.6	22.5	20.1	25.2	20.6
31	725	29.2	30.2	31.8	33.5	33	33.6	34.3	35.6	22	18.9	24.4	18.8
31	726	29.5	30.7	32.7	34.3	33	33.6	34.3	35.6	21.5	17.8	23.7	17
31	727	30	31.4	32.3	35.6	33	33.6	34.3	35.6	21.1	16.9	22.9	15.3
31	728	29.8	31.1	30.9	35.1	33	33.6	34.3	35.6	20.7	16	22.3	13.9
31	729	29	30	29.6	33	33	33.6	34.3	35.6	20.3	15.1	21.9	12.9
31	730	28.3	29	28.9	31.1	32.5	32.9	33.4	34.3	20.1	14.6	22.1	13.4
31	731	27.9	28.4	28	30	32	32.2	32.5	32.9	19.9	14.3	23.2	16
31	732	27.4	27.7	27.1	28.7	32	32.2	32.5	32.9	20.3	15.2	25.2	20.6
31	733	26.9	27	26.2	27.4	31.5	31.5	31.6	31.6	21.4	17.7	28	27
31	734	26.4	26.3	25.5	26	31.5	31.5	31.6	31.6	22.3	19.8	30.9	33.6
31	735	26	25.8	25.1	25	31	30.8	30.7	30.3	24	23.6	32.7	37.9
31	736	25.8	25.5	24.4	24.4	31	30.8	30.7	30.3	25.8	27.7	34.2	41.3
31	737	25.4	24.9	23.7	23.4	30.5	30.1	29.8	28.9	27.4	31.5	35.2	43.5
31	738	25	24.4	23.2	22.3	30.5	30.1	29.8	28.9	28.9	34.8	36.1	45.6
31	739	24.7	23.9	22.6	21.5	30.5	30.1	29.8	28.9	29.6	36.6	36.3	46.1
31	740	24.4	23.5	21.9	20.7	30	29.4	28.9	27.6	29.9	37.1	35.4	43.9
31	741	24	23	21.7	19.7	30	29.4	28.9	27.6	29.6	36.5	34.3	41.5
31	742	23.9	22.8	21.7	19.4	30	29.4	28.9	27.6	28.9	34.9	33	38.6
31	743	23.9	22.8	22.4	19.4	29.5	28.7	28	26.3	28	32.9	31.7	35.6
31	744	24.3	23.4	23.3	20.5	30	29.4	28.9	27.6	27.1	30.8	30.4	32.6
32	745	25	24.1	25.2	21.4	30.5	31.2	31.9	33.4	26.3	26.1	29.3	28.3
32	746	26.1	25.7	27.6	24.3	31	31.9	32.8	34.8	25.6	24.5	28.3	26
32	747	27.4	27.5	29.9	27.8	31	31.9	32.8	34.8	25	23.2	27.4	23.8
32	748	28.7	29.3	31.2	31.2	31	31.9	32.8	34.8	24.5	21.9	26.6	22
32	749	29.4	30.3	31.4	33.1	30.5	31.2	31.9	33.4	24	20.9	25.8	20.2
32	750	29.5	30.4	31.5	33.4	30.5	31.2	31.9	33.4	23.6	19.9	25.2	18.9
32	751	29.6	30.6	31.9	33.6	30	30.5	31	32.1	23.2	19	24.8	17.9
32	752	29.8	30.9	31	34.2	30.5	31.2	31.9	33.4	22.9	18.3	24.6	17.4
32	753	29.3	30.2	30.1	32.8	30.5	31.2	31.9	33.4	22.6	17.6	24.5	17.2
32	754	28.8	29.5	29.2	31.5	30	30.5	31	32.1	22.4	17.1	24.8	17.9
32	755	28.3	28.8	28.8	30.2	30	30.5	31	32.1	22.2	16.7	25.7	20.1
32	756	28.1	28.5	27.9	29.6	29.5	29.8	30.1	30.8	22.4	17.2	27.4	23.8
32	757	27.6	27.8	27.4	28.3	29	29.1	29.2	29.4	23.6	19.8	29.5	28.7
32	758	27.3	27.4	26.7	27.5	28.5	28.4	28.3	28.1	25.3	23.8	31.9	34.3
32	759	26.9	26.8	25.8	26.5	28	27.7	27.4	26.8	27.2	28.2	33.8	38.6
32	760	26.4	26.1	25.6	25.1	27.5	27	26.5	25.5	29	32.3	34.9	41.1
32	761	26.3	26	24.9	24.9	27.5	27	26.5	25.5	30.5	35.8	35.8	43.2
32	762	25.9	25.4	24.7	23.8	27	26.3	25.6	24.1	31.5	38	36.4	44.6
32	763	25.8	25.3	24	23.5	26.5	25.6	24.7	22.8	31.6	38.3	36.6	45.1
32	764	25.4	24.7	23.3	22.5	26.5	25.6	24.7	22.8	31.7	38.5	36	43.8
32	765	25	24.1	23.1	21.4	26	24.9	23.8	21.5	31.3	37.6	35	41.3
32	766	24.9	24	23.4	21.1	25.5	24.2	22.9	20.1	30.3	35.3	33.7	38.5
32	767	25.1	24.3	24.2	21.7	26	24.9	23.8	21.5	29.3	33	32.5	35.7
32	768	25.5	24.8	24.7	22.7	26.5	25.6	24.7	22.8	28.3	30.8	31.5	33.5
33	769	26.2	25.4	26.3	23	27.5	27	26.5	25.3	27.5	30.6	30.6	30.2
33	770	27.1	26.7	28.6	25.4	28.5	28.4	28.3	28	26.7	28.9	29.9	28.5
33	771	28.4	28.5	30.4	28.8	29.5	29.8	30.1	30.6	26	27.2	29.2	26.9
33	772	29.4	29.9	32.2	31.5	31	31.9	32.8	34.6	25.3	25.7	28.6	25.5
33	773	30.4	31.3	33.8	34.2	31.5	32.6	33.7	36	24.8	24.4	28.1	24.3

33	774	31.3	32.6	34.2	36.6	32	33.3	34.6	37.3	24.2	23.1	27.1	22
33	775	31.5	32.8	33.8	37.1	32	33.3	34.6	37.3	23.5	21.6	26.3	20.3
33	776	31.3	32.6	32.8	36.6	31.5	32.6	33.7	36	23	20.4	25.8	19.1
33	777	30.7	31.7	31.5	35	31	31.9	32.8	34.6	22.7	19.6	25.7	18.8
33	778	30	30.7	30.4	33.1	30.5	31.2	31.9	33.3	22.5	19.1	26	19.6
33	779	29.4	29.9	29.7	31.5	30	30.5	31	32	22.5	19.2	27.1	22.2
33	780	29	29.3	28.6	30.4	29.5	29.8	30.1	30.6	22.6	19.4	29	26.5
33	781	28.4	28.5	27.9	28.8	29	29.1	29.2	29.3	22.8	20	31.2	31.5
33	782	28	27.9	27.5	27.8	29	29.1	29.2	29.3	23.1	20.6	33.5	36.9
33	783	27.8	27.7	26.8	27.3	28.5	28.4	28.3	28	23.5	21.5	35.5	41.4
33	784	27.4	27.1	25.9	26.2	28	27.7	27.4	26.7	25.1	25.2	36.5	43.7
33	785	26.9	26.4	25.7	24.9	27.5	27	26.5	25.3	26.5	28.3	37	44.8
33	786	26.8	26.3	25	24.6	27	26.3	25.6	24	27.3	30.3	37.3	45.5
33	787	26.4	25.7	24.1	23.5	27	26.3	25.6	24	28.1	32	36.8	44.4
33	788	25.9	25	23.9	22.2	26.5	25.6	24.7	22.7	27.9	31.7	35.4	41.1
33	789	25.8	24.9	23.2	21.9	26	24.9	23.8	21.3	27.6	30.9	33.8	37.5
33	790	25.4	24.3	23.6	20.9	26	24.9	23.8	21.3	27	29.6	32.3	34.1
33	791	25.6	24.6	24.3	21.4	26	24.9	23.8	21.3	26.3	27.9	30.7	30.5
33	792	26	25.1	25.4	22.5	26.5	25.6	24.7	22.7	25.5	26	29.3	27.2
34	793	26.8	26.1	27.1	24	28	27	26	23.8	24.8	23.9	28.1	27
34	794	27.7	27.4	29.2	26.4	29	28.4	27.8	26.5	24.1	22.4	26.9	24.3
34	795	28.9	29.1	31.2	29.6	30.5	30.5	30.5	30.5	23.6	21.1	25.9	22
34	796	30	30.6	33.2	32.5	32	32.6	33.2	34.5	23.1	19.9	24.9	19.8
34	797	31.1	32.1	34.4	35.4	33	34	35	37.1	22.6	18.9	24.1	17.9
34	798	31.8	33.1	34.8	37.3	33.5	34.7	35.9	38.4	22.2	17.9	23.3	16.1
34	799	32	33.4	34.4	37.8	33.5	34.7	35.9	38.4	21.8	17	22.6	14.5
34	800	31.8	33.1	33	37.3	33.5	34.7	35.9	38.4	21.3	16	22.1	13.2
34	801	31	32	31.7	35.1	33	34	35	37.1	21	15.1	21.7	12.5
34	802	30.3	31	30.8	33.3	32.5	33.3	34.1	35.8	20.7	14.4	22.1	13.3
34	803	29.8	30.3	29.9	31.9	32	32.6	33.2	34.5	20.5	13.9	23.2	15.9
34	804	29.3	29.6	29	30.6	31.5	31.9	32.3	33.1	20.7	14.5	25.1	20.2
34	805	28.8	28.9	28.1	29.3	31	31.2	31.4	31.8	22.3	18.2	27.7	26.3
34	806	28.3	28.2	27.4	28	30.5	30.5	30.5	30.5	24.4	22.9	30.7	33
34	807	27.9	27.7	26.5	26.9	30	29.8	29.6	29.1	26.5	27.8	33.4	39.3
34	808	27.4	27	26.3	25.6	30	29.8	29.6	29.1	28.4	32.1	35.5	44.1
34	809	27.3	26.8	25.6	25.3	29.5	29.1	28.7	27.8	29.9	35.6	36.6	46.7
34	810	26.9	26.3	24.9	24.2	29	28.4	27.8	26.5	31	38.2	37.1	47.8
34	811	26.5	25.7	24.7	23.2	29	28.4	27.8	26.5	31.6	39.5	36.8	47.1
34	812	26.4	25.6	23.8	22.9	28.5	27.7	26.9	25.1	31.6	39.6	35.8	44.8
34	813	25.9	24.9	23.8	21.6	28.5	27.7	26.9	25.1	31	38.2	34.4	41.6
34	814	25.9	24.9	24.2	21.6	28	27	26	23.8	30.2	36.3	33	38.3
34	815	26.1	25.1	24.5	22.1	28	27	26	23.8	29.2	34.1	31.5	35
34	816	26.3	25.4	26	22.6	28.5	27.7	26.9	25.1	28.2	31.7	30.4	32.3
35	817	27	26.5	28	24.9	29	28.7	28.3	27.5	27.2	27.5	29.3	27.7
35	818	28.1	28	29.6	27.8	29.5	29.4	29.2	28.9	26.4	25.7	28.3	25.4
35	819	29	29.3	31.4	30.2	30	30.1	30.1	30.2	25.7	24	27.4	23.4
35	820	30	30.7	33	32.8	30.5	30.8	31	31.5	25.1	22.5	26.6	21.6
35	821	30.9	31.9	34.6	35.2	30.5	30.8	31	31.5	24.5	21.3	25.9	19.8
35	822	31.8	33.2	35.5	37.6	31	31.5	31.9	32.9	24	20.1	25.4	18.7
35	823	32.3	33.9	35.5	39	31	31.5	31.9	32.9	23.5	19	25	17.8
35	824	32.3	33.9	32.8	39	31.5	32.2	32.8	34.2	23.1	18.1	24.7	17.3
35	825	30.8	31.8	31	35	31.5	32.2	32.8	34.2	22.8	17.3	24.7	17.3
35	826	29.8	30.4	30.1	32.3	31.5	32.2	32.8	34.2	22.5	16.7	25.1	18
35	827	29.3	29.7	29.2	31	31	31.5	31.9	32.9	22.3	16.3	25.9	20
35	828	28.8	29	28.3	29.7	31	31.5	31.9	32.9	22.6	16.9	27.8	24.3
35	829	28.3	28.3	27.6	28.3	30.5	30.8	31	31.5	24	20	30.5	30.5
35	830	27.9	27.7	26.7	27.3	30	30.1	30.1	30.2	25.8	24.2	33	36.4
35	831	27.4	27	26.2	25.9	30	30.1	30.1	30.2	27.6	28.4	35	40.9
35	832	27.1	26.6	25.8	25.1	29.5	29.4	29.2	28.9	29.3	32.3	36.1	43.4
35	833	26.9	26.3	24.9	24.6	29.5	29.4	29.2	28.9	30.8	35.7	37	45.4
35	834	26.4	25.6	24.7	23.3	29	28.7	28.3	27.5	31.9	38.4	37.5	46.7
35	835	26.3	25.5	24	23	29	28.7	28.3	27.5	32.5	39.7	37.1	45.7
35	836	25.9	24.9	23.8	21.9	28.5	28	27.4	26.2	32.6	40	36.1	43.4
35	837	25.8	24.8	23.1	21.7	28.5	28	27.4	26.2	32.2	39	35	40.9
35	838	25.4	24.2	23.5	20.6	28	27.3	26.5	24.9	31.3	36.8	33.9	38.4

35	839	25.6	24.5	23.8	21.1	28	27.3	26.5	24.9	30.3	34.6	32.8	35.8
35	840	25.8	24.8	26.5	21.7	28.5	28	27.4	26.2	29.4	32.4	31.7	33.3
36	841	26.3	26.4	27.5	26.6	29.5	28.8	28	26.4	28.6	29.9	30.8	29
36	842	26.9	27.2	29.2	28.2	30.5	30.2	29.8	29	27.9	28.5	30.1	27.3
36	843	27.8	28.5	30.1	30.6	31.5	31.6	31.6	31.7	27.4	27.3	29.4	25.8
36	844	28.3	29.2	30.2	31.9	32	32.3	32.5	33	27	26.3	28.9	24.5
36	845	28.4	29.3	30.1	32.2	33	33.7	34.3	35.7	26.6	25.3	28.4	23.4
36	846	28.3	29.2	30.1	31.9	33.5	34.4	35.2	37	26.1	24.3	28	22.5
36	847	28.3	29.2	30.2	31.9	33.5	34.4	35.2	37	25.6	23.1	27.6	21.7
36	848	28.4	29.3	30.1	32.2	33.5	34.4	35.2	37	25.1	22	27.4	21.1
36	849	28.3	29.2	29.5	31.9	33.5	34.4	35.2	37	24.7	20.9	27.3	20.9
36	850	28	28.8	28.6	31.1	33	33.7	34.3	35.7	24.3	20.1	27.5	21.4
36	851	27.5	28.1	27.5	29.8	33	33.7	34.3	35.7	24.1	19.6	28.3	23.2
36	852	26.9	27.2	26.8	28.2	32.5	33	33.4	34.4	24.2	20	29.9	26.8
36	853	26.5	26.7	26.1	27.2	32	32.3	32.5	33	25.4	22.6	32.3	32.4
36	854	26.1	26.1	25.6	26.1	32	32.3	32.5	33	25.9	23.8	35	38.6
36	855	25.8	25.7	24.7	25.3	31.5	31.6	31.6	31.7	27	26.2	36.6	42.3
36	856	25.3	25	23.9	24	31	30.9	30.7	30.4	28.6	29.9	37.8	45.1
36	857	24.9	24.4	23	22.9	30.5	30.2	29.8	29	30.2	33.6	38.3	46.3
36	858	24.4	23.7	22.3	21.6	30.5	30.2	29.8	29	31.3	36.3	38.2	45.9
36	859	24	23.2	21.6	20.5	30	29.5	28.9	27.7	31.7	37.1	37.7	44.9
36	860	23.6	22.6	21.1	19.4	29.5	28.8	28	26.4	31.8	37.3	37	43.2
36	861	23.3	22.2	20.5	18.6	29.5	28.8	28	26.4	30.8	35	35.9	40.8
36	862	23	21.8	20.3	17.8	29	28.1	27.1	25.1	29.9	32.9	34.7	38
36	863	22.9	21.6	21.1	17.6	29	28.1	27.1	25.1	29	30.9	33.4	35
36	864	23.3	22.2	23.2	18.6	29.5	28.8	28	26.4	28.1	28.8	32.2	32.3
37	865	24	23.6	24.8	22.2	30	29.4	28.8	27.4	27.2	28	31.2	30.6
37	866	24.9	24.8	26.4	24.6	31	30.8	30.6	30.1	26.4	26.2	30.2	28.5
37	867	25.8	26.1	28.2	27	32	32.2	32.4	32.8	25.7	24.6	29.4	26.4
37	868	26.8	27.5	29.5	29.7	33	33.6	34.2	35.4	25.1	23.2	28.5	24.4
37	869	27.5	28.5	30	31.5	34	35	36	38.1	24.5	21.8	27.7	22.7
37	870	27.8	28.9	30.2	32.3	34.5	35.7	36.9	39.4	24	20.6	27.1	21.1
37	871	27.9	29	30.2	32.6	34	35	36	38.1	23.5	19.4	26.6	20
37	872	27.9	29	29.1	32.6	33.5	34.3	35.1	36.8	23	18.4	26.2	19.1
37	873	27.3	28.2	28.2	31	33	33.6	34.2	35.4	22.6	17.4	25.8	18.3
37	874	26.8	27.5	27.3	29.7	33	33.6	34.2	35.4	22.3	16.6	25.9	18.4
37	875	26.3	26.8	26.6	28.4	32.5	32.9	33.3	34.1	22	16.1	26.6	20.1
37	876	25.9	26.2	25.7	27.3	32.5	32.9	33.3	34.1	22.2	16.4	28.2	23.9
37	877	25.4	25.5	24.8	26	32	32.2	32.4	32.8	23.4	19.3	30.7	29.6
37	878	24.9	24.8	24.1	24.6	31.5	31.5	31.5	31.4	25.2	23.3	33.3	35.5
37	879	24.5	24.3	23.7	23.6	31	30.8	30.6	30.1	27	27.4	35.7	41.1
37	880	24.3	24	23	23	31	30.8	30.6	30.1	28.7	31.4	37.5	45.1
37	881	23.9	23.4	22.3	22	30.5	30.1	29.7	28.8	30.1	34.7	38.6	47.7
37	882	23.5	22.9	21.9	20.9	30	29.4	28.8	27.4	31.2	37.2	38.8	48.3
37	883	23.3	22.6	21	20.4	30	29.4	28.8	27.4	31.9	38.7	38.2	46.7
37	884	22.8	21.9	20.5	19	30	29.4	28.8	27.4	31.8	38.6	37.2	44.4
37	885	22.5	21.5	20.1	18.2	29.5	28.7	27.9	26.1	31.3	37.5	35.8	41.2
37	886	22.3	21.2	20.3	17.7	29.5	28.7	27.9	26.1	30.6	35.9	34.5	38.2
37	887	22.4	21.3	21	18	29.5	28.7	27.9	26.1	29.8	33.9	33.1	35.1
37	888	22.8	21.9	21.6	19	29.5	28.7	27.9	26.1	28.8	31.7	31.9	32.2
38	889	23.5	22.5	23.9	19.5	30	29.9	29.8	29.6	27.9	28.3	30.7	30.7
38	890	24.8	24.4	26.1	23	30.5	30.6	30.7	30.9	27.2	26.5	29.7	28.4
38	891	26	26	28.6	26.1	31	31.3	31.6	32.2	26.5	24.9	28.9	26.4
38	892	27.4	28	30.4	29.9	31	31.3	31.6	32.2	25.9	23.5	28.1	24.6
38	893	28.4	29.4	32	32.5	31	31.3	31.6	32.2	25.3	22.2	27.4	23.1
38	894	29.3	30.7	32.7	34.9	31.5	32	32.5	33.5	24.8	21.1	26.9	21.8
38	895	29.7	31.2	32	36	31.5	32	32.5	33.5	24.4	20.1	26.3	20.5
38	896	29.3	30.7	30.6	34.9	31	31.3	31.6	32.2	24	19.1	25.8	19.3
38	897	28.5	29.5	29.5	32.8	31	31.3	31.6	32.2	23.6	18.3	25.4	18.5
38	898	27.9	28.7	28.6	31.2	31	31.3	31.6	32.2	23.3	17.6	25.6	18.8
38	899	27.4	28	27.7	29.9	31	31.3	31.6	32.2	23.3	17.5	26.5	20.9
38	900	26.9	27.3	26.8	28.5	30.5	30.6	30.7	30.9	23.4	17.9	28.4	25.2
38	901	26.4	26.6	26.1	27.2	30.5	30.6	30.7	30.9	24.5	20.3	30.9	31.1
38	902	26	26	25.5	26.1	30	29.9	29.8	29.6	26.2	24.2	33.7	37.4
38	903	25.7	25.6	24.6	25.3	30	29.9	29.8	29.6	28	28.4	36	42.7

38	904	25.2	24.9	24.1	24	30	29.9	29.8	29.6	29.8	32.4	37.5	46.2
38	905	24.9	24.5	23.2	23.2	30	29.9	29.8	29.6	31.3	35.9	38.4	48.3
38	906	24.4	23.8	22.5	21.9	29.5	29.2	28.9	28.2	32.4	38.6	38.2	47.9
38	907	24	23.2	22.1	20.8	29.5	29.2	28.9	28.2	33.1	40	37	45
38	908	23.8	23	21.2	20.3	29.5	29.2	28.9	28.2	33.2	40.4	35	40.4
38	909	23.3	22.3	20.5	19	29.5	29.2	28.9	28.2	32.8	39.5	33.2	36.3
38	910	22.9	21.7	20.5	17.9	29	28.5	28	26.9	32.1	37.8	31.4	32.3
38	911	22.9	21.7	21.2	17.9	29	28.5	28	26.9	31.2	35.7	29.7	28.4
38	912	23.3	22.3	22.6	19	29	28.5	28	26.9	30.2	33.4	28.2	24.8
39	913	23.9	23.2	24.4	21.1	29.5	29.3	29.1	28.7	29.3	30.1	26.8	24.6
39	914	24.9	24.6	26.5	23.8	29.5	29.3	29.1	28.7	28.5	28.2	25.6	21.9
39	915	26.1	26.3	28.9	27	30	30	30	30.1	27.8	26.6	24.7	19.9
39	916	27.4	28.1	30.7	30.4	30	30	30	30.1	27.1	25	24.1	18.3
39	917	28.4	29.5	32.1	33.1	30.5	30.7	30.9	31.4	26.5	23.6	23.5	17
39	918	29.2	30.6	33	35.2	30.5	30.7	30.9	31.4	25.9	22.4	23	15.9
39	919	29.7	31.3	32.3	36.5	31	31.4	31.8	32.7	25.4	21.2	22.6	15
39	920	29.3	30.8	31.2	35.5	31	31.4	31.8	32.7	25	20.2	22.2	14.1
39	921	28.7	29.9	29.6	33.9	31	31.4	31.8	32.7	24.6	19.2	22.1	13.7
39	922	27.8	28.7	28.5	31.5	31	31.4	31.8	32.7	24.2	18.4	22.3	14.3
39	923	27.2	27.8	27.1	29.9	31	31.4	31.8	32.7	24.1	18.1	22.9	15.6
39	924	26.4	26.7	26.2	27.8	31	31.4	31.8	32.7	24.2	18.4	24.6	19.4
39	925	25.9	26	25.3	26.4	31	31.4	31.8	32.7	25.4	21.1	27.5	26.2
39	926	25.4	25.3	24.4	25.1	30.5	30.7	30.9	31.4	27.1	25	30.7	33.7
39	927	24.9	24.6	23.6	23.8	30	30	30	30.1	28.9	29.3	33.7	40.4
39	928	24.5	24.1	22.7	22.7	30	30	30	30.1	30.6	33.2	35.3	44.1
39	929	24	23.4	22	21.4	29.5	29.3	29.1	28.7	32.1	36.6	36.2	46.3
39	930	23.6	22.8	21.5	20.3	29.5	29.3	29.1	28.7	33.3	39.2	36.9	47.9
39	931	23.3	22.4	20.8	19.5	29	28.6	28.2	27.4	33.9	40.7	36.8	47.6
39	932	22.9	21.8	20.6	18.5	29	28.6	28.2	27.4	34	41	35.7	45
39	933	22.8	21.7	19.9	18.2	28.5	27.9	27.3	26.1	33.7	40.3	33.9	41
39	934	22.4	21.1	19.9	17.1	28.5	27.9	27.3	26.1	33	38.6	32.2	37.1
39	935	22.4	21.1	20.6	17.1	28.5	27.9	27.3	26.1	32	36.4	30.7	33.7
39	936	22.8	21.7	21.7	18.2	29	28.6	28.2	27.4	31	34	29.5	30.8
40	937	23.5	22.6	23.7	19.8	29.5	28.4	27.4	25.1	30	31.3	28.6	33.5
40	938	24.6	24.1	26.2	22.7	30.5	29.8	29.2	27.7	29.2	29.3	27.9	31.8
40	939	26	26.1	27.3	26.4	31.5	31.2	31	30.4	28.4	27.6	27.3	30.5
40	940	26.6	26.9	28	28	32.5	32.6	32.8	33.1	27.8	26.1	26.7	29.2
40	941	27	27.5	30.2	29.1	33.5	34	34.6	35.7	27.1	24.6	26.2	27.9
40	942	28.2	29.2	31.4	32.3	34	34.7	35.5	37	26.6	23.4	25.3	25.9
40	943	28.9	30.2	31.2	34.1	34	34.7	35.5	37	26	22.1	24.4	23.9
40	944	28.8	30	30.5	33.9	34	34.7	35.5	37	25.5	20.9	23.7	22.3
40	945	28.4	29.5	29.8	32.8	34	34.7	35.5	37	25	19.8	23.4	21.6
40	946	28	28.9	28.7	31.7	34	34.7	35.5	37	24.6	18.9	23.4	21.5
40	947	27.4	28.1	27.8	30.1	33.5	34	34.6	35.7	24.4	18.4	23.2	21.2
40	948	26.9	27.4	26.9	28.8	33	33.3	33.7	34.4	24.4	18.4	23.4	21.6
40	949	26.4	26.7	26	27.5	33	33.3	33.7	34.4	25.6	21.2	23.4	21.6
40	950	25.9	26	25.5	26.1	32.5	32.6	32.8	33.1	27.3	25.1	23.4	21.6
40	951	25.6	25.5	24.9	25.3	32.5	32.6	32.8	33.1	29.2	29.4	23.6	22
40	952	25.3	25.1	24.2	24.5	32	31.9	31.9	31.7	31.1	33.7	24.1	23
40	953	24.9	24.6	23.3	23.5	31.5	31.2	31	30.4	32.6	37.3	24.4	23.9
40	954	24.4	23.9	22.6	22.2	31.5	31.2	31	30.4	33.9	40.1	25.5	26.4
40	955	24	23.3	22.2	21.1	31.5	31.2	31	30.4	34.6	41.7	26.4	28.5
40	956	23.8	23	21.5	20.6	31	30.5	30.1	29.1	34.7	42.1	26.2	27.9
40	957	23.4	22.5	21.2	19.5	31	30.5	30.1	29.1	34	40.5	25.3	25.9
40	958	23.2	22.2	21.3	19	30.5	29.8	29.2	27.7	32.9	38	24.2	23.5
40	959	23.3	22.3	21.9	19.2	30.5	29.8	29.2	27.7	31.7	35.1	23.2	21.2
40	960	23.6	22.7	22.4	20	30.5	29.8	29.2	27.7	30.6	32.6	22.4	19.2
41	961	24.3	23.3	24	20.3	31	30.6	30.2	29.2	29.7	30.7	21.6	20.9
41	962	25.2	24.6	26	22.7	31.5	31.3	31.1	30.6	29	29.1	21	19.4
41	963	26.3	26.1	28.1	25.6	32.5	32.7	32.9	33.2	28.3	27.6	20.5	18.3
41	964	27.5	27.8	30.5	28.8	33	33.4	33.8	34.6	27.8	26.3	20	17.1
41	965	28.8	29.6	32.3	32.3	34	34.8	35.6	37.2	27.3	25.2	19.6	16.1
41	966	29.8	31	33	34.9	34.5	35.5	36.5	38.5	26.8	24.1	19.3	15.5
41	967	30.2	31.6	33.2	36	34.5	35.5	36.5	38.5	26.4	23.1	19.3	15.5
41	968	30.3	31.7	31.6	36.3	34.5	35.5	36.5	38.5	26	22.2	19.4	15.7

41	969	29.4	30.5	30.5	33.9	34	34.8	35.6	37.2	25.6	21.3	19.5	16
41	970	28.8	29.6	29.6	32.3	34	34.8	35.6	37.2	25.3	20.6	20.1	17.4
41	971	28.3	28.9	28.7	30.9	33.5	34.1	34.7	35.9	25.1	20.2	21.2	19.9
41	972	27.8	28.2	27.8	29.6	33	33.4	33.8	34.6	25.2	20.3	21.7	21.2
41	973	27.3	27.5	26.9	28.3	32.5	32.7	32.9	33.2	25.6	21.4	22.9	23.9
41	974	26.8	26.8	26.2	27	32	32	32	31.9	26.7	23.9	24	26.3
41	975	26.4	26.3	25.3	25.9	32	32	32	31.9	28.5	27.9	24.7	28.1
41	976	25.9	25.6	24.7	24.6	31.5	31.3	31.1	30.6	30	31.4	24.7	27.9
41	977	25.6	25.2	24.4	23.8	31	30.6	30.2	29.2	31.5	34.9	24.9	28.5
41	978	25.4	24.9	23.5	23.2	31	30.6	30.2	29.2	32.5	37.2	25.7	30.2
41	979	24.9	24.2	23.3	21.9	30.5	29.9	29.3	27.9	33.4	39.1	25.9	30.6
41	980	24.8	24	22.6	21.6	30	29.2	28.4	26.6	33.7	40	25	28.6
41	981	24.4	23.5	22.6	20.6	30	29.2	28.4	26.6	33.6	39.6	24.1	26.6
41	982	24.4	23.5	21.8	20.6	29.5	28.5	27.5	25.2	32.9	38.1	23.3	24.7
41	983	24	22.9	22.4	19.5	29.5	28.5	27.5	25.2	32	36	22.6	23
41	984	24.3	23.3	24.1	20.3	30	29.2	28.4	26.6	31.1	33.9	21.7	21.2
42	985	24.6	24.3	24.5	23.6	30.5	30.3	30.1	29.7	30.2	31.3	21	15
42	986	24.8	24.6	25.5	24.1	31	31	31	31	29.5	29.6	20.2	13.2
42	987	25.4	25.5	27.2	25.7	32	32.4	32.8	33.7	28.8	28	19.7	12.1
42	988	26.3	26.7	29.3	28.1	32.5	33.1	33.7	35	28.1	26.4	19.1	10.6
42	989	27.5	28.4	31.1	31.3	33	33.8	34.6	36.4	27.5	25	18.5	9.4
42	990	28.5	29.8	32.6	33.9	33.5	34.5	35.5	37.7	26.9	23.8	18	8.1
42	991	29.3	30.9	32.6	36.1	33.5	34.5	35.5	37.7	26.4	22.6	17.5	6.9
42	992	29.3	30.9	31.8	36.1	33	33.8	34.6	36.4	25.9	21.5	17.1	6
42	993	28.9	30.4	30.8	35	33	33.8	34.6	36.4	25.5	20.4	17	5.8
42	994	28.3	29.5	29.9	33.4	32.5	33.1	33.7	35	25.1	19.5	18	8.1
42	995	27.8	28.8	17.8	32.1	32	32.4	32.8	33.7	24.8	19	20.1	13
42	996	21.1	19.4	21.8	14.2	31.5	31.7	31.9	32.4	24.8	18.8	23.3	20.3
42	997	23.3	22.5	22.8	20.1	31.5	31.7	31.9	32.4	25.8	21.2	27	28.8
42	998	23.9	23.4	22.8	21.7	31	31	31	31	27.4	24.9	30.7	37.4
42	999	23.9	23.4	22.8	21.7	30.5	30.3	30.1	29.7	29.3	29.1	33.4	43.6
42	1000	23.9	23.4	22.8	21.7	30.5	30.3	30.1	29.7	31	33.2	35.7	48.7
42	1001	23.9	23.4	22.8	21.7	30	29.6	29.2	28.4	32.5	36.5	35.5	48.3
42	1002	23.9	23.4	22.8	21.7	29.5	28.9	28.3	27	33.5	39	35	47.3
42	1003	23.9	23.4	22.7	21.7	29.5	28.9	28.3	27	34.4	40.8	34.8	46.7
42	1004	23.8	23.2	22.1	21.4	29	28.2	27.4	25.7	34.7	41.7	33.5	43.7
42	1005	23.5	22.8	21.9	20.6	28.5	27.5	26.5	24.4	34.5	41.1	32	40.4
42	1006	23.4	22.7	21.9	20.4	28.5	27.5	26.5	24.4	33.6	39.2	30.6	37.1
42	1007	23.4	22.7	21	20.4	28.5	27.5	26.5	24.4	32.7	37.1	29.2	33.8
42	1008	22.9	22	20.5	19	28.5	27.5	26.5	24.4	31.8	35	27.9	30.8
43	1009	22.9	21.7	21.2	17.9	29	28.5	28	27	31	34.5	26.6	27
43	1010	23.3	22.3	22.5	19	30	29.9	29.8	29.7	30.2	32.8	25.5	24.4
43	1011	24	23.2	24.6	20.8	30.5	30.6	30.7	31	29.5	31.1	24.5	22.1
43	1012	25.2	24.9	26.6	24	31.5	32	32.5	33.6	28.6	29.2	23.7	20.3
43	1013	26.3	26.5	28.4	26.9	32	32.7	33.4	35	27.9	27.6	23.2	19
43	1014	27.3	27.9	29.7	29.6	32	32.7	33.4	35	27.3	26.2	22.7	18
43	1015	28	28.8	30.2	31.5	32	32.7	33.4	35	26.8	25	22.2	16.9
43	1016	28.3	29.3	29.5	32.3	31.5	32	32.5	33.6	26.3	23.8	21.9	16
43	1017	27.9	28.7	28.8	31.2	31.5	32	32.5	33.6	25.8	22.7	21.7	15.7
43	1018	27.5	28.1	28.4	30.1	31	31.3	31.6	32.3	25.4	21.8	22	16.3
43	1019	27.3	27.9	28.1	29.6	31	31.3	31.6	32.3	25.1	21.1	22.4	17.3
43	1020	27.1	27.6	27.5	29.1	30.5	30.6	30.7	31	25.2	21.2	23.2	19.2
43	1021	26.8	27.2	26.8	28.3	30	29.9	29.8	29.7	25.4	21.7	25.7	24.9
43	1022	26.4	26.6	26.6	27.2	30	29.9	29.8	29.7	25.6	22.3	29.1	32.7
43	1023	26.3	26.5	26.1	26.9	30	29.9	29.8	29.7	26.3	23.9	30.1	35
43	1024	26	26	25.7	26.1	30	29.9	29.8	29.7	27.5	26.7	30.3	35.4
43	1025	25.8	25.8	25.4	25.6	29.5	29.2	28.9	28.3	29.5	31.2	30.9	36.9
43	1026	25.6	25.5	24.8	25.1	29.5	29.2	28.9	28.3	31.3	35.3	30.8	36.6
43	1027	25.3	25.1	24.8	24.3	29.5	29.2	28.9	28.3	32.3	37.6	30.8	36.6
43	1028	25.3	25.1	24.1	24.3	29	28.5	28	27	32.2	37.4	30.5	35.9
43	1029	24.9	24.5	23.9	23.2	29	28.5	28	27	31.5	35.8	29.9	34.4
43	1030	24.8	24.4	23.9	23	28.5	27.8	27.1	25.7	30.6	33.8	29.1	32.6
43	1031	24.8	24.4	23.9	23	28.5	27.8	27.1	25.7	29.6	31.5	28.2	30.5
43	1032	24.8	24.4	24.6	23	29	28.5	28	27	28.5	29	27.4	28.7
44	1033	25.3	25	25.7	23.9	29.5	29.3	29.1	28.7	25.1	27.5	26.7	24

44	1034	25.9	25.8	26.6	25.5	30	30	30	30	24.8	26.7	26.1	22.5
44	1035	26.4	26.5	26.8	26.9	30.5	30.7	30.9	31.3	24.4	25.8	25.6	21.5
44	1036	26.5	26.7	27.3	27.1	31.5	32.1	32.7	34	24	24.9	25.3	20.8
44	1037	26.8	27.1	27.5	27.9	32	32.8	33.6	35.3	23.7	24.2	25	20.1
44	1038	26.9	27.2	28.4	28.2	32	32.8	33.6	35.3	23.3	23.4	24.7	19.4
44	1039	27.4	27.9	29.1	29.5	32	32.8	33.6	35.3	22.9	22.4	24.4	18.7
44	1040	27.8	28.5	29.1	30.6	32	32.8	33.6	35.3	22.5	21.4	24.1	17.9
44	1041	27.8	28.5	28.6	30.6	32	32.8	33.6	35.3	22.1	20.5	23.8	17.4
44	1042	27.5	28.1	28.6	29.8	31.5	32.1	32.7	34	21.7	19.8	23.7	17.1
44	1043	27.5	28.1	27.9	29.8	31.5	32.1	32.7	34	21.6	19.3	23.9	17.7
44	1044	27.1	27.5	27.3	28.7	31	31.4	31.8	32.7	21.9	20.1	24.6	19.1
44	1045	26.8	27.1	26.6	27.9	30.5	30.7	30.9	31.3	22.3	21.1	25.8	22
44	1046	26.4	26.5	26.1	26.9	30	30	30	30	22.7	21.9	28.4	28
44	1047	26.1	26.1	25.5	26.1	30	30	30	30	23	22.6	31.9	35.9
44	1048	25.8	25.7	25.2	25.3	29.5	29.3	29.1	28.7	23.8	24.6	34.4	41.6
44	1049	25.6	25.4	24.6	24.7	29	28.6	28.2	27.3	23.4	23.6	35.9	45.2
44	1050	25.3	25	24.5	23.9	28.5	27.9	27.3	26	23.3	23.3	36.7	47.1
44	1051	25.2	24.8	23.9	23.7	28.5	27.9	27.3	26	23.5	23.8	36.7	46.9
44	1052	24.9	24.4	23.7	22.9	28	27.2	26.4	24.7	24.1	25.2	35.5	44.4
44	1053	24.8	24.3	23	22.6	28	27.2	26.4	24.7	24.4	25.8	34	40.8
44	1054	24.4	23.7	23	21.5	27.5	26.5	25.5	23.4	24.1	25.3	32.5	37.5
44	1055	24.4	23.7	22.8	21.5	27.5	26.5	25.5	23.4	23.7	24.2	31.2	34.5
44	1056	24.3	23.6	23.9	21.3	27.5	26.5	25.5	23.4	23.1	23	29.4	30.2
45	1057	24.3	24.1	24.4	23.4	28	27.6	27.2	26.3	22.6	24.8	27.7	26.7
45	1058	24.6	24.5	25.1	24.2	28.5	28.3	28.1	27.6	22.1	23.7	26.2	23.3
45	1059	25	25.1	25.7	25.2	29.5	29.7	29.9	30.3	21.6	22.6	25	20.4
45	1060	25.3	25.5	26	26	30.5	31.1	31.7	32.9	21.2	21.7	23.9	17.8
45	1061	25.5	25.8	26.7	26.6	31.5	32.5	33.5	35.6	20.9	20.9	22.9	15.7
45	1062	25.9	26.3	27.6	27.6	32	33.2	34.4	36.9	20.6	20.2	22.1	13.8
45	1063	26.4	27	28.5	29	32	33.2	34.4	36.9	20.3	19.5	21.3	11.9
45	1064	26.9	27.7	28.7	30.3	32	33.2	34.4	36.9	20.1	19	20.6	10.3
45	1065	27	27.9	28.4	30.6	32	33.2	34.4	36.9	19.9	18.6	20.2	9.3
45	1066	26.8	27.6	27.8	30	26.5	25.5	24.5	22.3	19.8	18.3	20.6	10.3
45	1067	26.5	27.2	27.5	29.2	28.5	28.3	28.1	27.6	19.9	18.7	22.2	13.9
45	1068	26.3	26.9	26.6	28.7	29	29	29	28.9	19.7	18.2	24.6	19.5
45	1069	25.8	26.2	25.8	27.4	28.5	28.3	28.1	27.6	19.9	18.6	27.4	26
45	1070	25.4	25.6	24.9	26.3	28.5	28.3	28.1	27.6	20.6	20.1	31	34.3
45	1071	24.9	24.9	24.6	25	28.5	28.3	28.1	27.6	20.7	20.5	34	41.2
45	1072	24.7	24.6	24	24.5	28.5	28.3	28.1	27.6	21.1	21.4	36.3	46.4
45	1073	24.4	24.2	23.1	23.7	28.5	28.3	28.1	27.6	21.6	22.6	37.5	49.3
45	1074	23.9	23.5	22.2	22.3	28	27.6	27.2	26.3	21.8	23.1	37.8	49.8
45	1075	23.4	22.8	21.9	21	28	27.6	27.2	26.3	21.8	23.1	37.5	49.1
45	1076	23.2	22.5	21.3	20.5	28	27.6	27.2	26.3	21.6	22.6	36.4	46.5
45	1077	22.9	22.1	20.6	19.7	28	27.6	27.2	26.3	21.6	22.5	34.7	42.7
45	1078	22.5	21.6	20.4	18.6	27.5	26.9	26.3	24.9	21.4	22	33	38.8
45	1079	22.4	21.4	20.4	18.3	27.5	26.9	26.3	24.9	20.9	20.9	31.4	35.1
45	1080	22.4	21.4	22	18.3	27.5	26.9	26.3	24.9	20.3	19.5	29.9	31.8
46	1081	23.1	22.6	23.5	20.9	27.5	26.6	25.6	23.6	19.7	19	28.7	28.3
46	1082	23.9	23.7	25.1	23	28	27.3	26.5	24.9	19.3	17.9	27.6	25.7
46	1083	24.8	25	26.9	25.4	28.5	28	27.4	26.3	18.8	16.9	26.6	23.4
46	1084	25.8	26.4	28.7	28.1	29.5	29.4	29.2	28.9	18.4	15.8	25.6	21.2
46	1085	26.8	27.8	29.6	30.7	30.5	30.8	31	31.6	18	15	24.8	19.4
46	1086	27.3	28.5	29.8	32.1	31	31.5	31.9	32.9	17.6	14.1	24.1	17.6
46	1087	27.4	28.6	29.8	32.3	31.5	32.2	32.8	34.2	17.2	13.2	23.4	16.1
46	1088	27.4	28.6	28.9	32.3	31.5	32.2	32.8	34.2	16.8	12.2	22.7	14.6
46	1089	26.9	27.9	27.8	31	31	31.5	31.9	32.9	16.4	11.3	22.4	13.9
46	1090	26.3	27.1	27.1	29.4	31	31.5	31.9	32.9	16.2	10.8	22.5	14
46	1091	25.9	26.5	26.2	28.4	31	31.5	31.9	32.9	16.1	10.7	23.2	15.8
46	1092	25.4	25.8	25.3	27	31	31.5	31.9	32.9	16.5	11.6	25	19.8
46	1093	24.9	25.1	24.6	25.7	30.5	30.8	31	31.6	18.1	15.2	26.7	23.8
46	1094	24.5	24.5	23.8	24.6	30.5	30.8	31	31.6	19.9	19.4	30	31.3
46	1095	24.1	24	23.5	23.6	30	30.1	30.1	30.2	21.8	23.8	33.1	38.5
46	1096	23.9	23.7	22.6	23	30	30.1	30.1	30.2	23.3	27.1	35.3	43.5
46	1097	23.4	23	21.7	21.7	29.5	29.4	29.2	28.9	24.1	29	36.1	45.4
46	1098	22.9	22.3	21	20.4	29.5	29.4	29.2	28.9	24.6	30.2	36.4	45.9

46	1099	22.5	21.7	20.6	19.3	29.5	29.4	29.2	28.9	24.9	31	36.4	45.9
46	1100	22.3	21.5	19.9	18.8	29	28.7	28.3	27.6	25.1	31.3	35.6	44.2
46	1101	21.9	20.9	19.2	17.7	29	28.7	28.3	27.6	24.7	30.3	34.3	41.2
46	1102	21.5	20.3	19	16.7	29	28.7	28.3	27.6	23.9	28.6	33	38.2
46	1103	21.4	20.2	19.7	16.4	29	28.7	28.3	27.6	23.2	26.9	31.7	35.3
46	1104	21.8	20.8	21	17.4	29	28.7	28.3	27.6	22.4	25	30.6	32.6
47	1105	22.5	21.8	22.7	19.5	29.5	29.2	28.9	28.2	21.6	20.8	29.5	29
47	1106	23.4	23	24.5	21.9	29.5	29.2	28.9	28.2	21.1	19.5	28.6	26.9
47	1107	24.4	24.4	26.4	24.5	30	29.9	29.8	29.5	20.6	18.5	27.7	25
47	1108	25.5	26	28.2	27.5	30.5	30.6	30.7	30.8	20.1	17.3	26.9	23.1
47	1109	26.5	27.4	29.7	30.1	31	31.3	31.6	32.1	19.6	16.1	26.2	21.4
47	1110	27.3	28.5	29.7	32.3	31.5	32	32.5	33.5	19.1	15.1	25.5	19.8
47	1111	27.3	28.5	29.9	32.3	32	32.7	33.4	34.8	18.7	14.2	24.7	18.1
47	1112	27.4	28.6	28.8	32.5	31.5	32	32.5	33.5	18.4	13.4	24.1	16.5
47	1113	26.8	27.8	27.9	30.9	31.5	32	32.5	33.5	18.1	12.7	23.4	15.1
47	1114	26.3	27.1	27	29.6	31.5	32	32.5	33.5	17.9	12.3	23.4	15.1
47	1115	25.8	26.4	26.1	28.3	31	31.3	31.6	32.1	17.9	12.2	24.5	17.5
47	1116	25.3	25.7	25.4	26.9	31	31.3	31.6	32.1	18.3	13.1	26.7	22.5
47	1117	24.9	25.1	24.5	25.9	30.5	30.6	30.7	30.8	19.8	16.7	29.5	29
47	1118	24.4	24.4	23.7	24.5	30.5	30.6	30.7	30.8	21.7	21.1	31.9	34.6
47	1119	24	23.9	23.4	23.5	30.5	30.6	30.7	30.8	23.9	26	34.3	40.1
47	1120	23.8	23.6	22.7	22.9	30	29.9	29.8	29.5	25.8	30.5	35.6	43.1
47	1121	23.4	23	21.8	21.9	30	29.9	29.8	29.5	27.2	33.7	36	44.1
47	1122	22.9	22.3	21	20.5	30	29.9	29.8	29.5	27.3	33.8	36.3	44.6
47	1123	22.5	21.8	20.7	19.5	29.5	29.2	28.9	28.2	27.4	34.2	36.1	44.2
47	1124	22.3	21.5	20	19	29.5	29.2	28.9	28.2	27.2	33.6	35.4	42.6
47	1125	21.9	20.9	19.2	17.9	29.5	29.2	28.9	28.2	26.7	32.4	34.4	40.2
47	1126	21.5	20.4	19.2	16.8	29	28.5	28	26.8	25.9	30.6	33.2	37.6
47	1127	21.5	20.4	20.1	16.8	29	28.5	28	26.8	24.9	28.4	32	34.7
47	1128	22	21.1	21.8	18.2	29	28.5	28	26.8	23.9	26.1	30.7	31.9
48	1129	22.8	22.3	23.6	20.7	29.5	28.9	28.3	27	23.1	23.1	29.6	28.8
48	1130	23.8	23.7	25.4	23.3	30.5	30.3	30.1	29.6	22.3	21.4	28.6	26.3
48	1131	24.8	25.1	26.3	26	31	31	31	31	21.6	19.8	27.6	24.2
48	1132	25.3	25.8	27.2	27.3	31.5	31.7	31.9	32.3	21	18.4	26.7	22
48	1133	25.8	26.5	27.4	28.6	32	32.4	32.8	33.6	20.5	17.1	25.9	20.1
48	1134	25.9	26.6	27.5	28.9	32.5	33.1	33.7	35	20	16	25.1	18.4
48	1135	26	26.8	27.9	29.2	32.5	33.1	33.7	35	19.5	14.9	24.4	16.7
48	1136	26.2	27	27.2	29.7	32.5	33.1	33.7	35	19.1	13.9	23.7	15.1
48	1137	25.8	26.5	26.5	28.6	32.5	33.1	33.7	35	18.7	13	23.2	14
48	1138	25.4	25.9	26.3	27.6	32.5	33.1	33.7	35	18.4	12.3	23.2	14
48	1139	25.3	25.8	25.4	27.3	32	32.4	32.8	33.6	18.3	12.1	24.1	16.1
48	1140	24.8	25.1	24.7	26	32	32.4	32.8	33.6	18.6	12.7	26	20.4
48	1141	24.4	24.5	23.8	24.9	31.5	31.7	31.9	32.3	20.1	16.2	28.8	26.9
48	1142	23.9	23.8	23	23.6	31.5	31.7	31.9	32.3	21.7	20.1	31.8	33.8
48	1143	23.5	23.3	22.9	22.5	31	31	31	31	23.6	24.2	34.5	40
48	1144	23.4	23.1	22.1	22.3	30.5	30.3	30.1	29.6	25.3	28.3	36.5	44.7
48	1145	23	22.6	22	21.2	30.5	30.3	30.1	29.6	26.8	31.7	37.8	47.7
48	1146	22.9	22.4	21.8	20.9	30.5	30.3	30.1	29.6	27.9	34.3	38.5	49.3
48	1147	22.8	22.3	21.1	20.7	30	29.6	29.2	28.3	28.6	35.8	38.2	48.4
48	1148	22.4	21.7	21.1	19.6	30	29.6	29.2	28.3	28.8	36.3	37.2	46.3
48	1149	22.4	21.7	20.9	19.6	29.5	28.9	28.3	27	28.4	35.3	35.9	43.3
48	1150	22.3	21.6	20.9	19.3	29.5	28.9	28.3	27	27.7	33.7	34.4	39.8
48	1151	22.3	21.6	21.8	19.3	29.5	28.9	28.3	27	26.8	31.7	33	36.7
48	1152	22.8	22.3	22	20.7	29.5	28.9	28.3	27	25.8	29.5	31.8	33.8
49	1153	23.5	22.8	23.6	20.4	30	29.4	28.9	27.7	25	24.7	30.7	36.8
49	1154	24.4	24	25.3	22.8	30.5	30.1	29.8	29	24.2	22.9	29.8	34.7
49	1155	25.3	25.3	27.4	25.2	31	30.8	30.7	30.3	23.5	21.4	29	32.8
49	1156	26.5	27	29.2	28.4	31.5	31.5	31.6	31.7	22.9	20	28.4	31.4
49	1157	27.5	28.4	30.7	31.1	32.5	32.9	33.4	34.3	22.4	18.8	27.7	29.9
49	1158	28.3	29.5	31.6	33.2	33	33.6	34.3	35.7	21.9	17.6	27.2	28.6
49	1159	28.8	30.2	31.2	34.5	33	33.6	34.3	35.7	21.4	16.6	26.7	27.6
49	1160	28.6	29.9	30.1	34	33	33.6	34.3	35.7	21	15.6	26.2	26.5
49	1161	28	29.1	28.9	32.4	32.5	32.9	33.4	34.3	20.6	14.7	25.7	25.2
49	1162	27.3	28.1	28	30.5	32.5	32.9	33.4	34.3	20.3	14	25.2	24.1
49	1163	26.8	27.4	27.2	29.2	32.5	32.9	33.4	34.3	20.2	13.8	24	21.3

49	1164	26.4	26.8	26.3	28.1	32	32.2	32.5	33	20.5	14.5	23.5	20.2
49	1165	25.9	26.1	25.4	26.8	32	32.2	32.5	33	22.1	18.1	23.4	20
49	1166	25.4	25.4	24.7	25.5	31.5	31.5	31.6	31.7	24.1	22.7	23.5	20.2
49	1167	25	24.9	24.4	24.4	31.5	31.5	31.6	31.7	26.1	27.3	23.2	19.6
49	1168	24.8	24.6	23.6	23.9	31	30.8	30.7	30.3	27.9	31.5	25.4	24.5
49	1169	24.4	24	22.7	22.8	31	30.8	30.7	30.3	29.4	34.9	27	28.2
49	1170	23.9	23.3	22	21.5	31	30.8	30.7	30.3	30.4	37.2	27.9	30.2
49	1171	23.5	22.8	21.8	20.4	30.5	30.1	29.8	29	31	38.6	27.9	30.2
49	1172	23.4	22.6	20.9	20.2	30.5	30.1	29.8	29	31	38.5	26.9	28.1
49	1173	22.9	21.9	20.6	18.8	30.5	30.1	29.8	29	30.6	37.6	25.6	25.1
49	1174	22.7	21.6	20	18.3	30	29.4	28.9	27.7	29.8	35.9	24.4	22.3
49	1175	22.4	21.2	20.8	17.5	30	29.4	28.9	27.7	28.9	33.8	23.4	19.9
49	1176	22.8	21.8	22.1	18.6	30	29.4	28.9	27.7	28	31.6	22.4	17.6
50	1177	23.8	23	23.9	20.3	30.5	30.3	30	29.5	27.1	27.4	21.5	16.3
50	1178	24.8	24.4	25.7	23	30.5	30.3	30	29.5	26.3	25.7	20.6	14.3
50	1179	25.8	25.8	27.9	25.7	31	31	30.9	30.8	25.6	24.1	19.9	12.7
50	1180	27	27.4	29.7	28.9	31.5	31.7	31.8	32.2	25	22.7	19.4	11.4
50	1181	28	28.8	31.1	31.5	32	32.4	32.7	33.5	24.5	21.4	18.8	10.1
50	1182	28.8	30	31.5	33.6	32.5	33.1	33.6	34.8	24	20.2	18.5	9.4
50	1183	29	30.2	31.3	34.2	33	33.8	34.5	36.1	23.4	19.1	18.2	8.8
50	1184	28.9	30.1	30.4	33.9	33	33.8	34.5	36.1	23	18.1	18	8.2
50	1185	28.4	29.4	29.3	32.6	32.5	33.1	33.6	34.8	22.6	17.2	17.9	8.1
50	1186	27.8	28.6	28.4	31	32.5	33.1	33.6	34.8	22.3	16.3	18.5	9.5
50	1187	27.3	27.9	27.5	29.7	32.5	33.1	33.6	34.8	22.1	15.9	20.6	14.3
50	1188	26.8	27.2	26.8	28.3	32	32.4	32.7	33.5	22.3	16.4	23.7	21.3
50	1189	26.4	26.6	25.9	27.3	32	32.4	32.7	33.5	23.7	19.6	26.9	28.8
50	1190	25.9	25.9	25.4	25.9	31.5	31.7	31.8	32.2	25.5	23.8	30.2	36.4
50	1191	25.6	25.5	24.8	25.1	31.5	31.7	31.8	32.2	27.4	28.2	32.3	41.1
50	1192	25.3	25.1	24.1	24.3	31	31	30.9	30.8	29.3	32.5	33.4	43.7
50	1193	24.9	24.5	23.4	23.3	30.5	30.3	30	29.5	30.8	36.1	33.7	44.3
50	1194	24.5	23.9	23.2	22.2	30.5	30.3	30	29.5	31.9	38.6	34.4	46
50	1195	24.4	23.8	22.3	21.9	30	29.6	29.1	28.2	32.5	39.9	34.5	46.2
50	1196	23.9	23.1	21.6	20.6	29.5	28.9	28.2	26.8	32.2	39.2	33.3	43.4
50	1197	23.5	22.5	21.4	19.6	29.5	28.9	28.2	26.8	31.7	38	31.6	39.6
50	1198	23.4	22.4	21.2	19.3	29	28.2	27.3	25.5	30.8	36.1	30	35.8
50	1199	23.3	22.3	21.8	19	29	28.2	27.3	25.5	29.9	34	28.6	32.5
50	1200	23.6	22.7	22.6	19.8	29	28.2	27.3	25.5	29	31.8	27.2	29.5
51	1201	24.4	23.5	24.3	20.8	29.5	29.1	28.7	27.9	28.2	29.3	26.1	23.2
51	1202	25.3	24.8	26.4	23.1	30	29.8	29.6	29.2	27.5	27.6	25.1	20.9
51	1203	26.5	26.5	28.6	26.3	30.5	30.5	30.5	30.6	26.9	26.3	24.2	18.9
51	1204	27.7	28.1	30.4	29.5	31.5	31.9	32.3	33.2	26.4	25	23.4	17
51	1205	28.7	29.5	31.8	32.2	32	32.6	33.2	34.6	25.7	23.6	22.7	15.3
51	1206	29.5	30.7	32.4	34.3	32	32.6	33.2	34.6	25.2	22.3	22.1	13.8
51	1207	29.8	31.1	32.4	35.1	32	32.6	33.2	34.6	24.7	21.1	21.5	12.5
51	1208	29.8	31.1	30.9	35.1	32	32.6	33.2	34.6	24.2	20	20.9	11.2
51	1209	29	30	30	33	31.5	31.9	32.3	33.2	23.9	19.3	20.7	10.7
51	1210	28.5	29.3	29.3	31.7	31.5	31.9	32.3	33.2	23.7	18.8	21.2	11.8
51	1211	28.1	28.7	28.4	30.6	31	31.2	31.4	31.9	23.6	18.6	23.1	16.1
51	1212	27.6	28	27.9	29.3	31	31.2	31.4	31.9	23.6	18.6	26.2	23.3
51	1213	27.3	27.6	27	28.5	30.5	30.5	30.5	30.6	23.9	19.3	28.8	29.4
51	1214	26.8	26.9	26.2	27.1	30.5	30.5	30.5	30.6	25.2	22.3	31	34.5
51	1215	26.4	26.3	25.3	26.1	30.5	30.5	30.5	30.6	27.1	26.7	34	41.4
51	1216	25.9	25.6	24.8	24.7	30	29.8	29.6	29.2	28.9	30.9	36.4	46.9
51	1217	25.6	25.2	24.3	23.9	30	29.8	29.6	29.2	30.5	34.4	37.7	49.9
51	1218	25.3	24.8	23.5	23.1	29.5	29.1	28.7	27.9	31.6	36.9	37.7	49.7
51	1219	24.9	24.2	23	22.1	29.5	29.1	28.7	27.9	32.2	38.4	36.3	46.6
51	1220	24.6	23.8	22.6	21.3	29.5	29.1	28.7	27.9	32.3	38.5	35.1	43.9
51	1221	24.4	23.5	21.9	20.8	29.5	29.1	28.7	27.9	31.9	37.7	33.7	40.7
51	1222	24	23	21.7	19.7	29	28.4	27.8	26.6	31.2	36.1	32.4	37.5
51	1223	23.9	22.8	22.5	19.4	29	28.4	27.8	26.6	30.3	34	31	34.4
51	1224	24.3	23.4	23.3	20.5	29	28.4	27.8	26.6	29.5	32.1	29.7	31.5
52	1225	25	24.2	25.3	21.5	29	28.1	27.2	25.3	28.7	31.3	28.7	28.3
52	1226	26.1	25.7	27.5	24.5	29.5	28.8	28.1	26.7	28	29.7	27.8	26.3
52	1227	27.3	27.4	29.6	27.7	30	29.5	29	28	27.4	28.3	26.9	24.2
52	1228	28.5	29.1	31.3	30.9	31	30.9	30.8	30.7	26.9	27.1	26.2	22.5

52	1229	29.4	30.3	32.3	33.3	31.5	31.6	31.7	32	26.3	25.7	25.4	20.8
52	1230	30	31.2	33.6	34.8	32	32.3	32.6	33.3	25.7	24.4	24.7	19.2
52	1231	30.7	32.1	33.1	36.7	32	32.3	32.6	33.3	25.3	23.3	24.2	18.1
52	1232	30.4	31.7	32.3	35.9	32	32.3	32.6	33.3	24.8	22.3	23.7	16.9
52	1233	30	31.2	31.4	34.8	32	32.3	32.6	33.3	24.5	21.6	23.3	15.9
52	1234	29.5	30.5	31.1	33.5	32	32.3	32.6	33.3	24.2	20.9	23.6	16.6
52	1235	29.3	30.2	30.2	33	32	32.3	32.6	33.3	23.9	20.2	25.1	19.9
52	1236	28.8	29.5	29.3	31.7	32	32.3	32.6	33.3	23.9	20.2	26.9	24.2
52	1237	28.3	28.8	28.4	30.3	32	32.3	32.6	33.3	24.4	21.3	28.5	27.8
52	1238	27.8	28.1	27.8	29	31.5	31.6	31.7	32	25.1	23	30.2	31.9
52	1239	27.5	27.7	27.5	28.2	31.5	31.6	31.7	32	26.3	25.8	32	35.9
52	1240	27.3	27.4	21.2	27.7	31.5	31.6	31.7	32	27.3	28.1	33.5	39.3
52	1241	23.8	22.5	20.8	18.4	31.5	31.6	31.7	32	28.3	30.3	34.3	41.2
52	1242	23.6	22.2	22.3	17.8	31	30.9	30.8	30.7	29	31.8	35.1	43.1
52	1243	24.4	23.3	22.3	20	31	30.9	30.8	30.7	29.2	32.3	35.2	43.3
52	1244	24.4	23.3	22.3	20	31	30.9	30.8	30.7	29.4	32.7	34.5	41.8
52	1245	24.4	23.3	22.3	20	31	30.9	30.8	30.7	29.3	32.5	33.3	38.9
52	1246	24.4	23.3	22.3	20	30.5	30.2	29.9	29.3	28.9	31.7	32	35.9
52	1247	24.4	23.3	22.6	20	30.5	30.2	29.9	29.3	28.3	30.2	30.8	33.2
52	1248	24.6	23.6	24.5	20.5	31	30.9	30.8	30.7	27.6	28.8	29.7	30.7
53	1249	25	24.7	25	23.9	31	30.3	29.6	28	27	28.4	28.8	32.8
53	1250	25.3	25.2	25.4	24.7	31.5	31	30.5	29.3	26.5	27.2	28.1	31.1
53	1251	25.5	25.4	26.1	25.3	32.5	32.4	32.3	32	26	26.1	27.4	29.5
53	1252	25.9	26	26.8	26.3	33	33.1	33.2	33.3	25.6	25.1	26.7	28.1
53	1253	26.3	26.6	27	27.4	34	34.5	35	36	25.2	24.3	26.1	26.6
53	1254	26.4	26.7	27.2	27.7	34	34.5	35	36	24.9	23.5	25.6	25.3
53	1255	26.5	26.8	27.9	27.9	34	34.5	35	36	24.6	22.9	25.2	24.6
53	1256	26.9	27.4	28.1	29	34	34.5	35	36	24.3	22.2	24.9	23.9
53	1257	27	27.5	27.7	29.3	34	34.5	35	36	24.1	21.7	24.7	23.5
53	1258	26.8	27.3	27.4	28.7	34	34.5	35	36	23.9	21.2	24.9	23.7
53	1259	26.6	27	26.8	28.2	33.5	33.8	34.1	34.6	23.8	21	25.3	24.7
53	1260	26.3	26.6	26.5	27.4	33.5	33.8	34.1	34.6	23.8	21.1	26.1	26.6
53	1261	26.1	26.3	25.9	26.9	33.5	33.8	34.1	34.6	24.1	21.7	27.5	29.8
53	1262	25.8	25.9	25.9	26.1	33	33.1	33.2	33.3	24.7	23.1	29.3	33.9
53	1263	25.8	25.9	25.2	26.1	33	33.1	33.2	33.3	25.3	24.5	28.4	31.9
53	1264	25.4	25.3	25	25	33	33.1	33.2	33.3	26.7	27.6	23.5	20.6
53	1265	25.3	25.2	24.7	24.7	32.5	32.4	32.3	32	27.2	28.8	25.3	24.7
53	1266	25.1	24.9	24.3	24.2	32.5	32.4	32.3	32	27.5	29.6	25.4	25
53	1267	24.9	24.6	24.1	23.7	32.5	32.4	32.3	32	27.9	30.3	25.1	24.3
53	1268	24.8	24.5	23.4	23.4	32	31.7	31.4	30.7	28.2	31.2	25.2	24.6
53	1269	24.4	23.9	23.4	22.3	32	31.7	31.4	30.7	28.5	31.7	24.4	22.7
53	1270	24.4	23.9	23.4	22.3	31.5	31	30.5	29.3	28.3	31.3	23.7	21
53	1271	24.4	23.9	23.6	22.3	31.5	31	30.5	29.3	27.8	30.2	23.1	19.7
53	1272	24.5	24	23.4	22.6	31.5	31	30.5	29.3	27.1	28.6	22.7	18.9
54	1273	24.8	24.1	24.7	21.9	31.5	31.5	31.6	31.6	26.5	26.3	22.5	17.9
54	1274	25.5	25.1	26.1	23.7	31.5	31.5	31.6	31.6	25.9	25	22.2	17.2
54	1275	26.3	26.2	28.1	25.9	31.5	31.5	31.6	31.6	25.4	23.9	21.9	16.6
54	1276	27.4	27.7	29.7	28.8	32	32.2	32.5	33	25	23	21.7	16.1
54	1277	28.3	29	30.6	31.2	32.5	32.9	33.4	34.3	24.7	22.2	21.3	15.1
54	1278	28.8	29.7	30.8	32.5	33	33.6	34.3	35.6	24.4	21.4	20.9	14.1
54	1279	28.9	29.8	30.8	32.8	33	33.6	34.3	35.6	24.1	20.7	20.4	13
54	1280	28.9	29.8	30.1	32.8	33	33.6	34.3	35.6	23.8	20.1	19.9	11.8
54	1281	28.5	29.3	29.2	31.7	33	33.6	34.3	35.6	23.6	19.7	19.6	11.3
54	1282	28	28.6	28.4	30.4	32.5	32.9	33.4	34.3	23.4	19.3	20.3	12.8
54	1283	27.6	28	27.9	29.3	32.5	32.9	33.4	34.3	23.4	19.1	22.1	17
54	1284	27.3	27.6	27	28.5	32.5	32.9	33.4	34.3	23.5	19.5	24.6	22.6
54	1285	26.8	26.9	26.5	27.2	32	32.2	32.5	33	24.1	20.8	27.1	28.4
54	1286	26.5	26.5	26.1	26.4	32	32.2	32.5	33	25.1	23.1	29.4	33.8
54	1287	26.3	26.2	25.4	25.9	31.5	31.5	31.6	31.6	26.8	27	31.7	39
54	1288	25.9	25.6	25.2	24.8	31	30.8	30.7	30.3	28.3	30.5	33.9	44.2
54	1289	25.8	25.5	24.7	24.5	31	30.8	30.7	30.3	29.6	33.4	35.2	47
54	1290	25.5	25.1	24.5	23.7	30.5	30.1	29.8	29	30.8	36.3	35.4	47.6
54	1291	25.4	24.9	24.3	23.5	30	29.4	28.9	27.6	31.5	37.9	34.2	44.9
54	1292	25.3	24.8	23.9	23.2	30	29.4	28.9	27.6	31.8	38.6	32	39.9
54	1293	25.1	24.5	23.6	22.7	29.5	28.7	28	26.3	30.8	36.2	29.9	34.8

54	1294	24.9	24.2	23.4	22.1	29.5	28.7	28	26.3	29.6	33.6	27.9	30.4
54	1295	24.8	24.1	23.4	21.9	29	28	27.1	25	28.7	31.3	26.2	26.5
54	1296	24.8	24.1	24.7	21.9	29.5	28.7	28	26.3	27.9	29.4	24.7	23
55	1297	25.3	25	25.6	24.1	29.5	29.1	28.6	27.7	27.3	29.5	23.5	26.9
55	1298	25.8	25.7	26	25.4	30	29.8	29.5	29	26.6	28	22.4	24.5
55	1299	26	26	26.9	26	30.5	30.5	30.4	30.3	25.9	26.4	21.6	22.6
55	1300	26.5	26.7	27.6	27.3	31.5	31.9	32.2	33	25.3	25	20.9	20.9
55	1301	26.9	27.3	28.5	28.4	32	32.6	33.1	34.3	24.7	23.6	20.2	19.3
55	1302	27.4	28	28.7	29.7	32	32.6	33.1	34.3	24.2	22.5	19.7	18.1
55	1303	27.5	28.1	29.2	29.9	32	32.6	33.1	34.3	23.8	21.5	19.3	17.3
55	1304	27.8	28.5	29.2	30.7	32	32.6	33.1	34.3	23.4	20.6	19	16.6
55	1305	27.8	28.5	28.7	30.7	32	32.6	33.1	34.3	23	19.8	19	16.6
55	1306	27.5	28.1	28.3	29.9	32	32.6	33.1	34.3	22.8	19.2	19.4	17.6
55	1307	27.3	27.8	27.4	29.4	32	32.6	33.1	34.3	22.7	18.9	19.7	18.1
55	1308	26.8	27.1	27.1	28.1	31.5	31.9	32.2	33	22.7	18.9	19.8	18.4
55	1309	26.6	26.8	26.5	27.6	31	31.2	31.3	31.7	23	19.6	20.2	19.3
55	1310	26.3	26.4	25.8	26.8	31	31.2	31.3	31.7	23.5	20.9	21	21.2
55	1311	25.9	25.9	25.6	25.7	30.5	30.5	30.4	30.3	24.4	23	21.2	21.7
55	1312	25.8	25.7	24.9	25.4	30.5	30.5	30.4	30.3	25.2	24.8	21.4	22.2
55	1313	25.4	25.2	24.5	24.4	30	29.8	29.5	29	26.3	27.2	21.8	23
55	1314	25.2	24.9	24	23.8	30	29.8	29.5	29	27.5	30	22.2	23.9
55	1315	24.9	24.5	23.5	23	29.5	29.1	28.6	27.7	28.7	32.8	22.3	24.2
55	1316	24.6	24	23.1	22.2	29.5	29.1	28.6	27.7	29.3	34.1	22.4	24.5
55	1317	24.4	23.8	22.7	21.7	29	28.4	27.7	26.3	29.3	34.1	22.1	23.6
55	1318	24.2	23.5	22.6	21.2	29	28.4	27.7	26.3	28.8	33	21.4	22
55	1319	24.1	23.3	23.5	20.9	28.5	27.7	26.8	25	28	31.3	20.5	20
55	1320	24.6	24	24	22.2	29	28.4	27.7	26.3	27.3	29.5	19.7	18.3
56	1321	25	24.5	25.6	22.9		28.7	28.3	27.6	26.6	31.8	19	16.6
56	1322	25.9	25.8	27.2	25.3		29.4	29.2	28.9	26.1	30.6	18.5	15.3
56	1323	26.8	27	29	27.7		30.8	31	31.6	22.8	23.2	18.2	14.6
56	1324	27.8	28.4	30.5	30.3		31.5	31.9	32.9	21.8	20.7	18	14.2
56	1325	28.6	29.5	31.4	32.5		32.2	32.8	34.3	21.5	20	17.8	13.7
56	1326	29.1	30.2	31.7	33.8		32.9	33.7	35.6	21.1	19.2	17.8	13.7
56	1327	29.3	30.5	31.5	34.3		32.9	33.7	35.6	20.7	18.2	17.8	13.7
56	1328	29.2	30.4	30.3	34.1		32.2	32.8	34.3	20.2	17	17.9	14
56	1329	28.5	29.4	29.4	32.2		32.2	32.8	34.3	20	16.5	18	14.2
56	1330	28	28.7	28.5	30.9		31.5	31.9	32.9	19.7	16	18.9	16.3
56	1331	27.5	28	27.8	29.5		31.5	31.9	32.9	19.5	15.5	20.6	20.1
56	1332	27.1	27.4	27.2	28.5		30.8	31	31.6	19.5	15.5	20.9	20.9
56	1333	26.8	27	26.3	27.7		30.8	31	31.6	20.4	17.5	21.9	23.1
56	1334	26.3	26.3	25.6	26.3		30.1	30.1	30.3	20.8	18.4	22.8	25.3
56	1335	25.9	25.8	24.7	25.3		30.1	30.1	30.3	21.4	19.9	22.4	24.4
56	1336	25.4	25.1	24	23.9		29.4	29.2	28.9	23.3	24.3	24.9	30
56	1337	25	24.5	23.8	22.9		28.7	28.3	27.6	24.8	27.7	26.3	33.3
56	1338	24.9	24.4	22.9	22.6		28.7	28.3	27.6	25.2	28.6	25.9	32.4
56	1339	24.4	23.7	22.7	21.3		28	27.4	26.3	25.6	29.4	25.3	31
56	1340	24.3	23.5	22	21		27.3	26.5	25	25.6	29.6	23.7	27.4
56	1341	23.9	23	21.3	20		27.3	26.5	25	25.5	29.3	22.6	24.8
56	1342	23.5	22.4	21.3	18.9		26.6	25.6	23.6	24.9	27.9	21.7	22.7
56	1343	23.5	22.4	22	18.9		26.6	25.6	23.6	24.2	26.3	20.9	20.8
56	1344	23.9	23	23.3	20		26.6	25.6	23.6	23.4	24.4	20.2	19.2
57	1345	24.6	23.9	24.7	21.8		27.6	27.1	26.1	22.7	21.5	19.5	20.2
57	1346	25.4	25.1	26.5	23.9		28.3	28	27.5	22.1	20.1	18.9	19
57	1347	26.4	26.5	28.1	26.6		29	28.9	28.8	21.6	18.9	18.3	17.5
57	1348	27.3	27.7	29.4	29		30.4	30.7	31.5	21.1	17.7	17.6	15.9
57	1349	28	28.7	30.3	30.9		31.1	31.6	32.8	20.7	16.8	17	14.5
57	1350	28.5	29.4	30.8	32.2		31.1	31.6	32.8	20.4	16.1	16.4	13.2
57	1351	28.8	29.8	30.8	33		31.8	32.5	34.1	20.1	15.5	15.9	12
57	1352	28.8	29.8	29.9	33		31.1	31.6	32.8	19.8	14.8	15.8	11.8
57	1353	28.3	29.1	29	31.7		31.1	31.6	32.8	19.5	14.2	15.9	12
57	1354	27.8	28.4	28.1	30.3		31.1	31.6	32.8	19.3	13.6	16.2	12.8
57	1355	27.3	27.7	27.8	29		30.4	30.7	31.5	19.1	13.2	16.7	13.9
57	1356	27.1	27.4	27.2	28.5		30.4	30.7	31.5	19.5	14	17.7	16.2
57	1357	26.8	27	26.5	27.7		29.7	29.8	30.1	21.1	17.7	18.5	18.1
57	1358	26.4	26.5	25.8	26.6		29.7	29.8	30.1	22.8	21.8	19.6	20.4

57	1359	26	25.9	25.4	25.5		29	28.9	28.8	24.6	25.9	21.9	25.7
57	1360	25.8	25.6	24.7	25		29	28.9	28.8	26.4	29.9	22.9	28
57	1361	25.4	25.1	24.5	23.9		28.3	28	27.5	27.8	33.3	22.7	27.7
57	1362	25.3	24.9	23.8	23.7		28.3	28	27.5	28.8	35.6	22.1	26.1
57	1363	24.9	24.4	23.1	22.6		27.6	27.1	26.1	29.3	36.8	21.6	25.1
57	1364	24.5	23.8	22.9	21.6		27.6	27.1	26.1	29.4	36.8	20.8	23.3
57	1365	24.4	23.7	22.4	21.3		26.9	26.2	24.8	28.9	35.7	20.2	22
57	1366	24.1	23.2	22.4	20.5		26.9	26.2	24.8	28	33.8	19.7	20.7
57	1367	24.1	23.2	23.3	20.5		26.9	26.2	24.8	27.1	31.6	19.2	19.5
57	1368	24.6	23.9	23.4	21.8		26.9	26.2	24.8	26.1	29.4	18.5	17.9
58	1369	25	24.2	25	21.7		27.5	27.4		25.3	24.9	17.7	12.1
58	1370	25.9	25.5	27.2	24.1		27.5	27.4		24.5	23.2	17	10.4
58	1371	27.1	27.2	29.2	27.3		27.5	27.4		23.9	21.8	16.4	8.9
58	1372	28.2	28.7	30.6	30.2		27.5	27.4		23.4	20.6	15.8	7.7
58	1373	29	29.8	32.1	32.4		27.5	27.4		22.9	19.4	15.2	6.4
58	1374	29.8	30.9	32.1	34.5		28.2	28.3		22.4	18.4	14.9	5.5
58	1375	29.8	30.9	31.9	34.5		28.2	28.3		22	17.4	14.5	4.6
58	1376	29.7	30.8	30.6	34.2		28.2	28.3		21.6	16.5	14.2	4.1
58	1377	29	29.8	30.3	32.4		28.2	28.3		21.3	15.8	14.3	4.2
58	1378	28.8	29.5	29.4	31.8		28.2	28.3		21	15.2	15.3	6.5
58	1379	28.3	28.8	28.5	30.5		28.2	28.3		20.9	14.9	17.8	12.3
58	1380	27.8	28.1	27.7	29.2		28.2	28.3		21.2	15.5	21.1	19.9
58	1381	27.4	27.6	27	28.1		28.2	28.3		22.7	19	24.4	27.5
58	1382	27	27	26.7	27		28.2	28.3		24.6	23.4	26.9	33.1
58	1383	26.8	26.7	25.9	26.5		28.2	28.3		26.6	28	28.9	37.7
58	1384	26.4	26.2	25	25.4		27.5	27.4		28.4	32.2	30	40.3
58	1385	25.9	25.5	24.9	24.1		27.5	27.4		29.9	35.7	31.5	43.9
58	1386	25.8	25.3	24.1	23.8		27.5	27.4		30.4	36.8	31.8	44.5
58	1387	25.4	24.8	23.8	22.8		27.5	27.4		31	38.2	30.7	41.9
58	1388	25.2	24.5	23.2	22.3		26.8	26.5		31.1	38.3	29.3	38.7
58	1389	24.9	24.1	22.7	21.5		26.8	26.5		30.6	37.2	27.7	35.1
58	1390	24.6	23.7	22.7	20.7		26.8	26.5		29.8	35.3	26.1	31.4
58	1391	24.6	23.7	23.4	20.7		26.8	26.5		28.8	33.1	24.6	27.8
58	1392	25	24.2	24.1	21.7		26.8	26.5		27.9	30.9	23.3	24.9
59	1393	25.5	24.8	25.5	22.5		26.7	26.4		27	27.1	22.2	21.5
59	1394	26.3	25.9	27.7	24.6		26.7	26.4		26.3	25.4	21.3	19.5
59	1395	27.5	27.6	29.1	27.8		27.4	27.3		25.6	23.8	20.6	17.8
59	1396	28.3	28.7	30	29.9		28.8	29.1		25	22.5	19.9	16.2
59	1397	28.8	29.4	30.9	31.3		29.5	30		24.5	21.2	19.4	15.1
59	1398	29.3	30.1	31.1	32.6		30.2	30.9		24	20.1	18.9	13.9
59	1399	29.4	30.2	31.1	32.9		30.2	30.9		23.5	19	18.4	12.8
59	1400	29.4	30.2	30.9	32.9		30.2	30.9		23.1	18.1	17.9	11.8
59	1401	29.3	30.1	30.4	32.6		30.2	30.9		22.7	17.2	18	11.9
59	1402	29	29.7	30	31.8		29.5	30		22.4	16.5	18.6	13.3
59	1403	28.8	29.4	29.1	31.3		29.5	30		22.3	16.2	19.5	15.4
59	1404	28.3	28.7	28.4	29.9		28.8	29.1		22.5	16.7	21.7	20.5
59	1405	27.9	28.1	27.8	28.9		28.8	29.1		23.9	19.9	24.6	27.1
59	1406	27.6	27.7	27.3	28.1		28.1	28.2		25.7	24.2	26.2	30.9
59	1407	27.3	27.3	26.6	27.3		28.1	28.2		27.7	28.7	26.8	32.2
59	1408	26.9	26.7	26.2	26.2		27.4	27.3		29.6	33.1	26.9	32.3
59	1409	26.7	26.5	25.7	25.7		27.4	27.3		31.1	36.6	27.1	32.9
59	1410	26.4	26	24.8	24.9		26.7	26.4		32.1	38.8	27.5	33.8
59	1411	25.9	25.3	24.6	23.6		26.7	26.4		32.6	40	26.6	31.6
59	1412	25.8	25.2	23.9	23.3		26	25.5		32.5	39.7	25.9	30
59	1413	25.4	24.6	23.7	22.2		25.3	24.6		32	38.5	25.3	28.7
59	1414	25.3	24.5	23.2	22		25.3	24.6		31.2	36.6	24.4	26.7
59	1415	25	24.1	23.7	21.2		25.3	24.6		30.1	34.2	23.6	24.8
59	1416	25.3	24.5	24.7	22		25.3	24.6		29.1	31.9	22.9	23.3
60	1417	25.8	25.3	26	23.6		25.4	24.7		28.2	29.1	21.9	22.5
60	1418	26.5	26.2	27.6	25.5		26.1	25.6		27.4	27.2	20.7	19.8
60	1419	27.4	27.5	29.6	27.8		26.8	26.5		26.7	25.7	20.2	18.6
60	1420	28.5	29	31	30.8		28.2	28.3		26.1	24.3	19.9	17.9
60	1421	29.3	30.2	31.9	32.9		28.9	29.2		25.6	23	19.7	17.4
60	1422	29.8	30.9	31.9	34.2		29.6	30.1		25.1	21.9	19.4	16.8
60	1423	29.8	30.9	31.8	34.2		29.6	30.1		24.6	20.8	19.1	16.1

60	1424	29.7	30.7	31	34		29.6	30.1		24.2	19.8	18.7	15
60	1425	29.3	30.2	30.1	32.9		28.9	29.2		23.7	18.8	18.5	14.8
60	1426	28.8	29.5	29.2	31.6		28.9	29.2		23.4	17.9	19.2	16.2
60	1427	28.3	28.8	28.5	30.2		28.9	29.2		23.1	17.4	20.1	18.2
60	1428	27.9	28.2	27.8	29.2		28.2	28.3		23.3	17.8	21.1	20.7
60	1429	27.5	27.6	27.4	28.1		28.2	28.3		24.7	21	22.7	24.3
60	1430	27.3	27.4	26.5	27.6		28.2	28.3		26.5	25.1	23.9	27
60	1431	26.8	26.7	25.8	26.3		27.5	27.4		28.3	29.3	24.7	28.9
60	1432	26.4	26.1	25.3	25.2		27.5	27.4		30	33.2	24.4	28.3
60	1433	26.1	25.7	24.9	24.4		27.5	27.4		31.3	36.2	23.9	27.1
60	1434	25.9	25.4	24	23.9		26.8	26.5		32.3	38.4	23.7	26.6
60	1435	25.4	24.7	23.8	22.5		26.8	26.5		32.5	39	23.8	26.8
60	1436	25.3	24.6	23.1	22.3		26.8	26.5		32.4	38.7	23.4	26
60	1437	24.9	24	22.9	21.2		26.8	26.5		31.8	37.3	22.6	24
60	1438	24.8	23.9	22.9	20.9		26.1	25.6		30.8	35.2	21.9	22.4
60	1439	24.8	23.9	22.9	20.9		26.1	25.6		29.9	33	21.1	20.7
60	1440	24.8	23.9	24.4	20.9		26.1	25.6		29	30.9	20.5	19.2
61	1441	24.8	24.6	24.7	23.9		26.8	26.6		28.2	31.1	20.1	16
61	1442	25	24.9	24.9	24.4		26.8	26.6		27.6	29.6	19.7	15.3
61	1443	25.1	25	24.9	24.7		27.5	27.5		27.1	28.5	19.4	14.4
61	1444	25.1	25	25.3	24.7		27.5	27.5		26.6	27.5	18.9	13.4
61	1445	25.3	25.3	26.2	25.2		28.2	28.4		26.2	26.6	18.5	12.4
61	1446	25.8	26	26.5	26.6		28.2	28.4		25.9	25.8	18	11.2
61	1447	26	26.3	26.7	27.1		28.2	28.4		25.5	24.9	17.6	10.4
61	1448	26.1	26.4	27.1	27.4		28.2	28.4		25.2	24.1	17.4	9.8
61	1449	26.3	26.7	26.7	27.9		28.2	28.4		24.7	23.1	17.4	9.9
61	1450	26.1	26.4	26.7	27.4		28.2	28.4		24.4	22.3	17.7	10.7
61	1451	26.1	26.4	26.2	27.4		28.2	28.4		24.4	22.3	18.4	12.2
61	1452	25.8	26	26.2	26.6		28.2	28.4		24.5	22.7	20.6	17.3
61	1453	25.8	26	25.6	26.6			28.4		25.1	24	23.8	24.6
61	1454	25.5	25.6	25.3	25.8			28.4		25.7	25.4	26.6	31.1
61	1455	25.3	25.3	25.3	25.2			28.4		27.1	28.6	27.9	34
61	1456	25.3	25.3	24.9	25.2			27.5		28.2	31.1	29.7	38.1
61	1457	25.1	25	24.5	24.7			27.5		28.9	32.7	31.4	42.1
61	1458	24.9	24.7	24.5	24.2			27.5		28.5	31.8	32.1	43.7
61	1459	24.9	24.7	24.4	24.2			26.6		28.1	30.9	31.2	41.6
61	1460	24.8	24.6	24.4	23.9			26.6		26.3	26.8	29.7	38.1
61	1461	24.8	24.6	24.4	23.9			26.6		24.5	22.7	27.6	33.4
61	1462	24.8	24.6	24.4	23.9			25.7		24.3	22.1	25.8	29.2
61	1463	24.8	24.6	24.4	23.9			25.7		24	21.5	24.1	25.3
61	1464	24.8	24.6	22.6	23.9			25.7		23.7	20.8	22.7	22
62	1465	24.8	23.7	22.9	20.2					23.4	22.2	21.4	17
62	1466	25	24	24.6	20.7					23.1	21.6	20.4	14.6
62	1467	25.9	25.2	27.1	23.1					22.9	21.1	19.4	12.4
62	1468	27.3	27.2	29.2	26.9					22.7	20.6	18.6	10.5
62	1469	28.5	28.9	30.7	30					22.5	20	17.9	8.9
62	1470	29.3	30	30.7	32.2					22.1	19.3	17.3	7.5
62	1471	29.3	30	30.9	32.2					21.8	18.4	16.7	6.2
62	1472	29.4	30.1	30.9	32.4					21.4	17.7	16.3	5.2
62	1473	29.4	30.1	30.5	32.4					21.2	17.1	16.1	4.8
62	1474	29.2	29.9	30.1	31.9					20.9	16.5	16.9	6.6
62	1475	29	29.6	29.8	31.4					20.9	16.3	19.2	12
62	1476	28.8	29.3	28.9	30.8					21.1	16.9	22.8	20.2
62	1477	28.3	28.6	28.5	29.5					22.4	19.9	26.9	29.5
62	1478	28.1	28.3	28	29					24.1	23.8	30.2	37.3
62	1479	27.8	27.9	27.4	28.2					25.8	27.7	32.9	43.3
62	1480	27.5	27.5	27.1	27.4					27.2	31	34.4	46.9
62	1481	27.3	27.2	26.7	26.9					28.4	33.7	35.5	49.5
62	1482	27.1	26.9	26.4	26.3					29.4	35.9	35.7	49.9
62	1483	26.9	26.6	26.2	25.8					29.9	37	34.4	46.9
62	1484	26.8	26.5	25.8	25.5					29.1	35.4	32.7	42.9
62	1485	26.6	26.2	25.5	25					28.3	33.6	30.4	37.7
62	1486	26.4	25.9	25.3	24.5					26.3	28.9	28.4	33
62	1487	26.3	25.8	25.8	24.2					25.1	26.1	26.4	28.5
62	1488	26.6	26.2	26.1	25					24.1	23.7	24.8	24.8

63	1489	26.6	26.4	26.9	25.6					23.4	24	23.4	21.7
63	1490	27	26.9	27.6	26.7					22.8	22.7	22.2	19
63	1491	27.4	27.5	27.8	27.8					22.3	21.4	21.2	16.6
63	1492	27.5	27.6	28.5	28					21.5	19.8	20.2	14.5
63	1493	27.9	28.2	28.7	29.1					20.9	18.4	19.5	12.8
63	1494	28	28.3	29.4	29.4					20.4	17.2	18.8	11.2
63	1495	28.4	28.9	29.4	30.4					20	16.2	18.2	9.7
63	1496	28.4	28.9	29.2	30.4					19.5	15.2	17.6	8.4
63	1497	28.3	28.7	28.8	30.2					19.1	14.2	17.3	7.7
63	1498	28.1	28.5	28.7	29.6					18.8	13.4	17.8	8.9
63	1499	28	28.3	28.3	29.4					18.5	12.8	19.6	13.1
63	1500	27.8	28	27.9	28.8					18.5	12.9	23.1	21
63	1501	27.6	27.8	27.4	28.3					19.7	15.5	26.8	29.6
63	1502	27.3	27.3	27	27.5					21.4	19.5	29.7	36.2
63	1503	27.1	27.1	26.7	27					23.4	24	30.9	38.9
63	1504	26.9	26.8	26.5	26.4					25.2	28.2	31.5	40.5
63	1505	26.8	26.6	26.1	26.2					26.6	31.5	32	41.7
63	1506	26.6	26.4	25.8	25.6					27.6	33.8	32.5	42.8
63	1507	26.4	26.1	25.6	25.1					28.1	34.9	31.9	41.2
63	1508	26.3	25.9	25.2	24.8					28.1	34.8	30.7	38.5
63	1509	26.1	25.7	24.9	24.3					27.5	33.5	29.2	35.2
63	1510	25.9	25.4	24.7	23.8					26.4	31	27.3	30.7
63	1511	25.8	25.2	25.2	23.5					25.3	28.4	25.8	27.3
63	1512	26.1	25.7	26	24.3					24.3	26.2	24.7	24.7
64	1513	26.9	26.5	27.6	25.1					23.2	23.7	23.7	21.9
64	1514	27.8	27.7	29.4	27.5					22.3	21.7	22.9	20.2
64	1515	28.8	29.1	31.2	30.1					21.6	20.1	22.2	18.6
64	1516	29.8	30.5	33.2	32.8					21.1	18.9	21.6	17
64	1517	30.9	32.1	34.3	35.7					20.7	17.9	21	15.7
64	1518	31.5	32.9	34.3	37.3					20.2	16.9	20.4	14.4
64	1519	31.5	32.9	34.1	37.3					19.8	15.9	20.1	13.7
64	1520	31.4	32.8	33.4	37					19.3	14.9	19.9	13.3
64	1521	31	32.2	32.5	36					18.9	14	19.9	13.2
64	1522	30.5	31.5	31.8	34.7					18.6	13.1	20.2	14
64	1523	30.1	30.9	27.5	33.6					18.5	12.9	20.7	15.2
64	1524	27.7	27.6	22.8	27.2					18.6	13.3	22.1	18.3
64	1525	25.1	23.9	26	20.3					19.7	15.8	24.5	23.8
64	1526	26.9	26.5	26	25.1					21.7	20.3	26.8	29.1
64	1527	26.9	26.5	25.8	25.1					23.6	24.7	28.5	33
64	1528	26.8	26.3	25.3	24.8					25.3	28.7	31.1	39
64	1529	26.5	25.9	25.1	24					26.8	32	32.5	42.2
64	1530	26.4	25.8	24.9	23.7					27.7	34.1	32.9	43
64	1531	26.3	25.6	24.4	23.5					28.1	35	32.2	41.6
64	1532	26	25.2	24.2	22.7					27.6	34	30.9	38.6
64	1533	25.9	25.1	24	22.4					27.1	32.8	29.1	34.4
64	1534	25.8	24.9	23.9	22.1					26.3	30.8	27.3	30.3
64	1535	25.7	24.8	24	21.9					25.3	28.5	25.7	26.5
64	1536	25.8	24.9	25.6	22.1					24.3	26.3	24.2	23.2
65	1537	25.9	25.8	26	25.3					23.5	24.6	23.1	19.1
65	1538	26.1	26	26.7	25.9					22.7	22.8	22	16.7
65	1539	26.5	26.6	27.6	26.9					22	21.3	21.1	14.6
65	1540	27	27.3	28.3	28.3					21.4	19.9	20.3	12.8
65	1541	27.4	27.9	29.1	29.3					20.9	18.6	19.6	11.2
65	1542	27.8	28.4	29.1	30.4					20.4	17.5	18.9	9.6
65	1543	27.8	28.4	28.3	30.4					20	16.6	18.4	8.3
65	1544	27.4	27.9	28.2	29.3					19.6	15.7	17.9	7.2
65	1545	27.3	27.7	27.6	29.1					19.2	14.8	17.5	6.3
65	1546	27	27.3	27.3	28.3					18.9	14.1	18.1	7.7
65	1547	26.8	27	26.9	27.7					18.8	13.8	20.2	12.6
65	1548	26.6	26.7	26.4	27.2					19	14.4	23.7	20.5
65	1549	26.3	26.3	26	26.4					20.2	16.9	27.6	29.6
65	1550	26.1	26	25.5	25.9					21.8	20.8	31.2	37.9
65	1551	25.8	25.6	25.3	25.1					22.9	23.3	33.8	43.8
65	1552	25.7	25.5	25.1	24.8					24.5	27	35	46.5
65	1553	25.6	25.3	24.9	24.5					25.9	30.2	36	48.8

65	1554	25.5	25.2	24.6	24.3					26.8	32.2	36	48.8
65	1555	25.3	24.9	24.6	23.8					27.2	33.1	35.1	46.8
65	1556	25.3	24.9	24.2	23.8					27.1	32.9	33.7	43.7
65	1557	25.1	24.6	24.2	23.2					26.3	31.1	31.9	39.4
65	1558	25.1	24.6	24.2	23.2					25.5	29.2	30	35.1
65	1559	25.1	24.6	24.2	23.2					24.5	27	28.3	31.2
65	1560	25.1	24.6	24.7	23.2					23.8	25.2	26.9	27.9
66	1561	25.3	25	24.7	24.1					23.1	24	25.7	29.7
66	1562	25.3	25	25.1	24.1					22.5	22.6	24.6	27.2
66	1563	25.5	25.3	25.6	24.7					21.9	21.3	23.7	25.1
66	1564	25.8	25.7	26.9	25.5					21.5	20.3	23.1	23.6
66	1565	26.5	26.7	28.2	27.3					21	19.3	22.5	22.3
66	1566	27.2	27.7	28.5	29.2					20.7	18.4	21.9	21.1
66	1567	27.4	28	28.7	29.7					20.4	17.8	21.5	20
66	1568	27.5	28.1	28.5	30					20.1	17.1	21	18.9
66	1569	27.4	28	28.3	29.7					19.7	16.3	20.6	18
66	1570	27.3	27.8	28	29.5					19.4	15.5	21.1	19
66	1571	27.1	27.5	27.4	28.9					19.2	15.1	22.1	21.5
66	1572	26.8	27.1	27.1	28.1					19.3	15.3	23.3	24.2
66	1573	26.6	26.8	26.5	27.6					20.1	17.1	23.2	24.1
66	1574	26.3	26.4	26.2	26.8					20.9	18.9	22.4	22.1
66	1575	26.1	26.1	25.8	26.3					21.8	21.1	22.3	21.9
66	1576	25.9	25.9	25.5	25.7					23.6	25.2	23.2	24.1
66	1577	25.7	25.6	24.9	25.2					24.6	27.4	23.6	24.8
66	1578	25.4	25.2	24.6	24.4					25.4	29.3	23.6	24.8
66	1579	25.2	24.9	24	23.9					26	30.8	23.2	24.1
66	1580	24.9	24.5	23.8	23.1					26.2	31.3	23.1	23.8
66	1581	24.8	24.3	23.5	22.8					26	30.7	22.6	22.5
66	1582	24.6	24	23.5	22.3					25.3	29.1	21.9	20.9
66	1583	24.6	24	23.8	22.3					24.5	27.3	21.4	19.8
66	1584	24.8	24.3	24.2	22.8					23.6	25.3	21	18.9
67	1585	25.5	24.9	25.5	22.9					22.8	24.5	20.7	21.4
67	1586	26.2	25.8	27.3	24.7					22	22.6	20.4	20.7
67	1587	27.2	27.2	28.4	27.4					21.2	20.8	20.1	20.1
67	1588	27.8	28.1	29.8	29					20.7	19.5	19.6	18.9
67	1589	28.6	29.2	30.9	31.1					20.2	18.4	19	17.6
67	1590	29.2	30	30.9	32.7					19.7	17.1	18.6	16.6
67	1591	29.2	30	30.5	32.7					19.1	15.8	18.3	15.9
67	1592	29	29.8	30.2	32.2					18.6	14.6	18	15.3
67	1593	28.8	29.5	29.5	31.7					18.1	13.4	17.9	15.1
67	1594	28.4	28.9	28.9	30.6					17.6	12.5	18.2	15.6
67	1595	28.1	28.5	28.4	29.8					17.4	11.9	18.9	17.2
67	1596	27.8	28.1	28	29					17.5	12.1	19.8	19.4
67	1597	27.6	27.8	27.5	28.5					18.4	14.2	20.2	20.4
67	1598	27.3	27.4	26.8	27.7					19.9	17.6	20.6	21.2
67	1599	26.9	26.8	26.4	26.6					21.6	21.5	22.2	25
67	1600	26.7	26.5	25.9	26.1					23.2	25.2	24.2	29.6
67	1601	26.4	26.1	25.5	25.3					24.6	28.6	24.4	29.9
67	1602	26.2	25.8	25	24.7					25.7	30.9	23.2	27.3
67	1603	25.9	25.4	24.6	23.9					26.2	32.2	21.9	24.3
67	1604	25.7	25.1	24.1	23.4					26.2	32.2	20.9	21.8
67	1605	25.4	24.7	23.9	22.6					25.8	31.3	20.1	19.9
67	1606	25.3	24.6	23.9	22.3					25	29.5	19.2	18.1
67	1607	25.3	24.6	24.1	22.3					24.1	27.3	18.5	16.5
67	1608	25.4	24.7	24.7	22.6					23.2	25.3	18	15.3
68	1609	26	25.3	25.6	23.2					22.5	22.4	17.6	14
68	1610	26.5	26	26.8	24.5					21.8	20.8	17.4	13.4
68	1611	27.2	27	28.1	26.4					21.2	19.3	17.1	12.8
68	1612	27.9	28	29.7	28.3					20.6	18	16.9	12.2
68	1613	28.8	29.2	30.6	30.6					20	16.7	16.4	11.2
68	1614	29.3	29.9	30.8	32					19.5	15.6	15.9	9.9
68	1615	29.4	30.1	30.6	32.2					19.1	14.6	15.4	8.9
68	1616	29.3	29.9	30.6	32					18.7	13.7	15.3	8.6
68	1617	29.3	29.9	30.1	32					18.3	12.8	15.2	8.5
68	1618	29	29.5	29.7	31.2					18	12.1	15.5	9.1

68	1619	28.8	29.2	29.3	30.6					17.8	11.7	17.2	13
68	1620	28.6	29	28.8	30.1					18	12	19.7	18.9
68	1621	28.3	28.5	28.3	29.3					19	14.4	22.2	24.6
68	1622	28	28.1	27.7	28.5					21.1	19.1	23.9	28.5
68	1623	27.7	27.7	27.2	27.7					23.3	24.1	25.8	32.8
68	1624	27.4	27.3	27	26.9					25.3	28.7	26.2	33.7
68	1625	27.3	27.1	26.3	26.7					26.9	32.5	27	35.5
68	1626	26.9	26.6	26.1	25.6					27.6	34	27.9	37.7
68	1627	26.8	26.4	25.7	25.3					28.1	35.2	26.3	34
68	1628	26.6	26.2	25.4	24.8					28.3	35.7	24.7	30.4
68	1629	26.4	25.9	25.2	24.3					27.9	34.9	23.3	27.1
68	1630	26.3	25.7	25	24					27.2	33.2	22	24
68	1631	26.2	25.6	25.6	23.7					26.2	30.8	21	21.7
68	1632	26.5	26	25.9	24.5					25.1	28.4	19.9	19.3
69	1633	27	26.5	27.2	24.7					24.2	24.8	19	17.9
69	1634	27.7	27.4	28.4	26.6					23.4	22.9	18.4	16.6
69	1635	28.4	28.4	30	28.4					22.8	21.3	17.9	15.3
69	1636	29.3	29.7	30.9	30.8					22.1	19.9	17.2	13.7
69	1637	29.8	30.4	32	32.2					21.6	18.6	16.5	12.1
69	1638	30.4	31.2	32	33.8					21.1	17.5	15.9	10.7
69	1639	30.4	31.2	31.8	33.8					20.6	16.5	15.4	9.5
69	1640	30.3	31.1	31.3	33.5					20.2	15.5	15	8.8
69	1641	30	30.7	30.9	32.7					19.8	14.7	15	8.8
69	1642	29.8	30.4	30.6	32.2					19.5	13.9	15.3	9.4
69	1643	29.6	30.1	30	31.6					19.4	13.6	15.9	10.7
69	1644	29.3	29.7	29.7	30.8					19.5	13.8	16.5	12.3
69	1645	29.1	29.4	29.1	30.3					20.2	15.4	18.2	16
69	1646	28.8	29	28.2	29.5					21.9	19.4	20.9	22.2
69	1647	28.3	28.3	27.7	28.2					23.9	24.1	24.2	29.8
69	1648	28	27.9	27.2	27.4					25.9	28.6	26.4	34.9
69	1649	27.7	27.4	26.6	26.6					27.5	32.3	25.9	33.8
69	1650	27.4	27	25.9	25.8					28.6	34.7	25.4	32.6
69	1651	27	26.5	25.5	24.7					29.1	36	24.9	31.5
69	1652	26.8	26.2	25	24.2					29.2	36.2	24.5	30.5
69	1653	26.5	25.8	24.8	23.4					28.9	35.4	23.5	28.2
69	1654	26.4	25.6	24.6	23.1					28.2	33.8	22.4	25.7
69	1655	26.3	25.5	25.2	22.9					27.4	31.9	21.4	23.5
69	1656	26.6	25.9	26	23.7					26.5	30	20.6	21.5
70	1657	26.8	26.4	26.9	25.1					25.8	27.3	19.9	20.9
70	1658	27.3	27.1	28	26.4					25.2	25.9	19.4	19.6
70	1659	27.9	27.9	28.9	28					24.6	24.6	19	18.7
70	1660	28.4	28.6	29.8	29.4					24.1	23.3	18.7	18
70	1661	28.9	29.3	30.7	30.7					23.6	22.2	18.4	17.3
70	1662	29.4	30	30.8	32					23.2	21.3	18.1	16.7
70	1663	29.5	30.2	30.5	32.3					22.8	20.4	17.9	16.3
70	1664	29.3	29.9	30.1	31.7					22.4	19.5	17.8	16
70	1665	29.1	29.6	29.8	31.2					22.1	18.8	17.8	16
70	1666	28.9	29.3	29.6	30.7					21.7	18	18.2	16.9
70	1667	28.8	29.2	29.2	30.4					21.7	17.8	18.7	18
70	1668	28.6	28.9		29.9					21.7	17.9	18.9	18.6
70	1669	28.3	28.5		29.1					21.8	18.1	19	18.7
70	1670	28	28.1		28.3					22.1	18.8	19.2	19.2
70	1671	27.8	27.8		27.8					23.1	21.2	19.2	19.3
70	1672	27.6	27.5		27.2					25.1	25.8	19.4	19.6
70	1673	27.3	27.1		26.4					26.8	29.6	19.5	19.9
70	1674	27.1	26.8		25.9					28	32.4	19.8	20.6
70	1675	26.9	26.5		25.4					28.5	33.6	20.6	22.3
70	1676	26.7	26.3		24.8					28.5	33.5	20.6	22.5
70	1677	26.4	25.8		24					27.9	32.2	20.4	22
70	1678	26.3	25.7		23.8					27.4	30.9	20.2	21.6
70	1679	26.2	25.6		23.5					26.7	29.4	20	21
70	1680	26.3	25.7		23.8					26.1	28	19.9	20.7
71	1681	26.6	25.9		23.7					25.6	26.8	19.7	17.8
71	1682	27.3	26.9		25.6					25.2	25.8	19.4	17.2
71	1683	27.9	27.7		27.2					24.8	25.1	19.2	16.6

71	1684	28.8	29		29.6					24.5	24.3	19	16.2
71	1685	29.8	30.4		32.2					24.1	23.4	18.7	15.5
71	1686	30.3	31.1		33.6					23.8	22.6	18.3	14.6
71	1687	30.2	31		33.3					23.4	21.8	18.2	14.3
71	1688	30	30.7		32.8					23.1	21.1	18	14
71	1689	29.8	30.4		32.2					22.9	20.5	18	13.9
71	1690	29.6	30.1		31.7					22.7	20	18.2	14.5
71	1691	29.4	29.8		31.2					22.6	19.8	19	16.3
71	1692	29.1	29.4		30.4					22.7	20.2	19.9	18.4
71	1693	28.8	29		29.6					23.2	21.2	22	23.1
71	1694	28.5	28.6		28.8					24	23	24.6	29.2
71	1695	28.3	28.3		28.3					24.5	24.2	25.5	31.2
71	1696	28.1	28		27.7					25.3	26.1	26.1	32.5
71	1697	27.8	27.6		26.9					26.1	28	26.1	32.5
71	1698	27.6	27.3		26.4					26.5	28.7	25	30
71	1699	27.4	27		25.9					26.8	29.5	24	27.7
71	1700	27.2	26.8		25.3					27	30.1	23.2	25.8
71	1701	26.9	26.3		24.5					27	29.9	22.4	24
71	1702	26.8	26.2		24.3					26.6	29	21.6	22.1
71	1703	26.8	26.2		24.3					25.8	27.3	20.8	20.4
71	1704	26.8	26.2		24.3					24.7	24.8	20.3	19.2
72	1705	27.1	26.5							24	24.7	19.8	16.5
72	1706	27.5	27.1							23.5	23.6	18.9	14.5
72	1707	28.2	28							23.1	22.6	18	12.5
72	1708	28.8	28.9							22.8	21.9	17.2	10.6
72	1709	29.8	30.3							22.5	21.2	16.4	8.7
72	1710	30.2	30.8							22.2	20.6	15.7	7.2
72	1711	30.3	31							21.9	19.8	15.2	6
72	1712	30.2	30.8							21.5	18.9	14.9	5.1
72	1713	30	30.6							21.2	18.4	14.5	4.4
72	1714	29.8	30.3							21	17.9	14.9	5.1
72	1715	29.6	30							21	17.8	16.5	9
72	1716	29.3	29.6							21.2	18.2	19.5	15.8
72	1717	29.1	29.3							21.7	19.4	22.6	23
72	1718	28.8	28.9							22.3	20.8	25.7	30.2
72	1719	28.6	28.6							23.1	22.6	28.8	37.2
72	1720	28.4	28.3							24.3	25.4	31	42.2
72	1721	28.3	28.2							24.8	26.6	32.4	45.4
72	1722	28.1	27.9							25.8	28.8	32.7	46.1
72	1723	27.8	27.5							26.5	30.5	31.3	43
72	1724	27.7	27.3							26.7	30.9	29.6	39.1
72	1725	27.5	27.1							26.6	30.7	27.6	34.3
72	1726	27.3	26.8							25.9	29	25.7	30.2
72	1727	27.3	26.8							25.2	27.4	24.1	26.3
72	1728	27.2	26.6							24.6	26	22.7	23.1
73	1729	27.1	27.5							23.9	24.3	21.7	19.7
73	1730	27.1	27.5							23.3	22.9	20.9	17.9
73	1731	26.9	27.2							22.8	21.8	20.2	16.3
73	1732	26.8	27.1							22.3	20.8	19.7	15.1
73	1733	26.5	26.7							22	20	19.2	13.9
73	1734	26.8	27.1							21.7	19.2	18.7	12.7
73	1735	27.1	27.5							21.3	18.5	18.3	11.9
73	1736	27.1	27.5							21.1	17.9	17.9	11
73	1737	27.1	27.5							20.8	17.2	17.7	10.6
73	1738	27.1	27.5							20.6	16.7	18.1	11.5
73	1739	26.9	27.2							20.4	16.4	19.2	13.9
73	1740	26.8	27.1							20.5	16.5	20.7	17.4
73	1741	26.5	26.7							21.3	18.4	22.4	21.4
73	1742	26.3	26.4							22.8	21.7	25.1	27.6
73	1743	25.9	25.8							24.4	25.6	27.2	32.3
73	1744	25.8	25.7							25.9	29.1	29.1	36.8
73	1745	25.4	25.1							27	31.6	30	38.8
73	1746	25.2	24.9							27.8	33.2	30.2	39.2
73	1747	24.9	24.4							28.3	34.4	30	38.8
73	1748	24.8	24.3							27.3	32.1	29.1	36.6

73	1749	24.4	23.7							26.1	29.4	27.6	33.3
73	1750	24.4	23.7							25.2	27.3	26.1	29.9
73	1751	24.2	23.5							24.5	25.7	24.8	26.8
73	1752	24.3	23.6							23.8	24.2	23.7	24.3
74	1753	24.3								23.2	21.4	22.8	25.4
74	1754	24.9								22.6	20	22.1	23.8
74	1755	25.5								22.1	18.8	21.4	22.2
74	1756	26.2								21.7	17.9	20.9	21
74	1757	26.9								21.4	17.3	20.2	19.5
74	1758	27.3								21.3	16.9	19.7	18.2
74	1759	27.3								21.1	16.6	19.2	17.1
74	1760	27.1								21	16.3	18.7	16.1
74	1761	26.8								20.7	15.7	18.4	15.2
74	1762	26.8								20.6	15.3	18.3	15.1
74	1763	26.6								20.6	15.4	18.8	16.2
74	1764	26.3								20.9	16.2	19.7	18.2
74	1765	25.9								21.7	17.9	20.5	20.1
74	1766	25.8								23.6	22.3	21.1	21.4
74	1767	25.4								25.7	27	21.7	22.8
74	1768	25.2								27.6	31.4	22.2	24.1
74	1769	24.9								29	34.8	23.2	26.4
74	1770	24.7								30	36.9	23.5	27
74	1771	24.4								30.5	38.1	23.1	26.1
74	1772	24								30.4	38	22.6	25
74	1773	23.9								29.9	36.7	21.7	23
74	1774	23.6								29	34.7	20.7	20.5
74	1775	23.5								28	32.5	19.7	18.2
74	1776	23.4								27.1	30.2	18.9	16.3
75	1777										27.9	17.9	14.1
75	1778										26.2	17.2	12.4
75	1779										24.6	16.5	10.8
75	1780										23.1	15.8	9.2
75	1781										21.8	15.2	7.9
75	1782										20.6	14.7	6.6
75	1783										19.4	14.2	5.5
75	1784										18.4	13.7	4.3
75	1785										17.4	13.4	3.6
75	1786										16.5	13.7	4.3
75	1787										15.9	15.6	8.8
75	1788										15.8	19	16.6
75	1789										17.1	22	23.5
75	1790										20.8	24.6	29.4
75	1791										24.8	27	35
75	1792										28.6	28.8	39.2
75	1793										31.6	29.8	41.5
75	1794										34	29.6	40.9
75	1795										35	28.8	39.2
75	1796										35	27.7	36.7
75	1797										33.8	26.1	32.8
75	1798										31.9	24.4	29.1
75	1799										29.7	22.9	25.6
75	1800										27.6	21.8	23.1
76	1801										27.1	20.7	17.7
76	1802										25.5	20.1	16.1
76	1803										24	19.6	15
76	1804										22.6	19.2	14.2
76	1805										21.3	18.9	13.4
76	1806										20.1	18.5	12.7
76	1807										19.1	18.2	11.9
76	1808										18.1	18	11.4
76	1809										17.2	17.9	11.1
76	1810										16.5	18	11.5
76	1811										16.5	19.4	14.7
76	1812										17	21.7	19.9
76	1813										18	23.6	24.2

76	1814										21.7	25.2	28.1
76	1815										23.4	27.9	34.1
76	1816										24.9	29.4	37.7
76	1817										27.8	30.1	39.1
76	1818										30.1	29.6	38.1
76	1819										32	29.2	37.3
76	1820										32.3	28.4	35.4
76	1821										31.7	27	32.1
76	1822										30.2	25.6	28.9
76	1823										28.4	24.4	26
76	1824										26.7	23.2	23.5
77	1825										26.5		23.2
77	1826										25.1		21.3
77	1827										24		19.4
77	1828										23		17.8
77	1829										22		17
77	1830										21.1		16.4
77	1831										20.2		16
77	1832										19.4		15.4
77	1833										18.7		14.7
77	1834										18.1		15
77	1835										17.8		16.4
77	1836										17.7		19.3
77	1837										18.1		21.4
77	1838										19.1		23.6
77	1839										20.9		24.9
77	1840										23.3		26.9
77	1841										25.8		28.6
77	1842										27.1		28.8
77	1843										28.5		28.5
77	1844										28.7		27.8
77	1845										27.9		26.5
77	1846										26.7		24.9
77	1847										25.3		23.5
77	1848										24.2		22.2
78	1849										23.3		19.8
78	1850										22.1		19
78	1851										21		18.2
78	1852										20		17.7
78	1853										19.1		17.1
78	1854										18.5		16.5
78	1855										18.1		15.8
78	1856										17.5		14.9
78	1857										16.8		14.2
78	1858										16.2		14.5
78	1859										15.9		15.4
78	1860										16		16.9
78	1861										16.8		19.4
78	1862										19.2		21.7
78	1863										22.4		23.4
78	1864										25.6		28.3
78	1865										28.3		33.2
78	1866										30.2		35.2
78	1867										31.3		35.1
78	1868										31.3		33.3
78	1869										30.5		31.5
78	1870										29		29.3
78	1871										27.5		27.6
78	1872										25.9		25.7
79	1873										24.2		20.8
79	1874										23		19.1
79	1875										21.8		17.6
79	1876										20.7		16.3
79	1877										19.7		15
79	1878										18.8		14

79	1879										18		13.2
79	1880										17.2		13
79	1881										16.4		12.9
79	1882										15.8		13
79	1883										15.6		15.6
79	1884										15.7		19.1
79	1885										15.9		23.7
79	1886										16.8		30.4
79	1887										19.7		32.7
79	1888										23.4		36.9
79	1889										27.1		40.5
79	1890										30.2		41.9
79	1891										32.2		40.3
79	1892										33		37.6
79	1893										32.6		35
79	1894										31.4		32.4
79	1895										29.7		30.4
79	1896										28.1		28.7
80	1897										24.6		
80	1898										23.4		
80	1899										22.2		
80	1900										21.3		
80	1901										20.5		
80	1902										19.9		
80	1903										19.2		
80	1904										18.5		
80	1905										17.7		
80	1906										17.1		
80	1907										16.5		
80	1908										16.2		
80	1909										17.2		
80	1910										20.4		
80	1911										24.1		
80	1912										27.7		
80	1913										30.6		
80	1914										32.8		
80	1915										34		
80	1916										34.2		
80	1917										33.7		
80	1918										32.3		
80	1919										30.5		
80	1920										28.6		
81	1921										26.4		
81	1922										24.9		
81	1923										23.6		
81	1924										22.4		
81	1925										21.3		
81	1926										20.2		
81	1927										19.2		
81	1928										18.3		
81	1929										17.4		
81	1930										16.6		
81	1931										16.1		
81	1932										16		
81	1933										16.9		
81	1934										20.1		
81	1935										23.9		
81	1936										27.7		
81	1937										31.1		
81	1938										33.6		
81	1939										35		
81	1940										35.4		
81	1941										34.6		
81	1942										32.9		
81	1943										30.7		

81	1944										28.7		
82	1945										26.3		
82	1946										24.7		
82	1947										23.4		
82	1948										22.5		
82	1949										21.8		
82	1950										21.1		
82	1951										20.5		
82	1952										20		
82	1953										19.5		
82	1954										19		
82	1955										18.6		
82	1956										18.6		
82	1957										19		
82	1958										20		
82	1959										22.8		
82	1960										26.6		
82	1961										29.9		
82	1962										32.5		
82	1963										34.1		
82	1964										34.5		
82	1965										33.9		
82	1966										32.3		
82	1967										30.4		
82	1968										28.5		
